# Ultrasound-activated dual-targeted liposomes for visualized precise neuromodulation against myocardial ischemia-reperfusion injury

**DOI:** 10.1016/j.mtbio.2026.102952

**Published:** 2026-02-24

**Authors:** Haoyuan Hu, Weiqin Yao, Huijun Wu, Qian Li, Wei Guo, Yida Pang, Hong Jiang, Yao Sun, Wei-Hai Chen, Songyun Wang

**Affiliations:** a Cardiovascular Hospital, Renmin Hospital of Wuhan University, Cardiac Autonomic Nervous System Research Center of Wuhan University, Cardiovascular Research Institute, Wuhan University, Hubei Key Laboratory of Cardiology, Wuhan, 430060, PR China; b Key Laboratory of Biomedical Polymers of Ministry of Education and Department of Chemistry, Wuhan University, Wuhan, 430072, PR China; c Key Laboratory of Pesticides and Chemical Biology, Ministry of Education, International Joint Research Center for Intelligent Biosensor Technology and Health, College of Chemistry, Central China Normal University, Wuhan, 430079, PR China; d College of Biomedicine and Health, Huazhong Agricultural University, Hubei Jiangxia Laboratory, Wuhan, 430070, PR China

**Keywords:** Liposomes, Dual-targeted, Imaging, Neuroinflammation, Sonosensitizer, Myocardial ischemia-reperfusion injury

## Abstract

Myocardial ischemia-reperfusion (I/R) injury remains a critical clinical challenge with limited effective treatments. Sympathetic hyperactivation and microglia-mediated neuroinflammation in the paraventricular nucleus exacerbate myocardial I/R injury. Whilst previous studies suggest that sonodynamic neuromodulation has the potential to impede neuroinflammation and confer cardioprotective effect, existing sonosensitizers employed in neuromodulation demonstrate deficiencies in terms of cellular targeting specificity and the capacity to facilitate functional imaging of neuroinflammation. Herein, a sonosensitizer (BT) was designed based on a donor-acceptor-donor scaffold exhibiting emission in the near-infrared (NIR)-II window, and it was further engineered into a neuroinflammatory dual-targeted sonosensitizer through antibody-modified liposomes (named BT@Lip-TN). *In vitro* studies demonstrated that BT@Lip-TN under ultrasound irradiation significantly enhanced reactive oxygen species generation compared to commercial sonosensitizers. More importantly, BT@Lip-TN was selectively internalized by sympathetic neurons and microglia, localized to mitochondria, and promoted mitophagy via the PINK1-Parkin pathway, thereby modulating neuroinflammation. *In vivo* studies confirmed that BT@Lip-TN enabled functional NIR-II imaging and real-time monitoring of neuroinflammatory activity. Furthermore, BT@Lip-TN-mediated targeted sonodynamic neuromodulation suppressed sympathetic neuroinflammation and ameliorating myocardial I/R injury. This study pioneers the design of neuroinflammatory dual-targeted sonosensitizer and establishes an integrated theranostic platform, offering novel insights into visualized precise neuromodulation and the treatment of myocardial I/R injury.

## Graphical abstract

**Figure:**
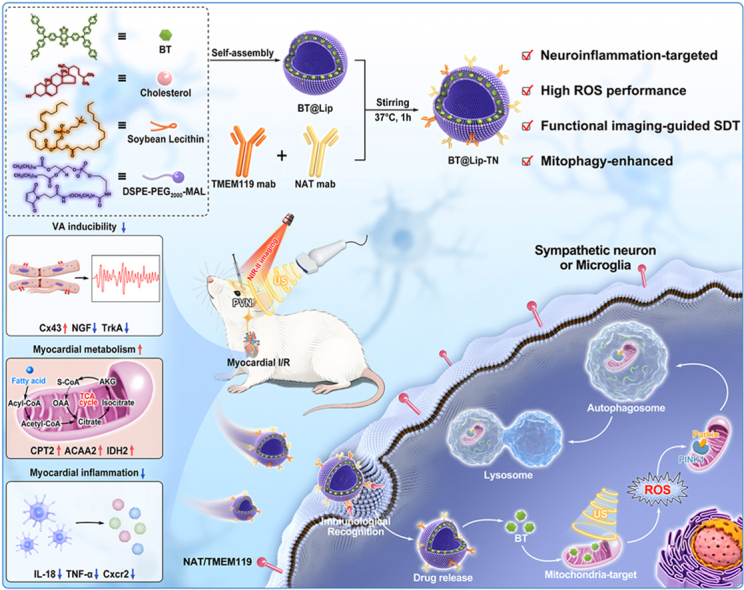

## 1 Introduction

Although restoring coronary blood flow via percutaneous coronary intervention or thrombolytic therapy remains the cornerstone strategy for improving clinical outcomes in acute myocardial infarction, subsequent myocardial ischemia-reperfusion (I/R) injury and associated ventricular arrhythmias (VAs) persist as major unresolved clinical challenges [[1], [2], [3]]. Substantial evidence has confirmed that excessive activation of the sympathetic nervous system and inflammatory responses, constitutes the fundamental pathophysiological mechanisms underpinning myocardial I/R injury [4,5]. The paraventricular nucleus (PVN) of the hypothalamus is the crucial integrative center for sympathetic nervous activity and neuroimmune responses. It harbors a self-amplifying vicious cycle formed by microglial activation and excessive sympathetic excitation [6]. More crucially, aberrant neural signals of the PVN transmit through its downstream neural circuit, particularly via the left stellate ganglion (LSG), thereby significantly exacerbating myocardial I/R injury. While surgical interventions such as LSG resection or blockade demonstrate efficacy in mitigating ischemia-related VAs, their inherent invasiveness, non-specificity, and irreversible nature profoundly hinder their further clinical translation [[7], [8], [9]]. Consequently, developing novel neuromodulation strategies that concurrently achieve non-invasiveness, targeting, reversibility, and theranostic capability represents a pivotal direction for overcoming current therapeutic bottlenecks in myocardial I/R injury.

Sonodynamic therapy (SDT), leveraging its inherent non-invasiveness, deep tissue penetrability, and spatio-temporal controllability, has emerged as a promising therapeutic modality for cardiovascular diseases [10,11]. With advancements in modern imaging techniques, imaging-guided SDT systems are actively being investigated to optimize SDT efficacy assessment and treatment process monitoring, holding clear-cut advantages including high spatial resolution, rapid feedback, and no ionizing radiation [[12], [13], [14]]. Critically, the integration of sonosensitizers with fluorescence imaging capabilities represents an emerging research frontier, establishing the rational design of theranostic SDT platforms as a significant paradigm shift in this field [[15], [16], [17]]. Previous studies have indicated that SDT could exert a regulatory effect on sympathetic neuroinflammation, thereby preventing the occurrence of myocardial ischemia-associated VAs [[18], [19], [20]]. However, existing sonosensitizers based on small molecules or supramolecular structures generally exhibit negligible intrinsic capacity for active targeting towards the neuroinflammatory microenvironment. This non-specific distribution not only poses a significant risk of off-target cytotoxicity to peri-lesional healthy tissues, but also limits the effectiveness of SDT in achieving precise interventions within complex neural circuits. Additionally, non-targeted sonosensitizers and fluorophores are inherently challenging to be specifically taken up and enriched by target cells, thus rendering real-time, precise tracing and functional imaging of the activated state of neuroinflammation unfeasible. Collectively, these limitations present considerable impediments to the establishment of a visualizable, individualized platform for precise neuromodulation.

Therefore, designing sonosensitizers that combine neuroinflammation dual-targeting and integrate diagnostic and therapeutic performances is key to achieve efficient and precise sonodynamic neuromodulatory system. Liposomes, as well-established nanocarriers in drug delivery, possess distinct advantages such as exceptional biocompatibility, flexible surface modifiability, and efficient encapsulation capacity for diverse therapeutic/diagnostic agents [[21], [22], [23]]. These attributes have propelled their successful deployment in nucleic acid therapeutics, targeted small-molecule delivery, and theranostic nanoplatform construction [24]. Notably, antibody-functionalized liposomes engineered through covalent surface modification provide a robust strategy to overcome the targeting deficiencies of conventional sonosensitizers by enabling specific recognition of neuroinflammatory biomarkers, thereby establishing an optimized platform for precise sonodynamic neuromodulation [25,26]. During myocardial I/R injury, the excessive activation of sympathetic neurons and microglia constitutes a vicious cycle, and simultaneously targeting both cell types may exploit synergistic effects. Noradrenaline transporter (NAT) and transmembrane protein 119 (TMEM119) are cell-surface antigens specifically expressed on sympathetic neurons and microglia, respectively [27,28]. Therefore, constructing liposomes dually targeted to NAT and TMEM119 may serve as a promising strategy for the precise modulation of sympathetic neuroinflammation.

**Scheme 1:**
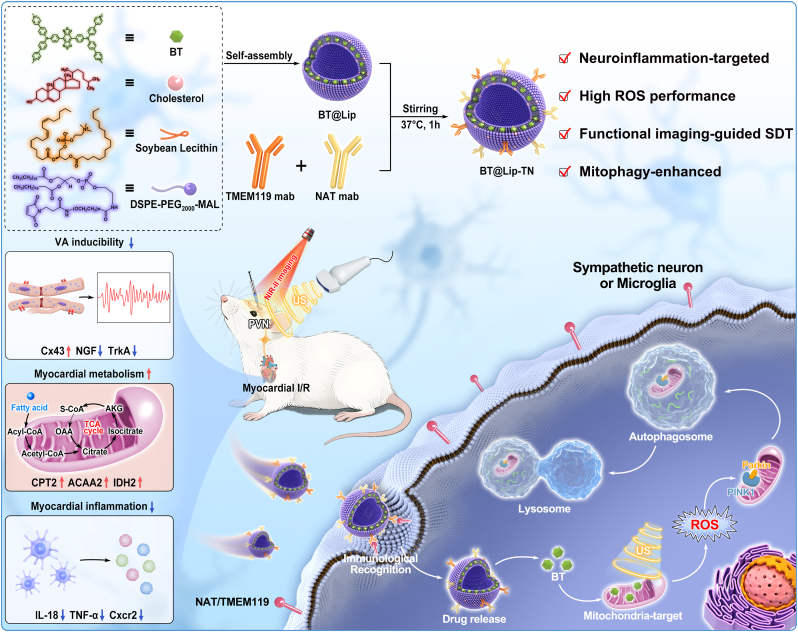
Schematic illustration of the synthesis, performance, targeted neuromodulation mechanism of BT@Lip-TN, and its biological application for protecting against myocardial I/R injury.

Herein, we designed and synthesized a near-infrared-II (NIR-II)-emissive sonosensitizer (named BT) using benzo [1,2-c:4,5c′]bis [1,2,5]thiadiazole (BBTD) as the electron acceptor and triphenylamine (TPA) as the electron donor. To enable neuroinflammation-targeting, liposomes with antibody modification sites were constructed through Michael addition reactions. Subsequently, we modified the surface of liposomes with terminal maleimide groups using sympathetic nerve-specific antibody (anti-NAT) and microglia-specific monoclonal antibody (anti-TMEM119) to construct an ultrasound-activated dual-targeted liposomes named BBTD-TPA@Liposome-TMEM119/NAT (BT@Lip-TN) with integrated NIR-II fluorescence imaging capability. *In vitro* tests confirmed that BT@Lip-TN exhibits excellent stability and undergoes specific cellular internalization in sympathetic neurons and microglia. Under ultrasound (US) activation, BT@Lip-TN demonstrated superior reactive oxygen species (ROS) generation performance and could selectively target mitochondria to induce cellular mitophagy. *In vivo* studies further validated its capacity for real-time functional imaging of neuroinflammatory activity within the PVN region post-myocardial ischemia, and BT@Lip-TN-mediated SDT can suppress neuroinflammation and protect against myocardial I/R injury by ameliorating cardiomyocyte metabolism and attenuating inflammatory responses (Scheme 1). As the first neuroinflammation dual-targeted sonosensitizer, BT@Lip-TN integrates NIR-II fluorescence imaging guidance, antibody-mediated precision delivery, ultrasound-triggered ROS generation, and mitophagy induction. The current theranostic platform establishes a novel strategy for visualizable precise neuromodulation and presents significant clinical translation potential for myocardial I/R injury management.

## 2 Results and discussion

### 2.1 Design, synthesis, and characterization of BT@Lip-TN

**Fig. 1:**
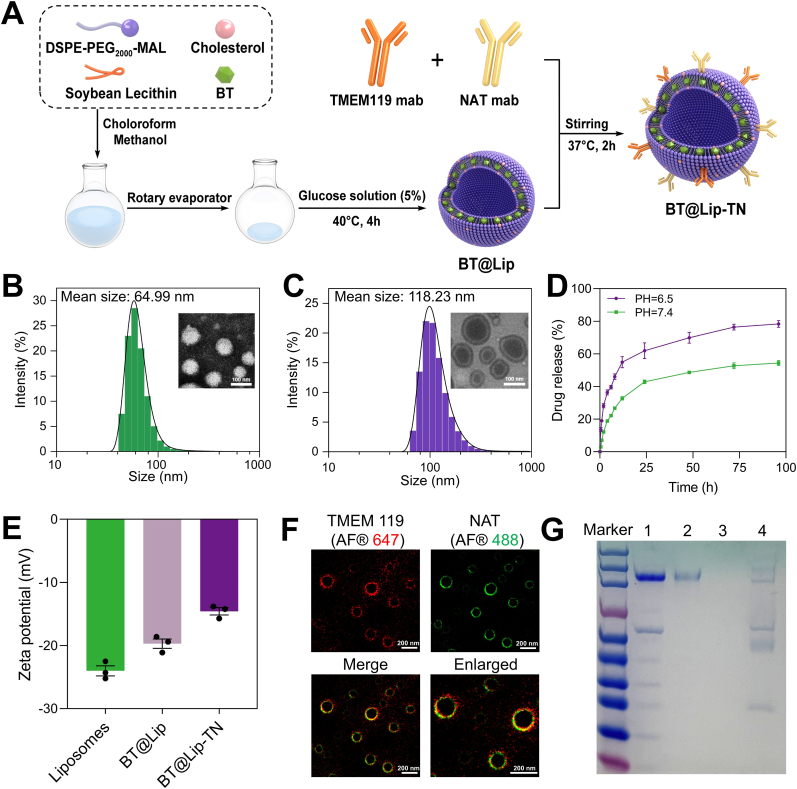
Preparation and characterization of BT@Lip and BT@Lip-TN. A) Synthetic illustration of BT@Lip-TN. B, C) Dynamic light scattering (DLS) and TEM images of empty liposomes and BT@Lip-TN showing their morphology and size. Scale bar: 100 nm. D) BT release profiles from BT@Lip under physiological condition (pH = 7.4) and inflammatory acidic microenvironment (pH = 6.5). E) Zeta potential measurements of blank liposomes, BT@Lip, and BT@Lip-TN. F) Super-resolution microscopy confirming co-localization of TMEM119 mab conjugated with Alexa Fluor® 647 (red, λ_ex_ = 652 nm, λ_em_ = 668 nm) and NAT mab conjugated with Alexa Fluor® 488 (green, λ_ex_ = 495 nm, λ_em_ = 519 nm) on the surface of BT@Lip. Scale bar: 200 nm. G) Images of reducing SDS-PAGE electrophoresis. Lane 1-4: TMEM119 mAb, NAT mAb, BT@Lip, and BT@Lip-TN. Values are expressed as mean ± S.E.M. (*n* = 3).

The organic sonosensitizer BT based on a donor-acceptor-donor (D-A-D) scaffold was synthesized via a four-step route (Fig. S1). Subsequently, BT@Lip consisting of BT, lecithin, cholesterol and DSPE-PEG_2000_-MAL were prepared by a thin-film hydration method (Fig. 1A). The encapsulation efficiency and drug-loading ratio of BT@Lip prepared at varying feed ratios of BT were quantitatively assessed, enabling the identification of the optimal BT input proportion for subsequent experimentation (Table S1). As shown in Fig. 1B, C and Figs. S2–S3, the mean hydrodynamic diameter of the synthesized liposomal formulations (BT@Lip and BT@Lip-TN) exhibited minimal variation when incubated in phosphate-buffered saline (PBS) and 10% fetal bovine serum (FBS) over a 7-day observation period, thereby evidencing robust long-term stability under physiologically relevant conditions. Meanwhile, as illustrated in Fig. 1D, BT@Lip exhibited a significantly higher drug-release efficiency at pH = 6.5 than at pH = 7.4, demonstrating that BT@Lip could achieve site-specific, high-efficiency release in acidic inflammatory lesions. Thereafter, following surface modification with TMEM119 and NAT antibodies, the zeta potential of BT@Lip changed from −24 mV to −13.5 mV, indicating that the TMEM119 and NAT antibodies were successfully modified on the surface of BT@Lip (Fig. 1E). Furthermore, super-resolution imaging provided direct visual evidence. It was observed that the red fluorophore-conjugated TMEM119 mAb and the green fluorophore-conjugated NAT mAb co-localized on the surface of BT@Lip (Fig. 1F). This confirmed the successful dual modification of both antibodies. Reducing sodium dodecyl sulfate-polyacrylamide gel electrophoresis (SDS-PAGE) demonstrated that BT@Lip-TN exhibited characteristic bands similar to those of TMEM mab and NAT mab, whereas no distinct bands were observed for BT@Lip, further verifying the successful conjugation of antibodies on the surface of BT@Lip (Fig. 1G).

### 2.2 Photophysical property and sonodynamic performance of BT@Lip-TN

**Fig. 2:**
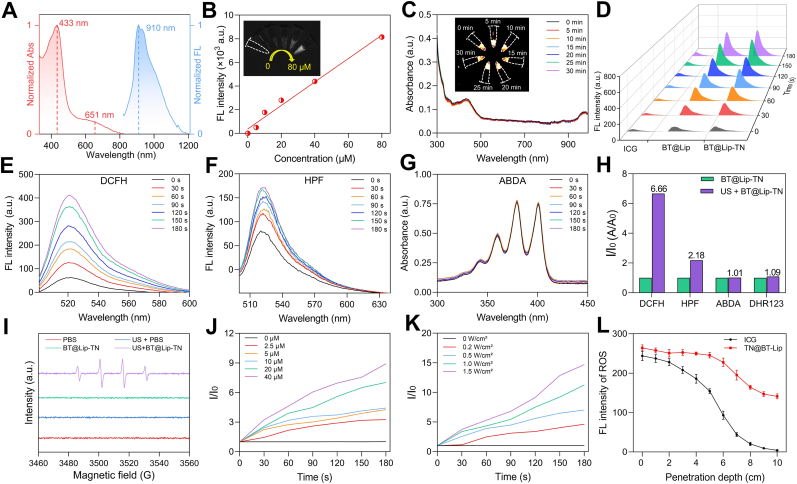
Assessment of photophysical and sonodynamic properties of BT@Lip-TN. A) Normalized absorption and NIR-II emission (λ_ex_ = 808 nm) spectra of BT@Lip-TN. B) Fluorescence fitting curves and NIR-II fluorescence images of BT@Lip-TN at different concentrations under 808 nm laser excitation. C) Absorption spectra of BT@Lip-TN under ultrasound (0.5 W cm^−2^, 1 MHz) at different time points with fluorescence signals inserted. D) Evaluation of ROS generation performance of ICG, BT@Lip, and BT@Lip-TN (20 μM) under US irradiation (0.5 W cm^−2^, 1 MHz) (indicator: DCFH). Detection of E) total ROS via DCFH probe, F) •OH via HPF probe, and G) ^1^O_2_ via ABDA probe by BT@Lip-TN (20 μM) under US irradiation. H) Quantitative analysis of various ROS generation using different probes. I) ESR spectra with DMPO as the spin trap confirming •OH signals. J) Linear curves of fluorescence intensity of DCFH with different concentrations of BT@Lip-TN (0 μM, 2.5 μM, 5 μM, 10 μM, 20 μM, and 40 μM) under US irradiation (0.5 W cm^−2^). K) The linear curves showing ROS generation performance of BT@Lip-TN (20 μM) under different ultrasound intensities (0 W cm^−2^, 0.2 W cm^−2^, 0.5 W cm^−2^, 1.0 W cm^−2^, and 1.5 W cm^−2^) (DCFH as the indicator). L) Quantitative analysis of depth-dependent ROS fluorescence intensity of BT@Lip-TN and ICG under US irradiation. Data are presented as mean ± S.E.M. (*n* = 3).

Furthermore, we evaluated the photophysical and sonodynamic properties of BT@Lip and BT@Lip-TN. BT@Lip-TN exhibited characteristic absorption peaks at 433 nm and 651 nm, with a maximum NIR-II emission peak at approximately 910 nm (Fig. 2A), demonstrating a relative large Stokes shift, which guaranteed superior NIR-II imaging for deep-tissue. The unmodified BT@Lip without monoantibodies also showed similar absorption and NIR-II emission peaks (Fig. S4). Under 808 nm excitation, the fluorescence intensity of BT@Lip-TN presented a strong linear correlation with its concentration, emphasizing its promise for quantitative fluorescence imaging applications (Fig. 2B). Additionally, BT@Lip-TN exhibited excellent stability under both US and laser irradiation, which is essential for reliable imaging and therapeutic applications. Under continuous US exposure at various intensities (0.2 W cm^−2^, 0.5 W cm^−2^, 1.0 W cm^−2^, and 1.5 W cm^−2^), the absorption spectrum and fluorescence signal of BT@Lip-TN remained relatively stable (Fig. 2C and Fig. S5). Furthermore, no significant attenuation in the absorption spectra was observed after 808 nm laser irradiation for 30 min (Fig. S6). Taken together, these results confirm the high stability of BT@Lip-TN.

Subsequently, we assessed the *in vitro* sonodynamic performance of BT@Lip and BT@Lip-TN using the commercial sonosensitizer indocyanine green (ICG) as a control. Using 2′,7′-dichlorodihydrofluorescein diacetate (DCFH-DA) as a ROS indicator, under US irradiation (0.5 W cm^−2^, 180 s), ICG induced a 2.41-fold increase in DCF fluorescence, while BT@Lip and BT@Lip-TN exhibited 6.96-fold and 7.08-fold amplifications, respectively (Fig. 2D, E and Fig. S7A), indicating their superior ROS generation capability compared to conventional sonosensitizers. To further identify the specific types of ROS produced by BT@Lip-TN under US irradiation, hydroxyl radicals (•OH), singlet oxygen (^1^O_2_), and superoxide anions (O_2_^•-^) were detected using HPF, ABDA, and DHR123 probes, respectively. The results showed that the •OH probe HPF exhibited a 2.18-fold increase in fluorescence after 180 s of ultrasound irradiation (Fig. 2F–H and Fig. S7B), while ABDA and DHR123 showed no significant changes (Fig. 2G, H and Fig. S8). This suggests that BT@Lip-TN primarily generates •OH under US exposure, with minimal production of ^1^O_2_ and superoxide anions O_2_^•-^. Electron spin resonance (ESR) spectroscopy was used to validate these findings. ^1^O_2_ was detected using 2,2,6,6-tetramethylpiperidine (TEMP) as a spin trap, while •OH were detected using 5,5-dimethyl-1-pyrroline N-oxide (DMPO). The ESR results confirmed that BT@Lip-TN mainly produces •OH under US irradiation, with negligible ^1^O_2_ generation (Fig. 2I and Fig. S9). Notably, while most current sonosensitizers produce type-II ROS (i.e., ^1^O_2_), BT@Lip-TN predominantly generates •OH, which may be attributed to the peroxidation of unsaturated phospholipids within the liposome structure. Furthermore, we investigated the tunability of ROS generation under different concentrations of BT@Lip-TN and varying ultrasound intensities. The results showed that ROS production efficiency was highly dependent on BT@Lip-TN concentration (0-40 μM) under US irradiation at 0.5 W cm^−2^ (Fig. 2J). Additionally, at specific concentration (20 μM), increasing ultrasound intensity enhanced ROS generation (Fig. 2K).

To evaluate whether ultrasound could activate BT@Lip-TN to produce ROS in deep tissues, BT@Lip-TN and ICG solutions were placed in a tissue-mimicking model composed of 1% intralipid emulsion and exposed to 5 min of US irradiation. Compared to ICG, BT@Lip-TN achieved a penetration depth of up to 10 cm (Fig. 2L and Fig. S10), with lower signal attenuation. Finally, the fluorescence penetration depth of BT@Lip-TN in 1% intralipid was assessed, revealing a depth of up to 6 mm (Fig. S11). These results demonstrate that BT@Lip-TN possesses excellent fluorescence and ROS penetration capabilities, highlighting its considerable potential for deep-tissue fluorescence imaging and targeted neuromodulation.

### 2.3 *In vitro* targeted cell uptake, colocalization, and anti-neuroinflammatory mechanism of BT@Lip-TN

**Fig. 3:**
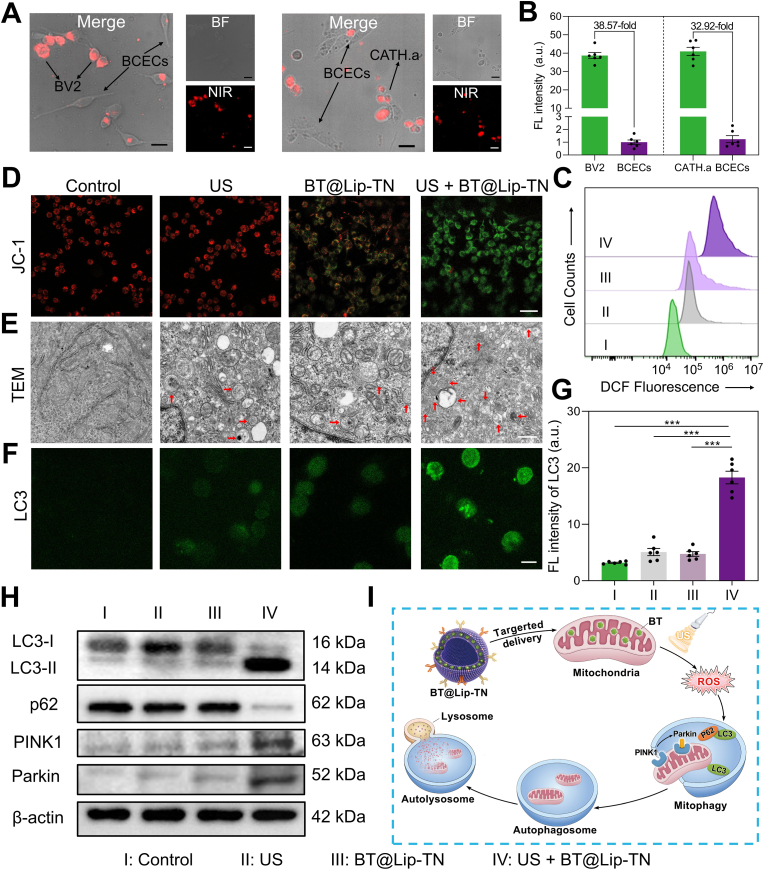
*In vitro* targeting validation and sonodynamic performance of BT@Lip-TN on cellular level. A) Validation of specific uptake of BT@Lip-TN in co-culture systems of BV2/CATH.a and BCECs cells. BF, bright field. Scale bar: 10 μm. B) Quantitative analysis of relative NIR-II fluorescence intensity in BV2, CATH.a, and BCECs cells. C) Flow cytometric analysis of DCFH-DA fluorescence intensity for ROS detection. D) Representative JC-1 fluorescence images of BV2 cells following various treatments. Red channel represents J-aggregates and green channel indicates JC-1 monomer. Scale bar: 20 μm. E) Representative Bio-TEM images showing autophagy levels in BV2 cells post different treatments. Red arrows indicate autolysosomes. Scale bar: 500 nm. F) Confocal images of LC3 immunofluorescence staining in BV2 cells under various treatment conditions. Scale bar: 10 μm. G) Quantitative analysis of the relative fluorescence intensity of LC3. H) Western blot assay of LC3, p62, PINK1, and Parkin in BV2 cells treated with different conditions. I) Schematic diagram illustrating the mechanism of mitophagy-enhanced sonodynamic modulation of BT@Lip-TN. Values are expressed as mean ± S.E.M. (*n* = 6), and analyzed by one-way ANOVA with Tukey's comparisons test. *∗∗∗P* < 0.001.

To validate the cellular selectivity and targeted uptake capability of BT@Lip-TN, we co-cultured BV2 cells (expressing TMEM119), CATH.a cells (expressing NAT), and mouse brain capillary endothelial cells (BCECs). The co-culture system was then incubated with BT@Lip-TN and observed using bright-field (BF) and NIR-II fluorescence microscopy. The results demonstrated that BT@Lip-TN was selectively internalized by BV2 and CATH.a cells (Fig. 3A), with fluorescence intensities 38.57-fold and 32.92-fold higher, respectively, than that in BCECs (Fig. 3B), indicating excellent cellular targeting specificity. Capitalizing on the fluorescence properties of BT@Lip-TN, we further evaluated the time-dependent cellular uptake in BV2 and CATH.a cells. Significant internalization was observed after 2-4 h of incubation, exhibiting robust red fluorescence signals that rapidly peaked within approximately 6-8 h and remained detectable for over 12 h (Fig. S12), highlighting its potential for early neural functional imaging following myocardial I/R injury. To determine the organelle localization of BT@Lip-TN, co-staining with the commercial mitochondrial probe Mito-tracker (green) revealed a Pearson correlation coefficient of 0.897 (Fig. S13), confirming its predominant localization within mitochondria. This phenomenon might be attributed to the electrostatic interaction between the negatively charged mitochondrial structure and the positively charged BT.

Subsequently, cell counting kit-8 (CCK-8) assay was utilized to assess the effect of BT@Lip-TN on cell viability. Under US irradiation (1 MHz, 0.5 W cm^−2^, 50% duty cycle, 5 min), it was observed that BT@Lip-TN concentrations below 40 μM did not induce significant cytotoxicity, whereas that exceeded 40 μM were found to reduce the viability in both BV2 and CATH.a cells (Fig. S14). In line with this, Calcein-AM/PI staining showed that concentrations below 40 μM did not induce significant cell death (Fig. S15). This phenomenon might be ascribed to concentration-dependent ROS generation, thus 40 μM was designated as the maximum safe concentration for subsequent mechanistic exploration. Intracellular ROS production under US activation was verified using the DCFH-DA probe. As shown in Fig. S16, the US + BT@Lip-TN group exhibited stronger green fluorescence compared to the Control, US, and BT@Lip-TN groups. Flow cytometric quantification corroborated these findings (Fig. 3C), confirming that BT@Lip-TN could effectively induce intracellular ROS generation under US irradiation. Furthermore, MitoSOX staining revealed a relatively higher red fluorescence signal in the US + BT@Lip-TN group, reflecting a moderate increase in mitochondrial ROS (Fig. S17).

As a fundamental process in cellular metabolism and quality control, mitophagy selectively eliminates dysfunctional mitochondria and this process is essential for maintaining neuronal homeostasis and mitigating inflammatory responses [[29], [30], [31], [32]]. Enhancing mitophagy has been demonstrated to alleviate neuroinflammation and associated neuronal damage [33,34]. To further explore the effect of BT@Lip-TN-mediated SDT on mitophagy, JC-1 probe was used to detect the changes in mitochondrial membrane potential (MMP). Fluorescence imaging revealed that under US activation, BT@Lip-TN induced the conversion of J-aggregates to JC-1 monomers, indicated by the green fluorescence signal (Fig. 3D), suggesting mitochondrial membrane depolarization. Subsequently, bio-transmission electron microscopy (Bio-TEM), microtubule-associated protein 1 light chain 3 (LC3) immunofluorescence, and monodansylcadaverine (MDC) staining were utilized to evaluate cellular autophagic levels. The results indicated the increased formation of autolysosomes in the US + BT@Lip-TN group (Fig. 3E and Fig. S18). In a similar manner, LC3 immunofluorescence (Fig. 3F, G and Fig. S19) and MDC staining (Figs. S20–S21) in both BV2 and CATH.a cells displayed intense green fluorescence signals in the US + BT@Lip-TN group. In contrast, minimal fluorescence was observed in the Control, US, and BT@Lip-TN groups. Additionally, we employed antibody-conjugated liposomes (Lip-TN) as a control to exclude potential effects of the antibody and liposomal carrier itself on cellular autophagy (Fig. S22). Furthermore, we utilized mannitol to scavenge •OH. LC3 immunofluorescence staining showed that autophagy levels in BV2 cells were reduced upon mannitol treatment, demonstrating the pivotal role of •OH in triggering autophagy (Fig. S22). Collectively, these results indicate that BT@Lip-TN-mediated SDT could significantly enhance autophagic levels in BV2 and CATH.a cells, contributing to the alleviation of sympathetic neuroinflammation following myocardial I/R.

PTEN-induced kinase (PINK1) recognizes depolarized mitochondrial membranes and serves as an initiator of mitophagy, while Parkin amplifies the autophagy signal by ubiquitinating damaged mitochondrial proteins [35,36]. To investigate the signaling pathway through which US + BT@Lip-TN activates mitophagy, the expression levels of PINK1, Parkin, LC3, and p62 were examined by Western blot assay. The results revealed upregulation of Parkin and PINK1, an increased LC3-II/I ratio, and downregulation of the autophagic substrate p62 in the US + BT@Lip-TN group (Fig. 3H and Fig. S23). Collectively, these findings demonstrate that BT@Lip-TN could be selectively taken up by BV2 and CATH.a cells and localize to mitochondria. Under US activation, it induces ROS generation leading to mitochondrial membrane depolarization and promotes mitophagy through the PINK1-Parkin pathway, thus mitigating neuroinflammatory responses (Fig. 3I).

### 2.4 Functional NIR-II fluorescence imaging and pharmacokinetic profiles *in vivo*

**Fig. 4:**
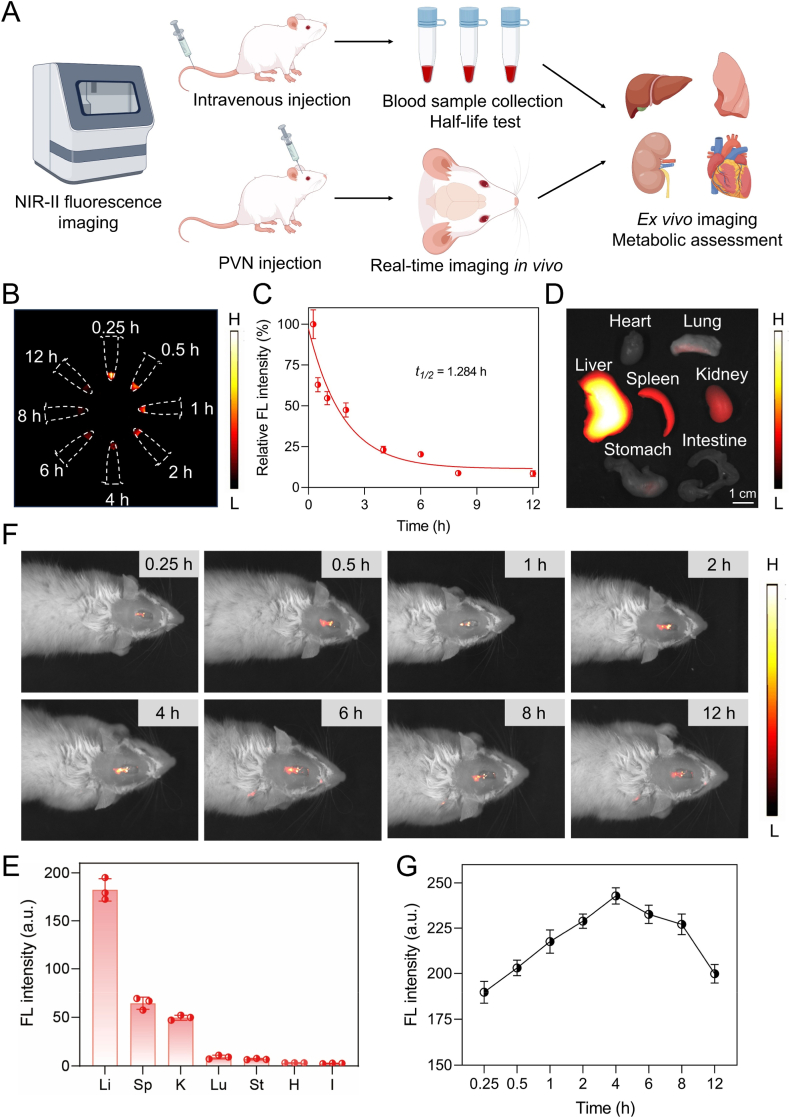
NIR-II fluorescence bioimaging and distribution of BT@Lip-TN *in vivo*. A) Experimental flowchart for evaluating the imaging performance, metabolic and biodistribution characteristics of BT@Lip-TN via intravenous injection (*i.v.*) and PVN microinjection. Created by Figdraw. B, C) Assessment of the blood circulation time of BT@Lip-TN after intravenous administration. D) NIR-II imaging of *ex vivo* organs after intravenous injection of BT@Lip-TN for 12 h and E) quantitative fluorescence intensity analysis of liver (Li), spleen (Sp), kidney (K), lung (Lu), stomach (St), heart (H), and intestine (I). Scale bar: 1 cm. F) *In vivo* real-time NIR-II bioimaging at different time points after microinjection into the PVN and G) time-dependent quantitative analysis of fluorescence intensity. Data are expressed as mean ± S.E.M. (*n* = 3).

During the initial exploratory phase of *in vitro* studies, BT@Lip-TN exhibited outstanding fluorescence characteristics within the NIR-II window. To further evaluate its potential for functional imaging of sympathetic neuroinflammation *in vivo*, we first conducted the hemolysis assay of BT@Lip-TN at various concentrations (Fig. S24). The results showed a hemolysis rate of 3.14% at 200 μM, confirming its favorable biocompatibility. As illustrated in Fig. 4A, we assessed the pharmacokinetic profiles of BT@Lip-TN following both intravenous injection and PVN microinjection. Following intravenous administration of BT@Lip-TN (200 μM, 0.5 mL) in rats, NIR-II fluorescence imaging revealed a blood half-life of approximately 1.284 h (Fig. 4B and C). Subsequent *ex vivo* organ imaging indicated robust fluorescence signals in the liver, spleen, and kidneys, suggesting that BT@Lip-TN might be metabolized through the hepatobiliary system and renal excretion (Fig. 4D and E). Furthermore, real-time imaging following PVN microinjection indicated that fluorescence intensity in the PVN region peaked within approximately 4 h (Fig. 4F and G). To further assess the functional imaging performance of BT@Lip-TN, rat models of myocardial ischemia were established. Subsequent to ischemia induction for 4 h, a significant increase in fluorescence signal was observed within the PVN region (Fig. S25). This phenomenon is primarily attributed to the hyperactivation of sympathetic nerves and microglia in the PVN following myocardial ischemia, leading to enhanced uptake of BT@Lip-TN and consequently intensified fluorescence signals, which reflect the relative level of sympathetic neuroinflammation [37]. Moreover, the rapid peak uptake within 4 h is advantageous for early monitoring of acute conditions such as sympathetic neuroinflammation associated with myocardial I/R injury. This functional imaging is essential for imaging-guided sonodynamic neuromodulation, enabling more precise and individualized modulation strategies. Similarly, *ex vivo* fluorescence signals suggested that after PVN microinjection, BT@Lip-TN might be metabolized through the third ventricle, liver, and kidneys (Fig. S26).

### 2.5 Ultrasound-activated BT@Lip-TN for targeted modulation of PVN to suppress sympathetic neuroinflammation

**Fig. 5:**
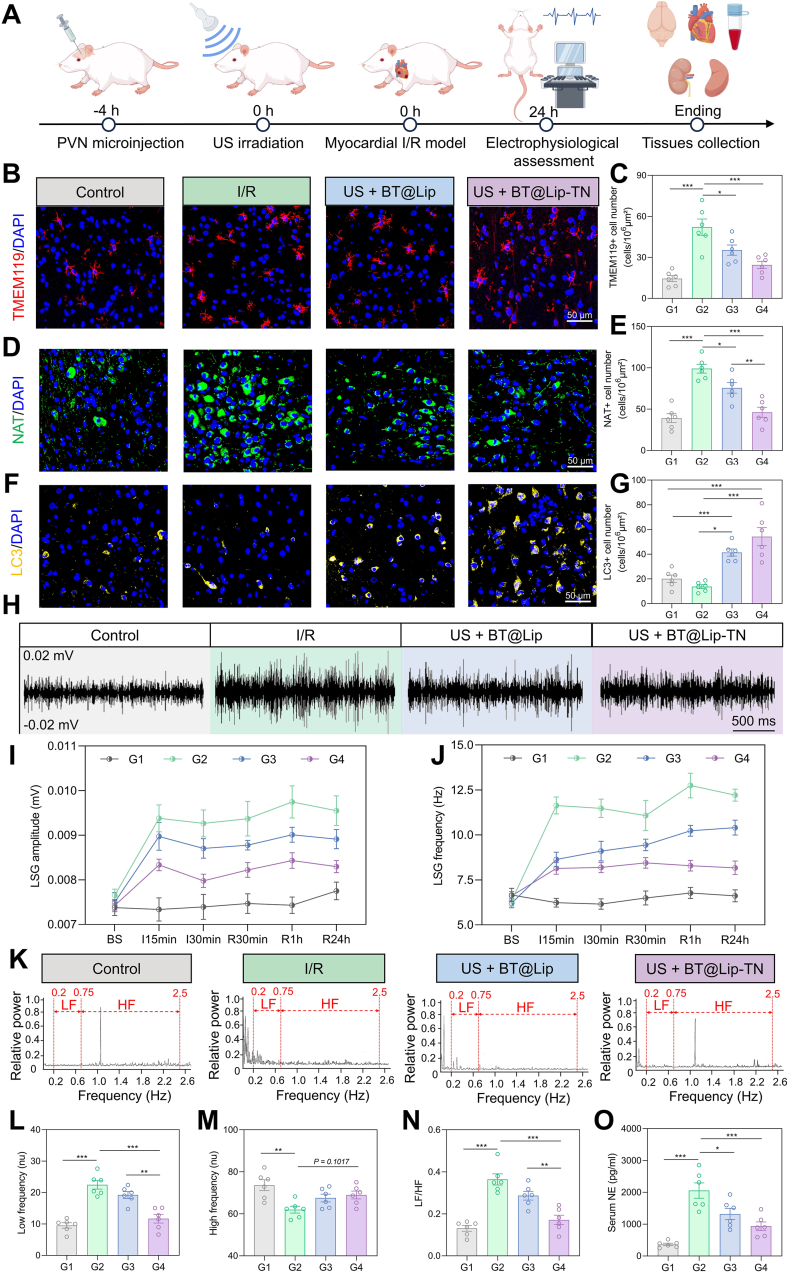
BT@Lip-TN under US activation alleviated sympathetic neuroinflammation. A) The protocols of *in vivo* study. Created by Figdraw. B-G) Representative images of TMEM119 (B, red), NAT (D, green), and LC3 (F, yellow) immunofluorescence staining in the PVN. BT@Lip-TN-mediated sonodynamic neuromodulation significantly suppressed I/R-induced microglial (C) and sympathetic (E) hyperactivation and increased their autophagy levels (G). Scale bar: 50 μm. H) Typical recordings of LSG neural activity in the various groups at the timepoint of reperfusion for 24 h. Scale bar: 500 ms. I, J) Quantification of LSG amplitude and frequency. K) Power spectrum of HRV analysis after various treatment. Statistical analysis of L) low frequency (LF), M) high frequency (HF), and N) the ratio of LF/HF. O) Serum NE levels reflect systematic sympathetic tone. Data are expressed as mean ± S.E.M. (*n* = 6), and analyzed by one-way ANOVA with Tukey's comparisons test. G1, control group; G2, I/R group; G3, US + BT@Lip group; G4, US + BT@Lip-TN group. *∗P* < 0.05, *∗∗P* < 0.01, and *∗∗∗P* < 0.001.

To further investigate the targeted sonodynamic neuromodulatory effects mediated by BT@Lip-TN, we designed *in vivo* experiments as illustrated in Fig. 5A and Fig. S27. Twenty-four Sprague-Dawley rats were randomly assigned to four groups: control group (G1, *n = 6*), I/R group (G2, *n = 6*), US + BT@Lip group (G3, *n = 6*), and US + BT@Lip-TN group (G4, *n = 6*). Based on previously pharmacokinetic results, US irradiation (1 MHz, 1.5 W cm^−2^, 50% duty cycle, 15 min) was applied at 4 h after PVN microinjection, followed by the establishment of myocardial I/R model. Twenty-four hours later, echocardiography, cardiac electrophysiological tests, and tissue collection were performed. Previous studies have demonstrated that the expression of c-fos is rapidly upregulated in cells responding to external stimuli and it is commonly used as an indicator of neuronal activation [38]. Additionally, TMEM119 and ionized calcium binding adapter molecule 1 (Iba-1) are specific biomarkers for microglia, while NAT and tyrosine hydroxylase (TH) are distinct markers of sympathetic neurons [[39], [40], [41], [42]]. Therefore, immunofluorescence staining of the PVN region was employed to evaluate the targeted neuromodulatory effect of US + BT@Lip-TN on neuroinflammation. The results revealed that myocardial I/R significantly increased the proportion of c-fos^+^ neurons in the PVN compared to the control group, which was partially reversed by targeted sonodynamic neuromodulation (Fig. S28). Microglial and sympathetic hyperactivation within the PVN represents a pivotal pathophysiological mechanism that serves to exacerbate myocardial I/R injury [43]. The current study found that both US + BT@Lip and US + BT@Lip-TN significantly reversed the I/R-induced overexpression of TMEM119 (Fig. 5B and C), Iba-1 (Fig. S29), NAT (Fig. 5D and E), and TH (Fig. S30), thereby suppressing sympathetic neuroinflammatory responses. Notably, compared to the US + BT@Lip group, the US + BT@Lip-TN group exhibited superior neuromodulatory effects. These results were mainly attributable to the targeted uptake and accumulation of BT@Lip-TN in sympathetic neurons and microglia, enabling more precise and efficient therapeutic effect. Furthermore, DHE staining and LC3 immunofluorescence staining indicated that US + BT@Lip-TN significantly promote ROS generation (Fig. S31) and enhanced autophagic levels (Fig. 5F and G) within the PVN, consistent with the cellular mechanism of action observed *in vitro*.

The left stellate ganglion (LSG) serves as a critical relay hub through which sympathetic signals from the PVN descend to the heart, and its activity directly reflects cardiac sympathetic tone [44,45]. LSG neural activity was recorded at different time points (Fig. 5H). Analysis of neural signals showed that myocardial I/R injury significantly increased both the discharge amplitude and frequency of the LSG (Fig. 5I and J). Compared to the I/R group and the US + BT@Lip group, the US + BT@Lip-TN group markedly suppressed LSG neural activity (Fig. 5I and J). As a non-invasive quantitative indicator of cardiac autonomic tone, heart rate variability (HRV) parameters include low frequency (LF, reflecting sympathetic activity), high frequency (HF, reflecting parasympathetic activity), and the LF/HF ratio (indicating the relative balance between the two) [46]. HRV was assessed in the different treatment groups and representative power spectra are illustrated in Fig. 5K. Compared to the control group, the I/R group exhibited a significant increase in LF and LF/HF and a decrease in HF, while US + BT@Lip-TN intervention mitigated these changes alterations to a certain extent (Fig. 5L–N). Furthermore, serum levels of the sympathetic neurotransmitter norepinephrine (NE) were elevated in the I/R group but decreased by US + BT@Lip-TN intervention (Fig. 5O). These results indicated that BT@Lip-TN-mediated targeted sonodynamic neuromodulation significantly could alleviate myocardial I/R-induced sympathetic neuroinflammation and cardiac autonomic imbalance.

### 2.6 BT@Lip-TN-mediated targeted sonodynamic neuromodulation alleviated myocardial I/R injury

**Fig. 6:**
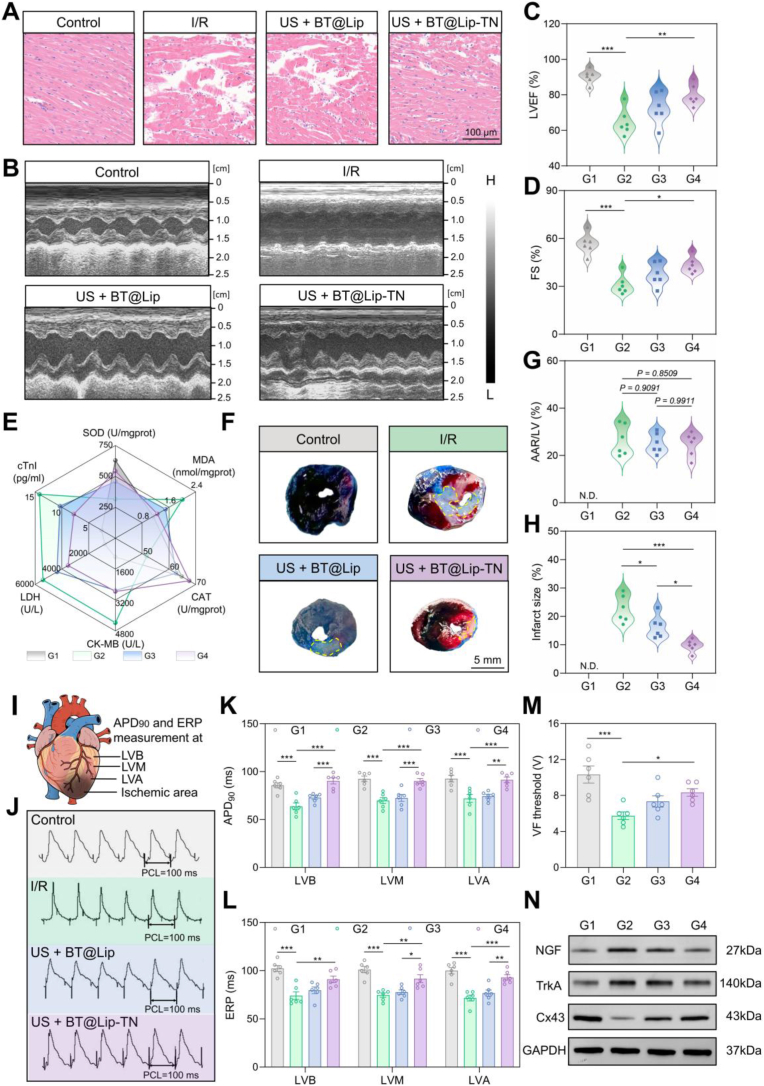
BT@Lip-TN-mediated targeted sonodynamic neuromodulation protected against myocardial I/R injury. A) H&E staining of ischemic myocardium. Scale bar: 100 μm. B) Representative images of M-mode echocardiography in the four groups. BT@Lip-TN under US activation improved C) left ventricular ejection fraction (LVEF) and D) fractional shortening (FS). E) Radar chart for the indicators of myocardial SOD, myocardial MDA, myocardial CAT, serum CK-MB, serum LDH, and serum cTnI. F) Representative images of myocardial Evans blue-TTC staining. Blue, red, and white areas indicate normal perfused myocardium, area at risk (AAR), and infarct zone (dashed yellow line), respectively. Scale bar: 5 mm. G, H) Quantitative analysis of AAR/LV ratio and infarct size. N. D. indicates not detected. I) Schematic diagram of electrode placement for measuring APD_90_ and ERP in the cardiac electrophysiological experiments. J) Representative epicardial monophasic action potential recordings for the measurement of APD_90_ (PCL = 100 ms). K, L) Quantitative analysis of APD_90_ and ERP at different ventricular regions in the four groups. M) Statistical analysis of VF threshold. N) Western blot analysis of NGF, TrkA, and Cx43 expression in peri-ischemic myocardial tissue. Data are presented as mean ± S.E.M. (*n* = 6), and analyzed by one-way ANOVA with Tukey's comparisons test. G1, control group; G2, I/R group; G3, US + BT@Lip group; G4, US + BT@Lip-TN group. *∗P* < 0.05, *∗∗P* < 0.01, and *∗∗∗P* < 0.001.

Myocardial I/R injury encompasses pathophysiological processes including infarct formation, cardiac dysfunction, oxidative stress imbalance, and electrophysiological deterioration, which can be exacerbated by hyperactivated sympathetic nerves and inflammatory responses [47,48]. Therefore, we comprehensively examined various indicators to further explore the cardioprotective effects of BT@Lip-TN-mediated sonodynamic neuromodulation. Hematoxylin and eosin (H&E) staining revealed that US + BT@Lip-TN alleviated the structural disruption of myocardial fibers induced by I/R (Fig. 6A). Furthermore, left ventricular function was assessed using echocardiography (Fig. 6B). Compared with the control group, the I/R group showed significantly reduced left ventricular ejection fraction (LVEF) and fractional shortening (FS). Notably, BT@Lip-TN‒mediated sonodynamic neuromodulation markedly improved left ventricular function, as evidenced by increased LVEF (*P* < 0.01) (Fig. 6C) and FS (*P* < 0.01) (Fig. 6D). We further examined oxidative stress indicators in myocardial tissues and serum levels of cardiac injury biomarkers (Fig. 6E). Encouragingly, US + BT@Lip‒TN intervention reduced the level of oxidative stress indicator malondialdehyde (MDA), while enhancing the activities of the antioxidant enzymes superoxide dismutase (SOD) and catalase (CAT) in the peri-ischemic myocardium (Fig. S32). In addition, serum concentrations of cardiac injury markers, including creatine kinase‒MB (CK-MB), cardiac troponin I (cTnI), and lactate dehydrogenase (LDH), were also reduced following US + BT@Lip-TN treatment (Fig. S33). Myocardial infarct size and area at risk (AAR) were evaluated using Evans blue-triphenyltetrazolium chloride (TTC) staining (Fig. 6F and Fig. S34). Regions stained blue, red, and white indicated normally perfused myocardium, AAR, and infarcted zone, respectively [49]. Quantitative analysis demonstrated that US + BT@Lip-TN did not significantly affect AAR (Fig. 6G) but substantially reduced the myocardial infarct size (US + BT@Lip-TN group: 9.67% ± 0.87% vs. I/R group: 22.58% ± 1.93%; *P* < 0.001) (Fig. 6H).

Additionally, cardiac electrophysiological stability was evaluated by measuring the ventricular action potential duration at 90% repolarization (APD_90_), effective refractory period (ERP), VA inducibility, and ventricular fibrillation (VF) threshold [50]. Fig. 6I illustrates the placement of electrodes for measuring APD_90_ and ERP, including the left ventricular base (LVB), left ventricular middle (LVM), and left ventricular apex (LVA). Compared with the control group, the I/R group exhibited significantly deteriorated electrophysiological stability, manifested as shortened APD_90_ and ERP, increased spatial dispersion (Fig. 6J–L and Figs. S35–S36), enhanced VA inducibility (Fig. S37), and reduced VF threshold (Fig. 6M and Fig. S38). In contrast, the US + BT@Lip-TN group markedly stabilized ventricular electrophysiological properties, prolonging both ERP and APD_90_ while reducing spatial dispersion. Furthermore, targeted sonodynamic neuromodulation decreased VA inducibility and elevated VF threshold. Previous studies have indicated that myocardial ischemia triggers an imbalance in the expression of nerve growth factor (NGF) and its high-affinity receptor tropomyosin receptor kinase A (TrkA), along with downregulation of connexin 43 (Cx43), leading to disrupted local sympathetic innervation and increased heterogeneity in electrical conduction [51,52]. The expression levels of NGF, TrkA, and Cx43 in the peri-ischemic myocardial tissue across different groups were examined using Western blot analysis. The results demonstrated that US + BT@Lip-TN intervention reversed the overexpression of NGF and TrkA and restored Cx43 expression (Fig. 6N and Fig. S39), providing a potential molecular mechanism through which sonodynamic neuromodulation improves cardiac electrophysiological stability.

### 2.7 Transcriptomic analysis to elucidate cardioprotective mechanisms of BT@Lip-TN against myocardial I/R injury

**Fig. 7:**
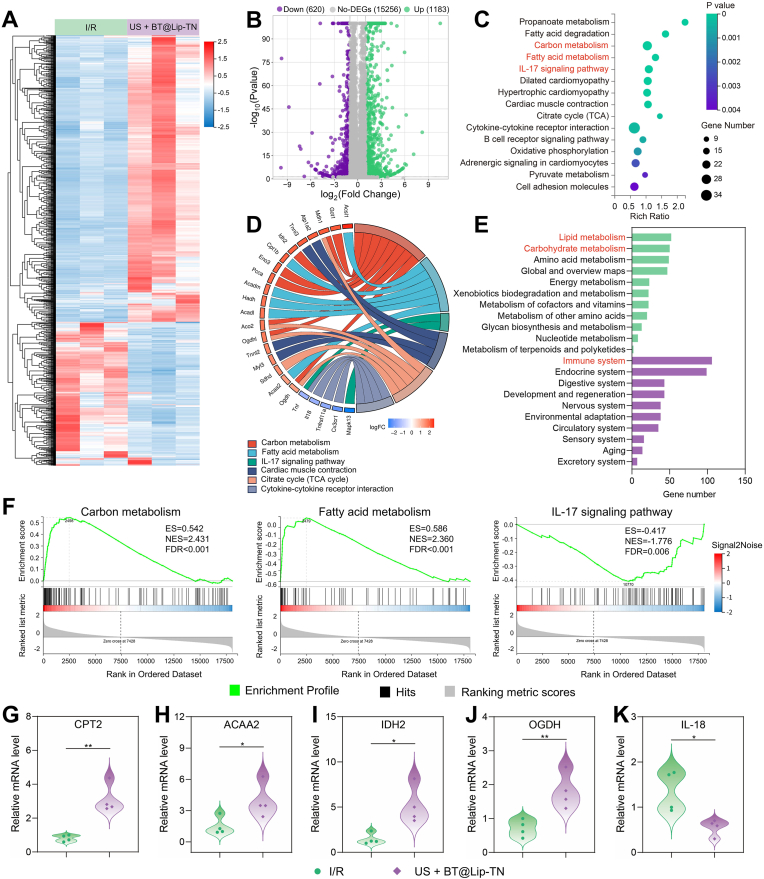
RNA-sequencing analysis of peri-ischemic myocardium. A) Heatmap of differentially expressed genes (DEGs) in the I/R and US + BT@Lip-TN group. B) Volcano plot illustrating up-regulated and down-regulated genes across various treatment groups. C) Bubble plot of KEGG pathway enrichment analysis. D) Chord diagram displaying partial DEGs involved in key immune-metabolic pathways. E) Classification of KEGG enrichment analysis (metabolism and organismal systems) of DEGs. F) GSEA of DEGs. G-K) Relative mRNA levels of CPT2, ACAA2, IDH2, OGDH, and IL-18 (*n = 4*). ∗*P* < 0.05, and ∗∗*P* < 0.01.

In order to further elucidate the underlying molecular mechanisms regarding the targeted sonodynamic neuromodulation of the PVN on myocardial I/R injury, we performed RNA-sequencing of peri-ischemic myocardium from the I/R group, and US + BT@Lip-TN group. Principal component analysis (PCA) delineated significant discrepancies in gene characteristics between the two groups (Fig. S40). A clustering heatmap displays the differentially expressed genes (DEGs) between the two groups (Fig. 7A). The volcano plot of DEGs is shown in Fig. 7B. Compared to the I/R group, US + BT@Lip-TN resulted in the upregulation of 1183 genes and downregulation of 620 genes (Fig. 7B).The bubble plot of Kyoto Encyclopedia of Genes and Genomes (KEGG) enrichment analysis is presented in Fig. 7C, in which we selected immune-metabolic-related pathways including "carbon metabolism", "fatty acid metabolism", and "IL-17 signaling pathway" for further investigation. A chord diagram illustrating the expression of key genes within these pathways is illustrated in Fig. 7D, and the corresponding heatmap can be found in Fig. S41. Furthermore, classification of KEGG pathway revealed that most DEGs were associated with "lipid metabolism", "carbohydrate metabolism", and "immune system" (Fig. 7E and Fig. S42). Subsequently, Gene Set Enrichment Analysis (GSEA) of DEGs related to "carbon metabolism", "fatty acid metabolism", and "IL-17 signaling pathway" is depicted in Fig. 7F. The results indicated that US + BT@Lip-TN primarily up-regulated genes involved in carbon and fatty acid metabolic pathways, while down-regulating genes associated with the IL-17 signaling pathway. Additionally, Gene Ontology (GO) enrichment analysis suggested that the DEGs were predominantly enriched in terms related to "Mitochondria", "Mitochondrial inner membrane" and "Mitochondrial matrix" (Fig. S43). Given that mitochondria are central organelles of energy metabolism and inflammatory responses, we further examined a set of key genes including carnitine palmitoyltransferase 2 (CPT2), acetyl-coenzyme A acyltransferase 2 (ACAA2), isocitrate dehydrogenase 2 (IDH2), oxoglutarate dehydrogenase (OGDH), acyl-CoA dehydrogenase medium chain (ACADM), hydroxyacyl CoA dehydrogenase (HADH), aconitase 2 (ACO2), interleukin-18 (IL-18), tumor necrosis factor-α (TNF-α), and C-X-C motif chemokine receptor 1 (CXCR1) using real-time quantitative polymerase chain reaction (qPCR). The results demonstrated that compared to the I/R group, the US + BT@Lip-TN group exhibited significant up-regulation of CPT2, ACAA2, IDH2, OGDH, ACADM, HADH, ACO2, along with down-regulation of IL-18, TNF-α, and CXCR1 in myocardial tissues (Fig. 7G–K and Fig. S44). These findings suggested that US + BT@Lip-TN intervention could enhance carbon and fatty acid metabolism in cardiomyocytes while suppressing myocardial inflammatory responses, thereby exerting cardioprotective effects (Fig. S45).

Finally, the biocompatibility of BT@Lip-TN is a critical aspect for its potential clinical translation. Major organs (brain, lung, liver, spleen, and kidney) were harvested from rats in each treatment group and subjected to H&E staining. No significant histopathological alterations were observed in any of the groups (Fig. S46). Terminal deoxynucleotidyl transferase mediated dUTP nick-end labeling (TUNEL) staining of the PVN region further confirmed that US + BT@Lip-TN treatment did not induce significant cellular apoptosis (Fig. S47). In addition, blood cell counts and biochemical indicators showed no notable differences among these groups (Figs. S48–S49). Moreover, the temperature of the peri-PVN tissue showed no significant increase following ultrasound intervention, demonstrating that the targeted sonodynamic neuromodulation does not induce thermal damage (Fig. S50). Taken together, these results demonstrate the favorable biocompatibility of BT@Lip-TN and support its potential for future clinical translation.

## 3 Conclusion

In summary, this study constructed an ultrasound-activated, dual-targeted sonosensitizer for neuroinflammation, designated BT@Lip-TN, through antibody-modified liposomes. Our results demonstrate that BT@Lip-TN possesses excellent NIR-II functional imaging properties and exhibits robust ROS generation under ultrasound activation. *In vitro* studies confirmed that BT@Lip-TN is specifically internalized by sympathetic neurons and microglia, localizes to mitochondria, and promotes mitophagy through the PINK1-Parkin pathway under ultrasound irradiation. *In vivo* experiments revealed that BT@Lip-TN enables rapid-feedback real-time fluorescence imaging for monitoring sympathetic neuroinflammation. Furthermore, BT@Lip-TN-mediated targeted sonodynamic neuromodulation suppressed sympathetic neuroinflammation, improved cardiac function, reduced infarct size, and enhanced electrophysiological stability, thereby ameliorating myocardial I/R injury. The current work provides a novel strategy for neuroimmune-targeted drug delivery and precise molecular therapy, while also offering new insights into visualized sonodynamic neuromodulation and clinical interventions for myocardial I/R injury.

## 4 Methods

*Animal ethical statement*: A total of thirty Sprague-Dawley rats (weight: 200-220 g) were included in this study. All experimental procedures were reviewed and approved by the Animal Ethics Committee of Renmin Hospital of Wuhan University (Approval Number: 202300237, 202500258). All protocols complied with the guidelines established by the National Institutes of Health (NIH).

*Statistical analysis*: Data are presented as mean ± standard error of the mean (S.E.M.). Normality and homogeneity of the data were assessed using the Shapiro–Wilk test and F test, respectively. Statistical analysis was performed using unpaired Student's t-test, one-way or two-way analysis of ANOVA followed by Tukey's comparison test using GraphPad Prism 9.0 software. Statistical significance was set at *∗P* < 0.05, *∗∗P* < 0.01, and *∗∗∗P* < 0.001.

## CRediT authorship contribution statement

**Haoyuan Hu:** Conceptualization, Investigation, Methodology, Writing – original draft. **Weiqin Yao:** Conceptualization, Investigation, Methodology, Writing – original draft. **Huijun Wu:** Investigation, Methodology, Writing – original draft. **Qian Li:** Investigation. **Wei Guo:** Investigation. **Yida Pang:** Investigation. **Hong Jiang:** Conceptualization, Investigation, Writing – review & editing. **Yao Sun:** Conceptualization, Funding acquisition, Methodology, Writing – review & editing. **Wei-Hai Chen:** Conceptualization, Funding acquisition, Writing – review & editing. **Songyun Wang:** Conceptualization, Funding acquisition, Methodology, Writing – review & editing.

## Declaration of competing interest

The authors declare that they have no known competing financial interests or personal relationships that could have appeared to influence the work reported in this paper.

## Data Availability

Data will be made available on request.
