# Supplementary material for: Ultrasound-activated dual-targeted liposomes for visualized precise neuromodulation against myocardial ischemia-reperfusion injury
Source: Mater Today Bio. 2026 Feb 24;37:102952. doi: 10.1016/j.mtbio.2026.102952 (PMC12955659; doi:10.1016/j.mtbio.2026.102952)
Supplement: Multimedia component 1 [file mmc1.docx]

Supplementary Materials for

**Ultrasound-activated Dual-targeted Liposomes for Visualized Precise Neuromodulation against Myocardial Ischemia-reperfusion Injury**

Haoyuan Hu^1, 5^, Weiqin Yao^2, 5^, Huijun Wu^1, 5^, Qian Li^3^, Wei Guo^1^, Yida Pang^3^, Hong Jiang^1*^, Yao Sun^4*^, Wei-Hai Chen^2*^, Songyun Wang^1*^

^1^ Cardiovascular Hospital, Renmin Hospital of Wuhan University; Cardiac Autonomic Nervous System Research Center of Wuhan University; Cardiovascular Research Institute, Wuhan University; Hubei Key Laboratory of Cardiology, Wuhan 430060, P.R. China.

^2^ Key Laboratory of Biomedical Polymers of Ministry of Education and Department of Chemistry, Wuhan University, Wuhan, 430072, P. R. China.

^3^ Key Laboratory of Pesticides and Chemical Biology, Ministry of Education, International Joint Research Center for Intelligent Biosensor Technology and Health, College of Chemistry, Central China Normal University, Wuhan 430079, P.R. China.

^4^ College of Biomedicine and Health, Huazhong Agricultural University; Hubei Jiangxia Laboratory, Wuhan 430070, P.R. China.

^5^ These authors contributed equally: Haoyuan Hu, Weiqin Yao, and Huijun Wu.

^*^ Corresponding authors:

Hong Jiang: [hong-jiang@whu.edu.cn](mailto:hong-jiang@whu.edu.cn); Yao Sun: [sunyaogbasp@mail.hzau.edu.cn](mailto:sunyaogbasp@mail.hzau.edu.cn); Wei-Hai Chen: [chenweihai@whu.edu.cn](mailto:chenweihai@whu.edu.cn); Songyun Wang: [songyunwang@whu.edu.cn](mailto:songyunwang@whu.edu.cn).

**Table of contents**

1. Synthetic Procedures………………………………………………………………S3

2. Experimental Section………………………………………………………...........S5

3. Supplementary Figures………………………………………………………...... S13

4. Supplementary Tables…………………………………………………………… S33

5. References………………………………………………………………………. S34

1. **Synthetic Procedures**
   1. **Synthesis of sonosensitizer BT:**

The detailed synthetic route for the sonosensitizer BT is illustrated in Figure S1 according to our previous study.[1] It involves three intermediate compounds (Compounds 2, 3, and 4) and one final product (Compound 5).

***Synthesis of Compound 2*:** Under a nitrogen atmosphere, the Compound 1 (5.0 g, 10.3 mmol, 1.0 equiv.), 4-pyridylboronic acid (2.5 g, 20.6 mmol, 2.0 equiv.), and tetrakis (triphenylphosphine) palladium (0) (594 mg, 0.52 mmol, 0.05 equiv.) were dissolved in 1,4-dioxane (180 mL). Aqueous K_2_CO_3_ solution (0.5 M, 50 mL) was then added. The reaction mixture was heated at 75 °C in an oil bath for 12 h. After completion, the mixture was cooled to room temperature and extracted with dichloromethane and water. The organic phase was collected and dried over anhydrous sodium sulfate. The crude product was purified by silica gel column chromatography (ethyl acetate: dichloromethane = 1:1, V/V) to afford Compound 2 as a yellow solid (1.5 g, 30% yield).

***Synthesis of Compound 3*:** Two clean round-bottom flasks were charged sequentially with Compound 2 (1.4 g, 2.9 mmol, 1.0 equiv.), bis(tributyltin) (1.87 g, 3.2 mmol, 1.1 equiv.), and tetrakis (triphenylphosphine) palladium (0) (167.6 mg, 0.15 mmol, 0.05 equiv.). Under nitrogen protection, toluene (30 mL) was added, and the reaction was carried out under reflux for 12 hours. After cooling to room temperature, the crude product was purified by silica gel column chromatography (petroleum ether: ethyl acetate = 5:1, V/V) to afford Compound 3 as a yellow oily liquid (611.5 mg, 30% yield).

***Synthesis of Compound 4*:** A 50 mL round-bottom flask was charged with Compound 3 (190 mg, 0.28 mmol, 20.0 equiv.), Compound 4 (20.0 mg, 0.056 mmol, 1.0 equiv.), and trans-dichlorobis (triphenylphosphine) palladium (II) (4.0 mg, 0.005 mmol, 0.1 equiv.). The air in the flask was replaced with nitrogen three times. Anhydrous tetrahydrofuran (15.0 mL) was injected, and the mixture was stirred at 100 °C for 48 h. After cooling to room temperature, purification by silica gel column chromatography (dichloromethane: methanol = 100:1) afforded Compound 4 as a green solid (19 mg, 35% yield).

***Synthesis of Compound 5 (BT)*:** Compound 5 (6.5 mg, 0.0065 mmol, 1.0 equiv.) was dissolved in dichloromethane (5 mL), and trifluoromethanesulfonate (4.3 mg, 0.026 mmol, 4.0 equiv.) was added. The reaction was stirred overnight at room temperature. Diethyl ether was then added to the mixture, and the resulting precipitate was collected by filtration to yield the target product BT (5.5 mg, 80% yield).

- 1. **Synthesis of targeted liposomes** **BT@Lip-TN**

The design and synthesis process of BT@Lip-TN is depicted in Figure 1A. BT@Lip was synthesized by one-pot process. Briefly, lecithin (30 mg), cholesterol (15 mg) and DSPE-PEG_2000_-MAL (5 mg) were dissolved in 20 mL chloroform and mixed sufficiently by ultrasonic apparatus. Then BT of different quality (1 mg, 2 mg, 5 mg and 10 mg) was dissolved into 10 mL methanol respectively and added dropwise into the liposome mixture solution. After stirring for 2 h, rotary evaporator was used to remove the chloroform and methanol. The product was dried at 40 ℃ for 24 h in the vacuum oven, and then hydrated in 20 mL glucose solution (glucose concentration = 5%) at 40 ℃ for 4 h. Finally, the liposome solution was extruded through the liposome extruder (aperture of filter membrane = 200 nm) and dialyzed by ultrapure water for 24 h in order to remove the extra BT. After the dialysis solution was freeze-dried, BT@Lip was totally prepared. Subsequently, the BT@Lip, TMEM119 conjugated to Alexa Fluor® 647 (ab313674, Abcam, UK) and NAT (A1421, Abclonal, China) conjugated to Alexa Fluor® 488 (ab236553, Abcam, UK) were uniformly dispersed in ultra-pure water and cocultured for 2 h to attach the antibodies to the surface of the BT@Lip. Thereafter, the mixed solution was centrifuged at 12000 rpm for 5 min and the precipitate was collected, followed by triple washing (12000 rpm, 5 min) with ultrapure water to remove excess antibodies, thereby yielding BT@Lip-TN.

**2. Experimental Section**

**2.1 Characterization analysis of BT@Lip and BT@Lip-TN**

***Characterization of analysis of*** ***BT@Lip and BT@Lip-TN*:** The hydrodynamic diameter and Zeta potential of BT@Lip and BT@Lip-TN nanoparticles in ultrapure water at pH 7.4 were measured by the Malvern Zetasizer ZEN3600. The morphology of BT@Lip and BT@Lip-TN were represented via the transmission electron microscope (TEM, JEM-2100). The successful decoration of Alexa Fluor® 647-TMEM119 mab (λ_ex_ = 652 nm, λ_em_ = 668 nm) and Alexa Fluor® 488-NAT mab (λ_ex_ = 495 nm, λ_em_ = 519 nm) on BT@Lip was validated by detecting the colocalized fluorescent signals on the surface of BT@Lip via super-resolution microscopy.

***Loading ratio and encapsulation efficiency of BT in*** ***BT@Lip*:** 1mg BT@Lip prepared with different feeding ratios (M_Liposomes_: M_BT_ = 50:1, 50:2, 50:5, 50:10) was dissolved in 2 mL dimethyl sulfoxide (DMSO) for 4 h to destroy the liposomes and release BT within them. Then the mixed solutions were centrifuged at 12000 rpm for 5 min to collect the supernatant. Subsequently, the supernatant was tested by Ultraviolet Spectrophotometer (UV-Vis) for evaluating the contents of remaining BT. The drug loading ratio and encapsulation efficiency of BT in BT@Lip were calculated through the computational formula: encapsulation efficiency = $m_{1}/m_{2}$, loading ratio = $m_{1}/m_{3}$, where $m_{1}$ represents the mass of BT loaded in BT@Lip, $m_{2}$ stands for the mass of BT that was initially used to synthesize BT@Lip, $m_{3}$ represents the initial mass of BT@Lip.

***Drug release capability of BT@Lip*:** 2 mL BT@Lip (1 mg/mL) was filled into dialysis bag (MWCO 5000) and placed in 5 mL PBS (pH = 6.5 and pH = 7.4) with uninterrupted stirring at 37 ℃. 5 mL dialysate was taken out for examine and the same volume of fresh PBS was refilled at 0.5 h, 1 h, 2 h, 4 h, 6 h, 8 h, 12 h, 24 h, 48 h, 72 h, 96 h. The cumulative amount of BT released from BT@Lip-TN in PBS (pH = 6.5 and pH = 7.4) at different time points was measured by Ultraviolet Spectrophotometer. The cumulative quantity of BT released from BT@Lip could be calculated through the computational formula: $m_{t}= C_{t}V+V\sum_{0}^{t-1} C_{t}$, where $m_{t}$ represents the mass of BT released from BT@Lip at time t, $C_{t}$ is the concentration of BT in the collected solution at time t, $V$ is the collected sample volume (5 mL).

***Sodium dodecyl sulfate-polyacrylamide gel electrophoresis (SDS-PAGE)*:** To validate antibody conjugation to liposomes, BT@Lip and BT@Lip-TN were dissolved in DMSO and subjected to reducing SDS-PAGE (10% gel). The gel was then placed in a glass dish, stained with Coomassie Brilliant Blue solution (G2021, Servicebio, China) at 70 °C for 5 min, and incubated on a shaker for 90 min. After washing, the gel was treated with destaining solution (G2022, Servicebio, China) and destained overnight on a shaking platform. Finally, the gel was rinsed with water and imaged.[2]

***Fluorescence emission and UV-Vis spectroscopy*:** Fluorescence emission spectra of BT@Lip and BT@Lip-TN (20 μM) were acquired using a FluoroMax+ spectrophotometer with an excitation wavelength of 808 nm and an emission range of 820-1500 nm. UV-Vis absorption spectra were recorded on a UV-1900i spectrophotometer over a wavelength range of 300-900 nm.

***Stability assessment*:** BT@Lip-TN was incubated in PBS and 10% FBS for one week. The hydrodynamic diameter was measured daily to evaluate its chemical stability. To assess sonostability, samples were exposed to ultrasound irradiation (1 MHz, 0.2-1.5 W cm^-2^), and UV-Vis absorption and fluorescence were recorded at five-minute intervals. Similarly, photostability was evaluated by monitoring UV-Vis spectra following laser irradiation (808 nm, 0.33 W cm^-2^).

***Reactive oxygen species (ROS) generation detection in solution*:** The ROS indicator DCFH-DA (D6683, Sigma-Aldrich, UK) was used to evaluate total ROS generation by ICG, BT@Lip, and BT@Lip-TN under ultrasound activation. DCFH-DA (10 mM, 50 μL) was mixed with 450 μL DMSO, followed by addition of NaOH (10 mM, 0.5 μL). After incubation in the dark for 30 min, DCFH was obtained by hydrolysis. The activated DCFH solution (10 mM, 0.1 μL) was added to aqueous solutions of ICG, BT@Lip, and BT@Lip-TN, which were then irradiated with ultrasound for varying durations. Fluorescence spectra of DCF (λ_ex_ = 488 nm, λ_em_ = 525 nm) induced by ICG, BT@Lip, and BT@Lip-TN were recorded. Hydroxyl radicals (•OH) and superoxide anions (O_2_^•⁻^) were detected using HPF (H4290, Sigma-Aldrich, UK) and DHR123 (D23806, Thermo Fisher Scientific, USA), respectively. Stock solutions (5 mM) of HPF and DHR123 were diluted to 5 μM working solutions. BT@Lip-TN (20 μM) was added to 3 mL of working solution and stimulated with US (1 MHz, 0.5 W cm⁻², 50% duty cycle). The generation of •OH and O_2_^•⁻^ was quantified by changes in fluorescence spectra. Singlet oxygen (^1^O_2_) was detected using ABDA (75068, Sigma-Aldrich, UK). The ABDA working solution (1 mg/mL) was prepared in DMSO. Then, 20 μL of ABDA working solution was added to 2 mL of BT@Lip-TN, and changes in UV absorption after US irradiation were measured to evaluate ^1^O_2_ generation. Additionally, the spin trap 2,2,6,6-tetramethylpiperidine (TEMP) (Y100068, Beyotime, China) was used for ^1^O_2_ detection, while 5,5-dimethyl-1-pyrroline N-oxide (DMPO) (Y157934, Beyotime, China) was employed for detecting •OH and O_2_^•⁻^ via ESR spectroscopy.

***Assessment of fluorescence and ROS penetration depth*:** To evaluate the tissue penetration depth of fluorescence, 1% intralipid emulsion was used to simulate biological tissue. Glass capillaries filled with ICG (40 μM) or BT@Lip-TN (40 μM) were fixed at the bottom of a cylindrical dish. The dish was filled with varying volumes of intralipid emulsion, and the depth of the capillary was calculated based on the cross-sectional area of the dish. Fluorescence images were acquired under 808 nm laser excitation. To further assess the penetration depth of ROS in simulated biological tissue, 1% intralipid emulsion solidified with 1% agarose was used (depth range: 0-10 cm). Test tubes containing ICG (40 μM) or BT@Lip-TN (40 μM) and DCFH (10 μM) were subjected to ultrasound irradiation for 5 min at different heights. The solutions were then transferred to a 96-well plate for fluorescence imaging. Image analysis was performed using Image J software.

**2.2 *In vitro* experimental methods**

***Cell culture*:** BV2 cells (from mice microglial cell line) were cultured in MEM medium supplemented with 10% fetal bovine serum (FBS), 1% penicillin, 1% streptomycin, and 1% amphotericin B. CATH.a cells (from mice neuronal cell line) were maintained in RPMI-1640 medium containing 8% horse serum, 4% FBS, 1% penicillin, and 1% streptomycin. BCECs (from mice brain capillary endothelial cells) were cultured in DMEM medium with 10% FBS, 1% streptomycin, and 1% amphotericin B. All cells were cultured at 37 °C in a humidified incubator with 5% CO_2_.

***Cellular uptake assay and targeting evaluation by NIR-II fluorescence imaging*:** BV2 and CATH.a cells were seeded separately in 35-millimetre Petri dishes (approximately 2 × 10^6^ cells per dish). After incubating with BT@Lip-TN (40 µM) for different durations (0-12 h), cells were washed three times and imaged using an NIR-II fluorescence microscope (λ_ex_ = 808 nm, λ_em_ = 1000-1200 nm). To evaluate the cellular targeting specificity of BT@Lip-TN, co-culture systems of BV2/BCECs and CATH.a/BCECs were established. The co-cultures were incubated with BT@Lip-TN (40 µM) for 12 h, followed by observation using NIR-II fluorescence and bright-field (BF) microscopy to assess fluorescence distribution and cellular morphology, thereby determining the uptake levels of BT@Lip-TN across different cell types.

***Cell viability assessment*:** The Cell Counting Kit-8 (CCK-8) assay was employed to evaluate the effects of BT@Lip-TN at varying concentrations on the viability of BV2 and CATH.a cells under ultrasound activation. Cells (approximately 5 × 10^3^ per well) were seeded into 96-well plates and incubated for 24 h. Subsequently, different concentrations (10 μM, 20 μM, 40 μM, 80 μM, and 160 μM) of BT@Lip-TN were added, followed by incubation in the dark at room temperature for 4 h. Afterward, the cells were subjected to either ultrasound or sham ultrasound intervention and further incubated for 12 h. Then, 10 μL of CCK-8 solution was added to each well. After 1 h, the optical density at 450 nm (OD_450_) was measured using a microplate reader, and relative cell viability was calculated.

***Calcein-AM/PI Staining:*** Cell viability was assessed using a Calcein-AM/PI Live/Dead Cell Staining Kit (C2015, Beyotime, China). Briefly, BV2 cells were seeded in 12-well plates and cultured for 24 h. The cells were then treated with the compound at concentrations of 0, 10, 20, 40, 80, and 160 mM for 4 h, followed by ultrasound treatment for the designated groups. Eight hours post-treatment, the culture medium was removed, and the cells were washed twice with PBS. Next, 500 μL of freshly prepared Calcein-AM/PI working solution was added to each well, and the cells were incubated at 37°C in the dark for 30 mi. After incubation, images were observed and captured using a fluorescence microscope (Calcein-AM: λ_ex_ =494 nm, λ_em_ =517 nm; PI: λ_ex_ = 535 nm, λ_em_ = 617 nm).

***Mitochondrial colocalization assay*:** BV2 cells were seeded in confocal dishes at a density of 1 × 10^4^ cells per dish and cultured overnight. After incubation with BT@Lip-TN (40 μM) for 4 h, the cells were stained with Mito-Tracker^®^ Green (50 nM) (C1048, Beyotime, China) for 45 min. Subsequently, the cells were washed three times with PBS. Fluorescence images were acquired using the NIR-II fluorescence microscope. For the Mito-Tracker^®^ Green channel, the excitation wavelength was set to 490 nm and emission was collected at 516 nm. Image analysis was performed using Image J software.

***Mitochondrial membrane potential (MMP) detection:*** The 5,5′,6,6′-Tetrachloro-1,1′,3,3′-tetraethyl-imidacarbocyanine iodide (JC-1) probe was used to detect mitochondrial membrane potential. BV2 cells were seeded in 12-well plates at a density of 5 × 10⁴ cells/mL and cultured overnight. Cells were treated with PBS, US (1.0 MHz, 0.5 W cm⁻², 50% duty cycle, 5 min), BT@Lip-TN (40 μM), and US + BT@Lip-TN (40 μM), respectively. Subsequently, cells were incubated with JC-1 (2 μg/mL) (C2006, Beyotime, China) and 1% DMSO in MEM medium at 37 °C in the dark for 30 min. Fluorescence images were captured using an upright microscope. For JC-1 monomers, the excitation wavelength was set at 514 nm, and the emission filter was adjusted to approximately 529 nm. For JC-1 aggregates, excitation at 585 nm was applied, and emission was collected at 590 nm.

***Intracellular ROS detection*:** The DCFH-DA assay was used to detect intracellular ROS levels. BV2 cells were incubated with BT@Lip-TN (40 μM) for 4 h, and then exposed to ultrasound irradiation (1.0 MHz, 0.5 W cm^-2^, 50% duty cycle, 5 min). The DCFH-DA fluorescent probe (20 μM) (S0033S, Beyotime, China) was subsequently added and incubated for 30 min in the dark conditions. After washing three times with PBS, fluorescence imaging was performed using an inverted fluorescence microscope. For flow cytometry analysis, DCFH-DA was used as the probe, and the ROS levels in BV2 cells under after treatments were assessed using a CytoFLEX flow cytometer. The data were processed using FlowJo VX software (BD Biosciences, USA).

***MitoSOX Red staining:*** Measurement of mitochondrial superoxide was performed using the MitoSOX™ Red fluorescent probe (M36008, Thermo Fisher, USA). BV2 cells were seeded in confocal dishes and cultured for 24 h, followed by treatment with BT@Lip-TN (40 μM) for 4 h. After treatment, the culture medium was removed, and the cells were gently washed with PBS. The MitoSOX Red working solution (500 nM) was prepared and added to each dish to completely cover the cells, followed by incubation at 37°C in the dark for 30 min. After incubation, the cells were gently washed three times with PBS to remove excess probe. Finally, images were observed and acquired using a confocal microscope (λ_ex_ = 396 nm, λ_em_ = 610 nm).

***Bio-transmission electron microscopy (Bio-TEM):*** BV2 cells were divided into four groups: Control, US, BT@Lip-TN, and US + BT@Lip-TN. After various treatments, the culture medium was removed and cells were fixed with 2.5% glutaraldehyde for 15 min. Subsequently, cells were collected using a cell scraper, and further fixed with 1% osmium tetroxide for 12 h. After washing with PBS, the samples were dehydrated using a graded ethanol series. The dehydrated cells were embedded in a mixture with embedding agent (SPI, 90529-77-4), sectioned into ultrathin slices, stained with 2% lead acetate, and ultimately observed by a transmission electron microscope (HT7800, HITACHI, Japan).

***LC3 immunofluorescence staining and MDC staining*:** BV2 and CATH.a cells were seeded in confocal dishes and cultured overnight. After different treatment, cells were washed three times with PBS, permeabilized with 0.1% Triton X-100 in PBS for 5 min, and fixed in fixative solution for 30 min. Subsequently, the cells were incubated with an anti-LC3B antibody (A19665, Abclonal, China) at 4 °C overnight. After PBS washes, cells were treated with a CoraLite488-conjugated secondary antibody (λ_ex_ = 488 nm, λ_em_ = 515 nm) for 1 h and then analyzed using a confocal microscope. MDC staining was performed according to the manufacturers’ instructions. For confocal imaging, the excitation wavelength was set to 405 nm and emission wavelength was set between 510-540 nm.

***Western blot analysis*:** Proteins were extracted from BV2 cells and peri-ischemic myocardium for Western blot analysis. After separation by SDS-PAGE, the proteins were transferred to a PVDF membrane and blocked with 5% bovine serum albumin (BSA) (GC305010, Servicebio, China) for 30 min. The membranes were washed three times with TBST and then incubated overnight at 4 °C with primary antibodies anti-LC3B (A19665, Abclonal, China), anti-p62 (A19700, Abclonal, China), anti-PINK1 (A7371, Abclonal, China), anti-Parkin (A0968, Abclonal, China), anti-NGF (GB111206, Servicebio, China), anti-TrkA (83477-7-RR, Proteintech, China), anti-Cx43 (GB12234, Servicebio, China), and anti-GAPDH (A19056, Abclonal, China). Subsequently, the membranes were incubated with corresponding secondary antibodies at room temperature for 2 h. After washing with TBST, images were acquired using a chemiluminescence imaging system (Bio-Rad, USA). Semi-quantitation of the blot images was performed using Image J software.

**2.3 *In vivo* experimental protocol**

***Animal preparation:*** All rats used in this study were supplied by the Animal Experiment Center of Renmin Hospital of Wuhan University. A total of thirty male Sprague-Dawley rats (6-8 weeks, weighing 200-220 g) were used in this study. Among them, six rats were utilized for *in vivo* NIR-II imaging, and the remaining rats were randomly divided into four groups: Control, I/R, US + BT@Lip, and US + BT@Lip-TN for the assessment of therapeutic efficacy and biocompatibility. Inhalation anesthesia was induced with 3% isoflurane and maintained with 1.5% isoflurane. During anesthesia, oxygen was continuously administered to maintain adequate oxygenation, and a heating pad was used to sustain a stable body temperature. All experimental procedures were reviewed and approved by the Animal Ethics Committee of Renmin Hospital of Wuhan University (Approval No.: 202300237, 202500258) and conducted in accordance with the guidelines established by the National Institutes of Health (NIH).

***PVN microinjection and ultrasound intervention*:** According to the rat brain atlas, the PVN was identified at 1.8 mm posterior to the bregma, 0.3 mm lateral to the midline, and 7.9 mm below the skull surface. [1] PBS, BT@Lip, or BT@Lip-TN (200 μM) was microinjected bilaterally into the PVN (5 μL per side) using a stereotaxic instrument and a microsyringe. The optimal time window for ultrasound irradiation was determined based on prior pharmacokinetic results. Ultrasound (UT1021, Nu-Tek, China) parameters utilized for *in vivo* experiments are detailed in Table S2.

***Hemolysis assay:*** To evaluate the hemocompatibility of BT@Lip-TN, fresh rat blood was collected in heparinized tubes and washed three times with PBS to obtain the red blood cell (RBC) suspension. Different concentrations of BT@Lip-TN (dissolved in PBS) were adjusted to a final volume of 0.5 mL, mixed with 0.5 mL of the RBC suspension, and incubated in the 37 °C water bath for 60 min. After centrifugation at 3000 rpm for 10 min, the supernatant was carefully collected. Photographs were taken, and the optical density (OD) of the supernatant was measured at 540 nm using a 96-well plate. Saline and deionized water were used as negative and positive controls, respectively. The hemolysis ratio was calculated using the following formula: [3]

$$Hemolysis R\mathrm{ate} \left( \% \right)= \frac{{OD}_{sample}-{OD}_{saline}}{{OD}_{water}-{OD}_{saline}} \times100\%$$

***In vivo fluorescence imaging:*** After intravenous injection of BT@Lip-TN (200 μM, 0.5 mL) via the tail vein, blood samples were collected at predetermined time points (0.25, 0.5, 1, 2, 4, 6, 8, and 12 h). Fluorescence images were acquired using an NIR-II fluorescence imaging system (Suzhou NIR-Optics, China), and fluorescence intensity was quantified with Image J software. The fluorescence intensity-time curve was plotted to calculate the blood half-life of BT@Lip-TN. For localized imaging, BT@Lip-TN (200 μM, 5 μL) was microinjected into the bilateral PVN, and continuous imaging was performed using the same NIR-II system. After imaging, major organs were harvested for *ex vivo* imaging.

***Establishment of myocardial I/R model*:** Following anesthesia, rats were intubated tracheally and mechanically ventilated to maintain oxygenation. A left thoracotomy was performed through the 3rd-4th intercostal space. The left anterior descending coronary artery was ligated approximately 5 mm below the left auricle using a 6-0 silk suture. Myocardial ischemia was confirmed by ST-segment elevation on the electrocardiogram and regional pallor of the myocardial tissue. After 30 min of ischemia, the suture was released to allow reperfusion. The chest was then closed in layers, and a heating pad was used to maintain the rats’ body temperature throughout the procedure.[4]

***Echocardiography*:** Twenty-four hours after establishing the myocardial I/R model, echocardiography was performed to assess left ventricular function. After anesthesia, transthoracic echocardiographic parameters were acquired using a high-resolution imaging system (Vivid E95, GE, USA) and analyzed. The echocardiographic parameters mainly included left ventricular ejection fraction (LVEF) and fractional shortening (FS). All operations and data analysis were conducted by a sonographer blinded to the group assignments.

***LSG neural activity recording*:** After anesthesia, the left stellate ganglion (LSG) was carefully dissected and isolated. Neural activity was recorded using a platinum-coated recording microelectrode and a PowerLab data acquisition system (8/35, AD Instruments, Australia). One-minute segments of neural activity data were analyzed using LabChart 8.0 software. LSG neural activity was defined as deviations with a signal-to-noise ratio exceeding 3:1, consistent with our previous methodological approach.[5]

***Myocardial Evans blue-TTC staining*:** At the end of the experiment, 2% Evans blue dye (T-1824, MCE, USA) was injected via the inferior vena cava and allowed to circulate and distribute uniformly in non-occluded areas. Following euthanasia, the heart was excised and rinsed with PBS. The heart was frozen at -20 °C for 20 min and then transversely sectioned into five 1-mm-thick slices. The slices were incubated in 1% triphenyltetrazolium chloride (TTC) (G1017, Servicebio, China) solution at 37 °C for 30 min. After washing with PBS, the slices were fixed in 4% paraformaldehyde for 24 h. Images of myocardial sections were captured using a digital camera and analyzed with Image J software. Blue-stained areas indicated normally perfused myocardium; red areas represented the area at risk (AAR); and white areas denoted infarcted tissue. [6]

***Heart rate variability (HRV)*:** Electrocardiograms (ECG) were recorded using a PowerLab data acquisition system. HRV was analyzed from 5-minute stable ECG segments using LabChart 8.0 software. Parameters included low frequency (LF, 0.25-0.75 Hz), high frequency (HF, 0.75-2.5 Hz), and the LF/HF ratio.[7]

***Biochemistry tests and enzyme-linked immunosorbent assay (ELISA)*:** Serum and peri-ischemic myocardial tissue samples were used for biochemical and ELISA. To evaluate myocardial oxidative stress, malondialdehyde (MDA), catalase (CAT), and superoxide dismutase (SOD) levels were measured using commercial biochemical assay kits (G4302, Servicebio, China) (G4307, Servicebio, China) (A001-1, JianCheng Bioengineering Institute, China). Serum levels of lactate dehydrogenase (LDH) (G1610, Servicebio, China) and creatine kinase isoenzyme (CK-MB) (GM1122, Servicebio, China) were determined according to the manufacturers’ instructions. Serum cardiac troponin I (cTnI) (SEA478Ra, Cloud-Clone Crop., China) and norepinephrine (NE) (ELK8956, ELK Biotechnology, China) concentration were assessed using the corresponding ELISA kits.

***Cardiac electrophysiological experiments*:** Cardiac electrophysiological parameters were recorded and analyzed using a four-channel electrode system, the PowerLab data acquisition system, and LabChart 8.0 software. Action potential duration at 90% repolarization (APD_90_) and effective refractory period (ERP) were measured at three sites: the left ventricular base (LVB), left ventricular middle (LVM), and left ventricular apex (LVA) (Figure 6I). Continuous pacing was performed at a pacing cycle length (PCL) of 100 ms (Figure 6J). Monophasic action potential (MAP) signals were analyzed using PowerLab 8.0 software to calculate APD_90_. ERP was determined using programmed electrical stimulation consisting of eight consecutive S1-S1 stimuli (cycle length =140 ms) followed by a decremental S2 stimulus. The S1-S2 interval was initially set at 120 ms and decreased in 10-ms steps until approaching the ERP, where the step size was reduced to 2 ms. The ERP was defined as the longest S1-S2 interval that failed to capture the ventricle (Figure S36). Spatial dispersion of APD_90_ and ERP was calculated as the coefficient of variation [(standard deviation / mean) × 100%] across the three recording sites using the following formula:[8, 9]

$${APD}_{90} dispersion= \frac{Standard deviation of {APD}_{90}}{Mean of {APD}_{90}}\times100\%$$

$$ERP dispersion= \frac{Standard deviation of ERP}{Mean of ERP}\times100\%$$

To assess ventricular arrhythmia (VA) inducibility, programmed electrical stimulation was performed using eight continuous stimuli (S1) followed by 1-3 extra stimuli (S2, S3, S4) at shorter coupling intervals (Figure S37). Arrhythmia score (ranging from 0 to 8) was assigned based on the number of extra stimuli and the occurrence of VA, following a detailed protocol consistent with our previous study.[10] The ventricular fibrillation (VF) threshold was measured via a 2-second burst pacing train starting at 4.0 V, with increments of 0.5 V. The VF threshold was defined as the minimum voltage required to induce VF (Figure S38).

***Histopathological staining*:** Brain and heart tissues were harvested and fixed in 4% paraformaldehyde or stored at -80 °C for further analysis. Brain sections containing the PVN were subjected to immunofluorescence staining for c-fos (GB11069, Servicebio, China), TMEM119 (ab313674, Abcam, UK), NAT (A1421, Abclonal, China), Iba-1 (ab178846, Abcam, UK), TH (ab137869, Abcam, UK), and LC3 (A19665, Abclonal, China) to evaluate the effects of US + BT@Lip-TN on sympathetic neurons and microglia within the PVN. Intracellular ROS levels in the PVN were detected using dihydroethidium (DHE) staining. Cardiac tissue sections were stained with hematoxylin and eosin (H&E) to assess morphological changes in the myocardium in various groups.

***Transcriptome analysis*:** After harvesting peri-ischemic myocardial tissue, it was rapidly frozen in liquid nitrogen, and samples were stored at -80°C for RNA-sequencing. Gene sequencing was conducted and raw reads were obtained by the BGSEQ-500 platform (BGI-Shenzhen, China). Subsequently, hierarchical indexing was adopted to map to the genome for transcript splice alignment. The differentially expressed genes (DEGs) were defined as "Fold Change ≥ 2 and Adjusted *P* ≤ 0.05".[11] R software was used for cluster analysis of DEGs and KEGG pathway analysis among different groups.

***Quantitative real-time quantitative polymerase chain reaction (qPCR)*:** To measure mRNA relative levels, RNA was extracted from left ventricular tissue using Trizol reagent (G3013, Servicebio, China). Reverse transcription was performed using the RevertAid First Strand cDNA Synthesis Kit (K1622, Thermo, USA), followed by real-time qPCR quantification analysis with a fluorescence qPCR instrument (CFX Connect, Bio-rad, USA). mRNA relative levels were calculated according to the 2^–ΔΔCt^ method, normalized to GAPDH. The primer sequences for the relevant genes were listed in Table S3.

***Biocompatibility assessment*:** At the end of the experiment, major organs (including the brain, lung, liver, spleen, and kidney) were collected and subjected to H&E staining to evaluate potential histopathological damage across treatment groups. Coronal sections containing the PVN were processed for terminal deoxynucleotidyl transferase mediated dUTP nick-end labeling (TUNEL) staining to assess the effect of US + BT@Lip-TN on cellular apoptosis. Blood samples were used to conduct blood cell count and biochemical tests to examine the influence of BT@Lip-TN on hematological parameters and hepatic/renal function. The temperature changes of the rat scalp before and after ultrasound intervention were measured using a thermal imaging camera (H23, HIKMICRO, Germany).

**3. Supplementary Figures**


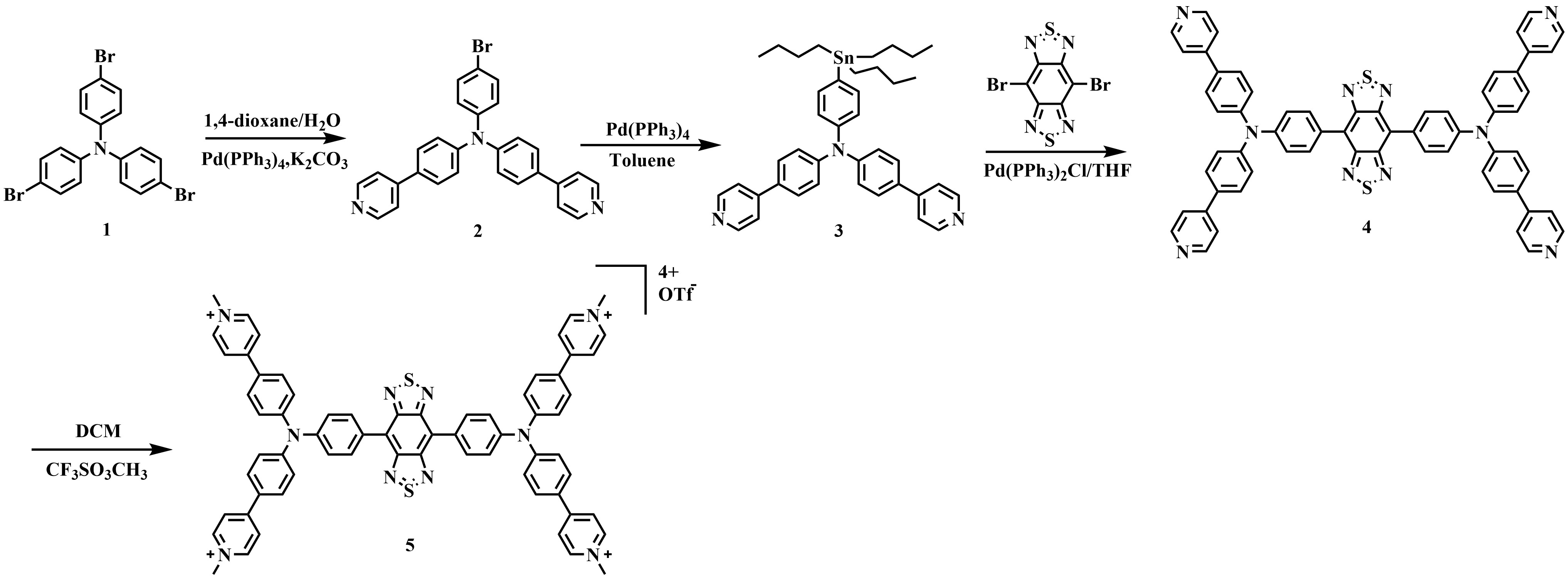


**Figure S1.** Synthesis procedure of the sonosensitizer BT.


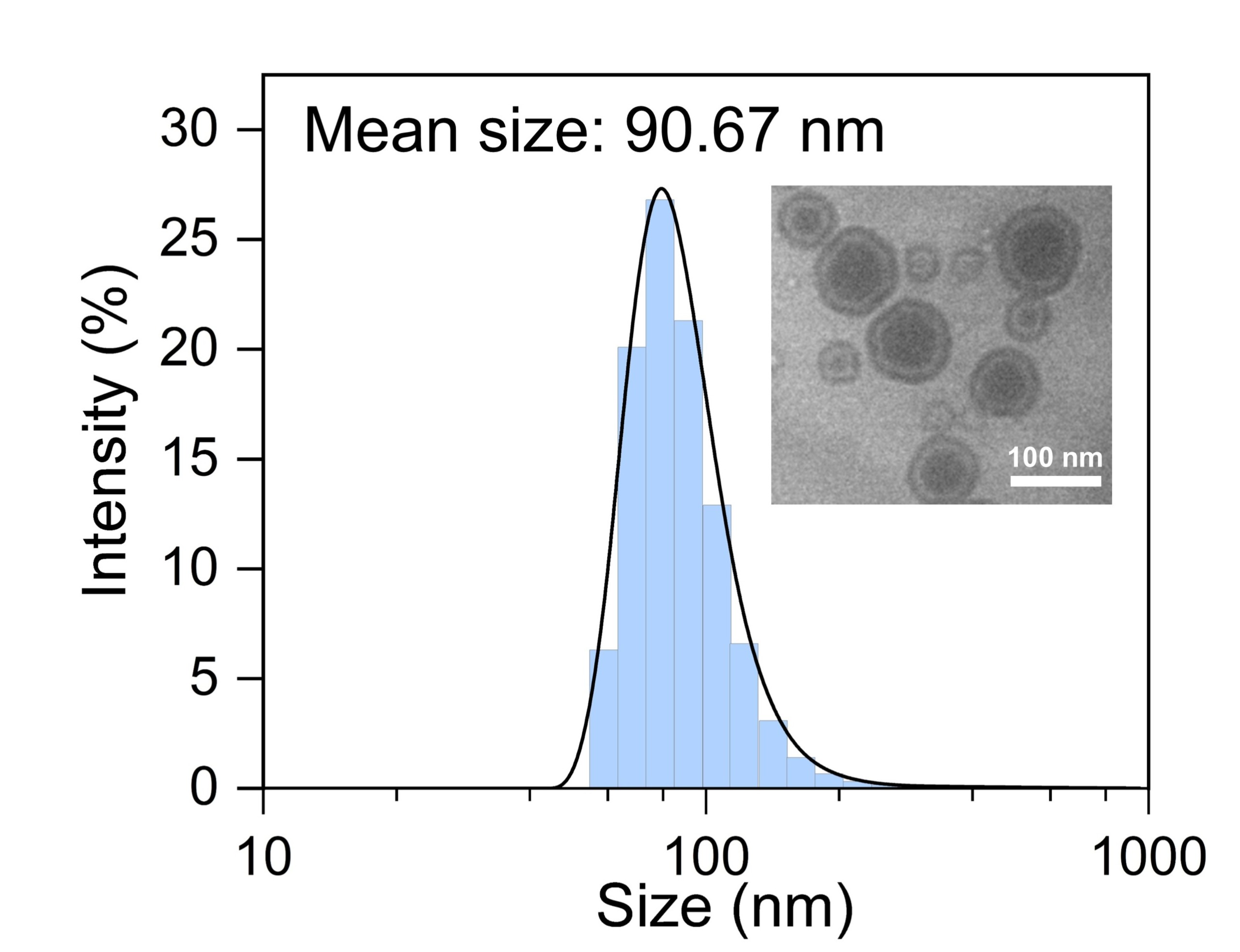


**Figure S2.** DLS and TEM images of BT@Lip showing its morphology and size. Scale bar: 100 nm.


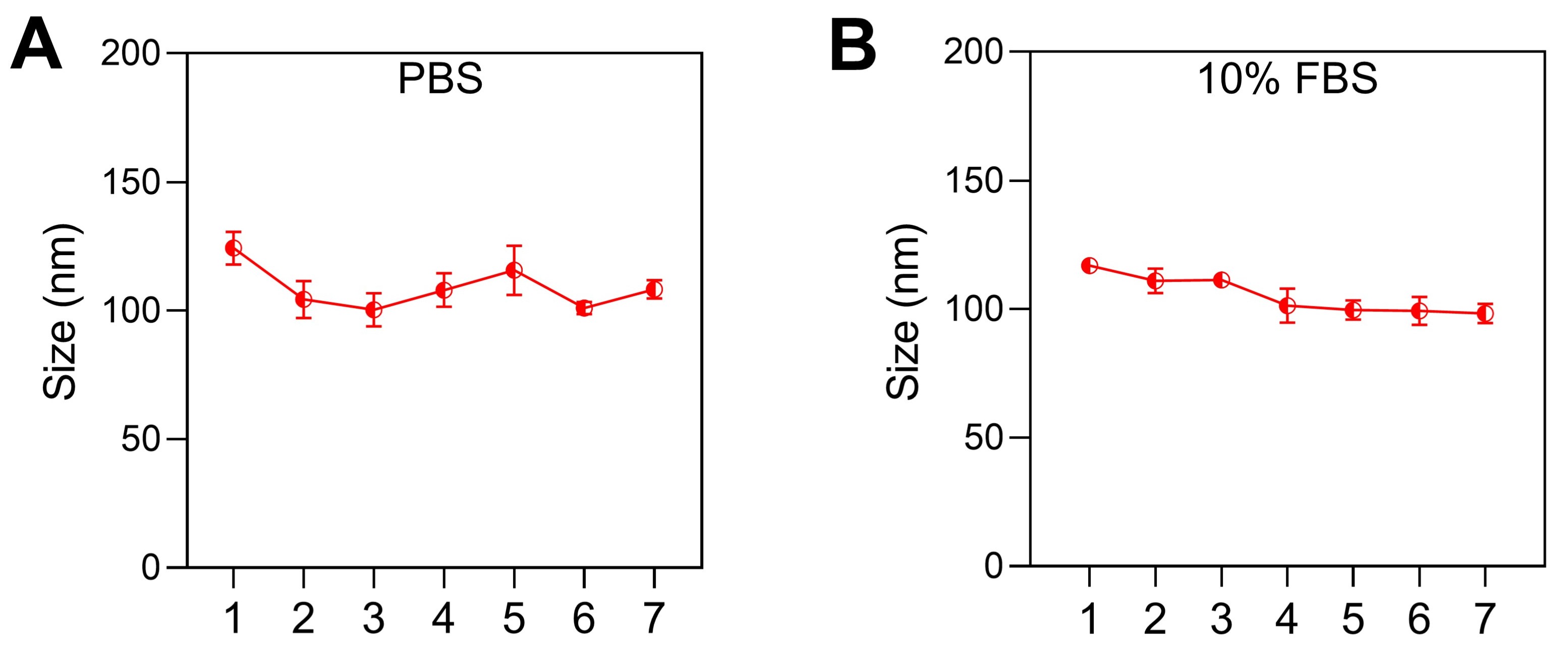


**Figure S3.**The particle size of BT@Lip-TN in A) PBS and B) 10% FBS measured by DLS in PBS over a 7-day period. Data are presented as mean ± S.E.M. (*n = 3*).


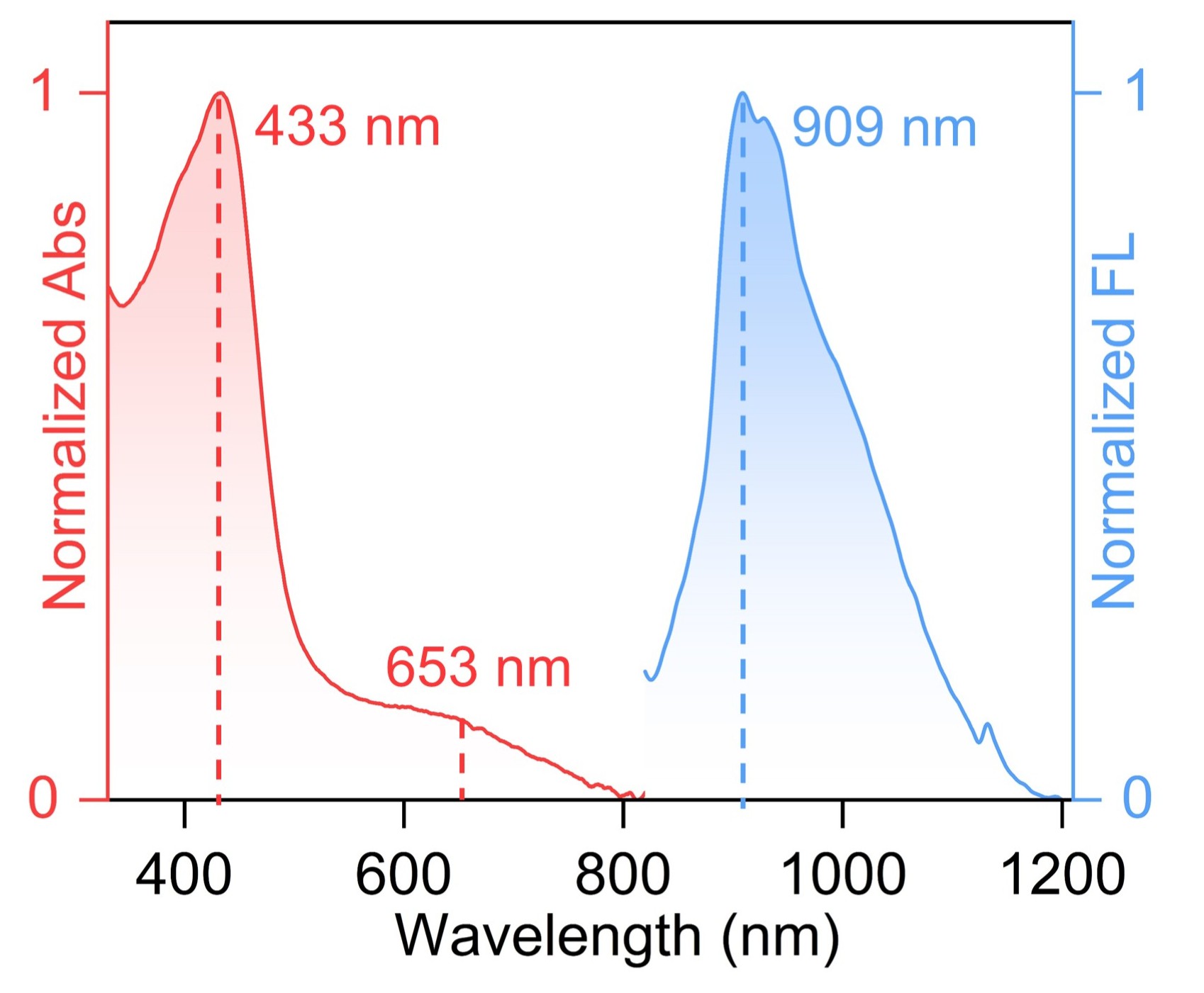


**Figure S4.** Normalized absorption and NIR-II emission (λ_ex_= 808 nm) spectra of BT@Lip.


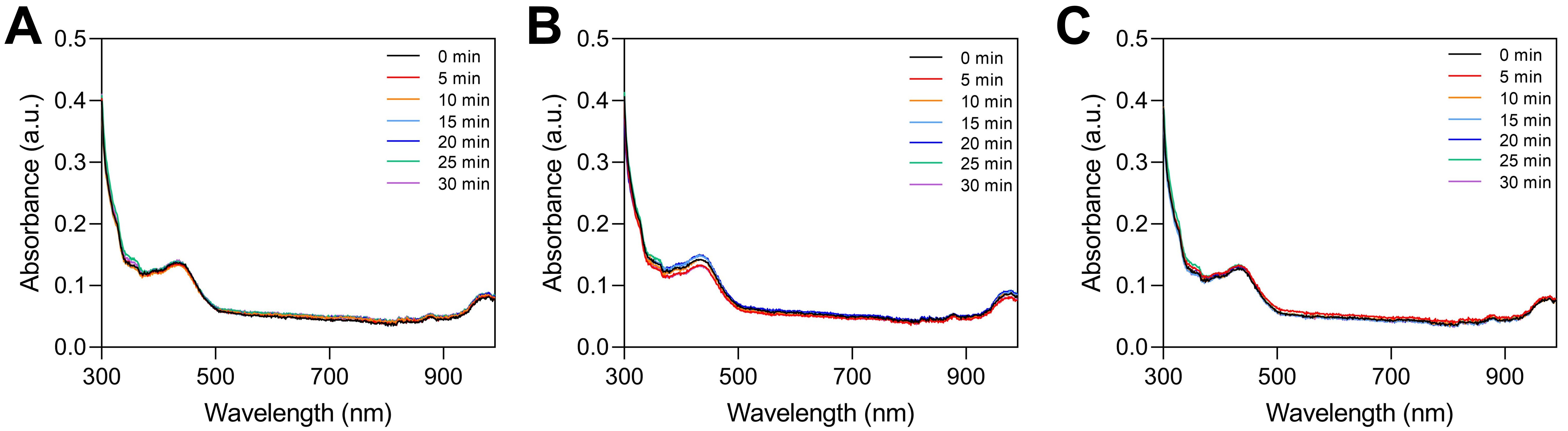


**Figure S5.** Absorption spectra of BT@Lip-TN under ultrasound irradiation at different time points and the intensities of A) 0.2 W cm^-2^, B) 1.0 W cm^-2^, and C) 1.5 W cm^-2^, to evaluate its sonostability.


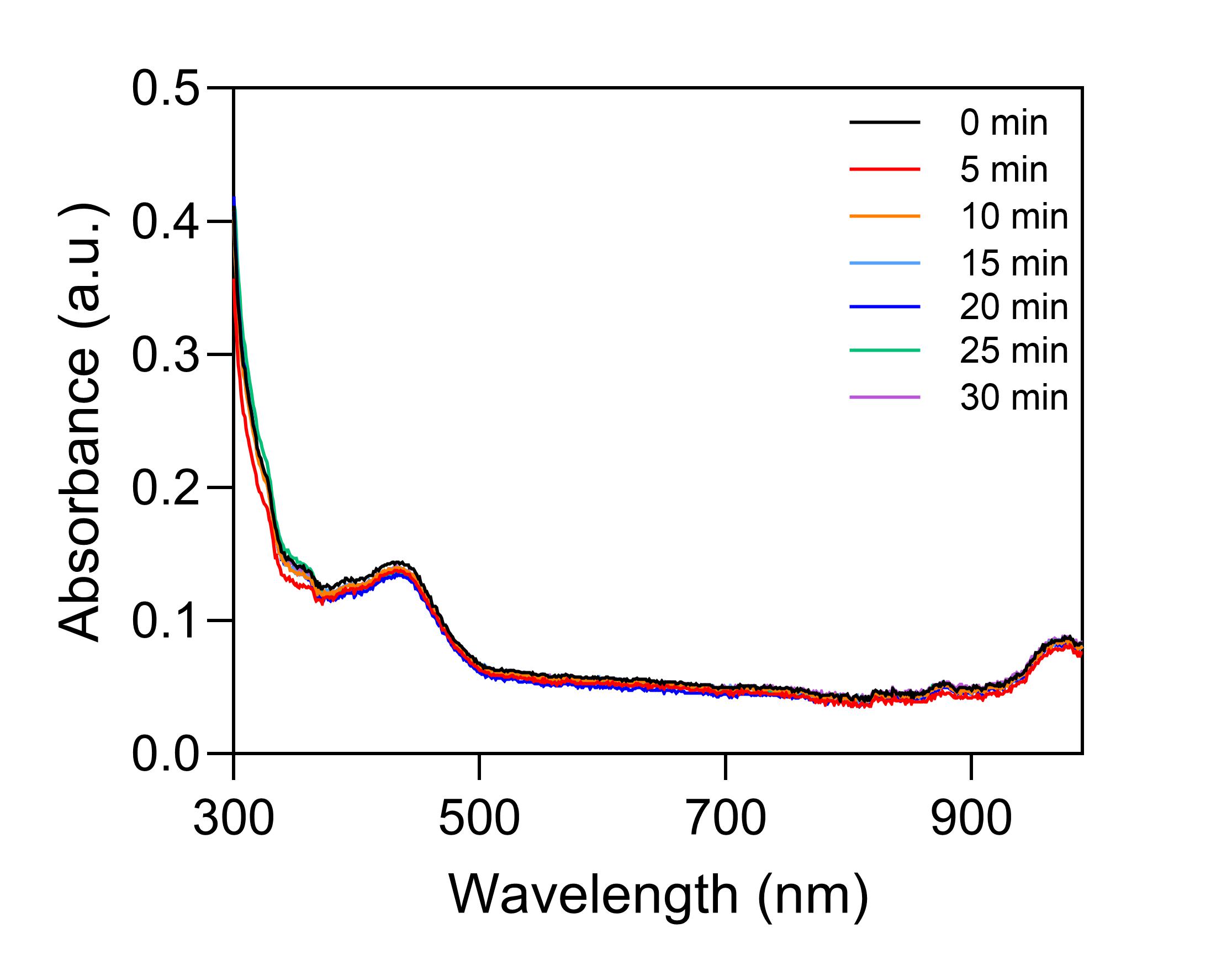


**Figure S6.** Absorption spectra of BT@Lip-TN at different time points under laser irradiation (808 nm, 0.33 W cm^-2^) to assess its photostability.


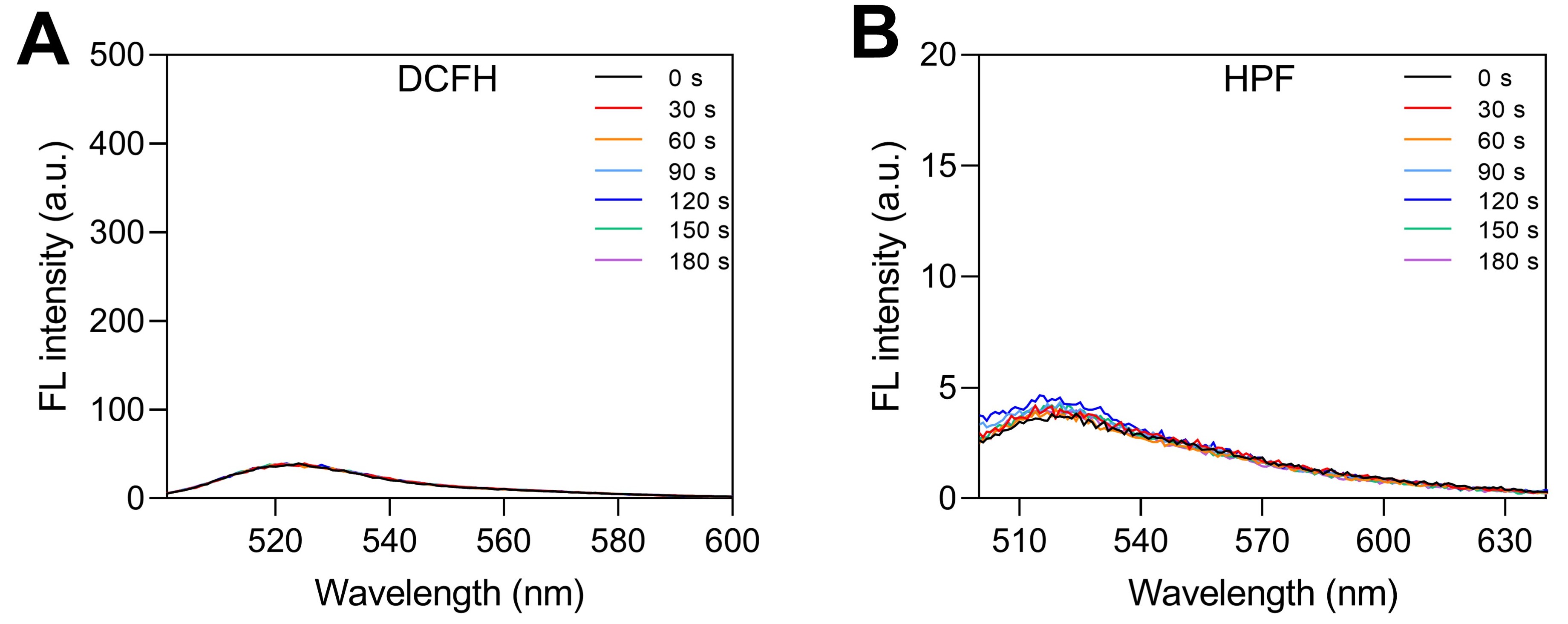


**Figure S7.** Fluorescence spectra of the A) DCFH and B) HPF in water under US irradiation.


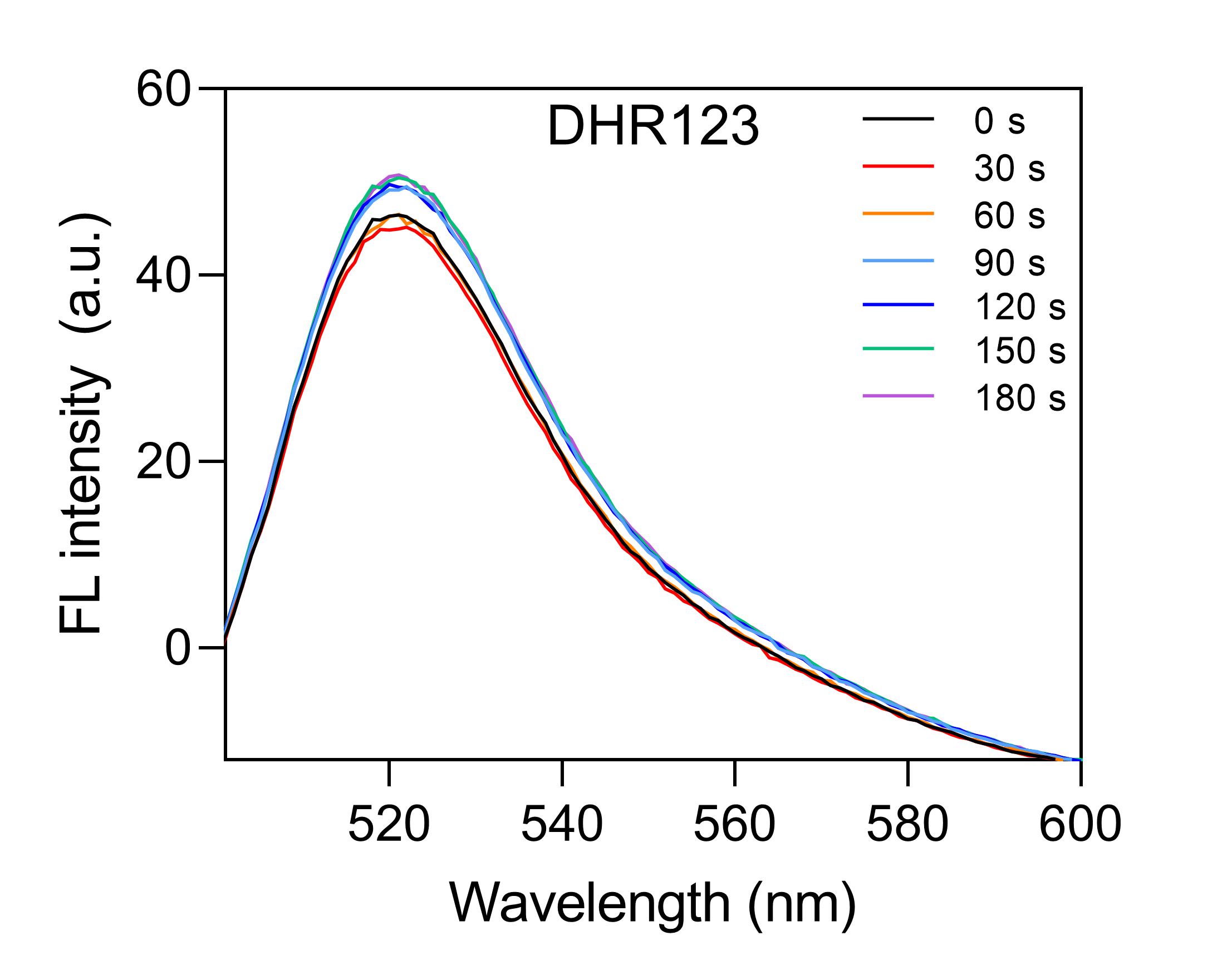


**Figure S8.** Fluorescence spectra of BT@Lip-TN for O_2_^•⁻^ detection using the DHR123 probe.


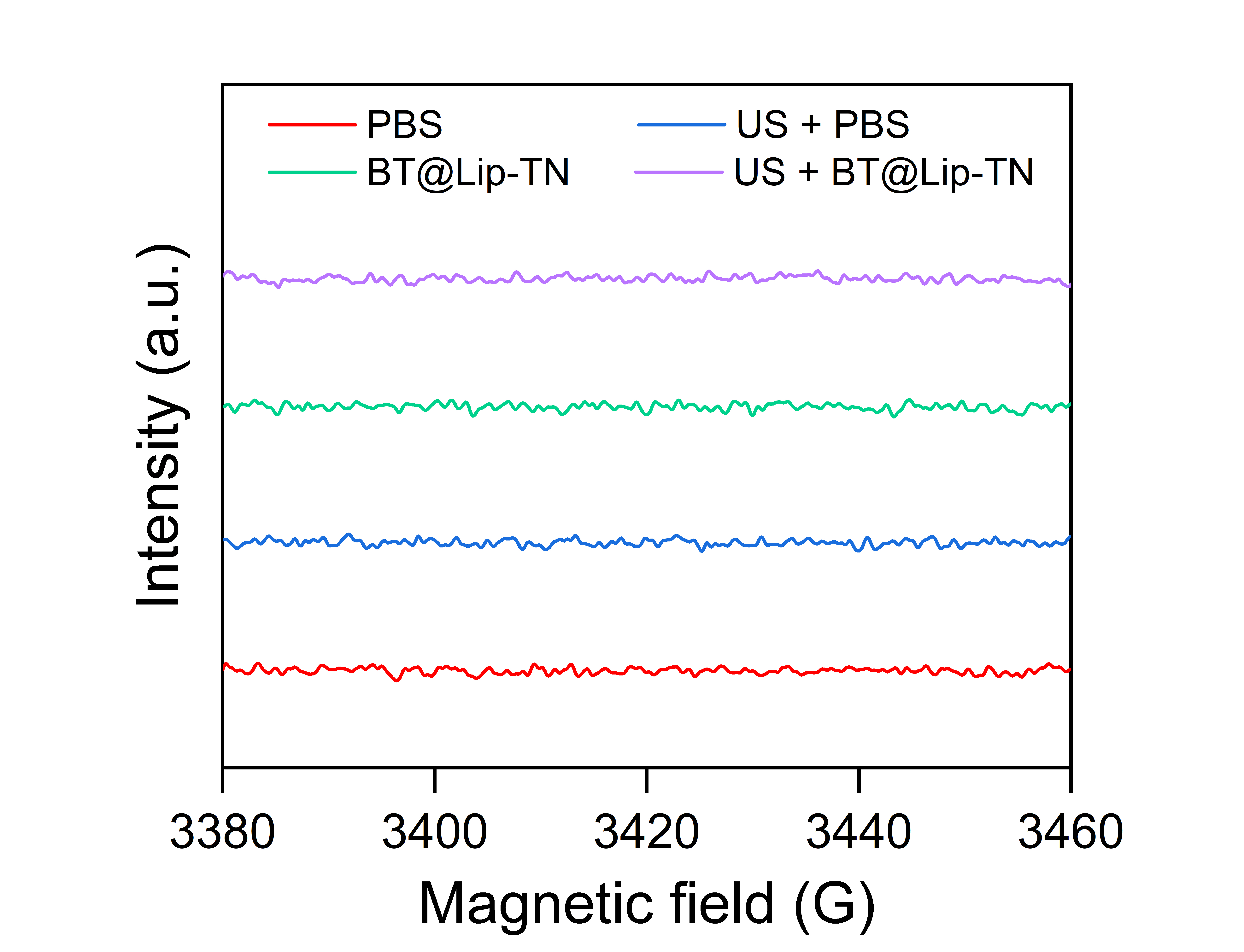


**Figure S9.** ESR spectra using TEMP as the spin trap for ^1^O_2_ detection.


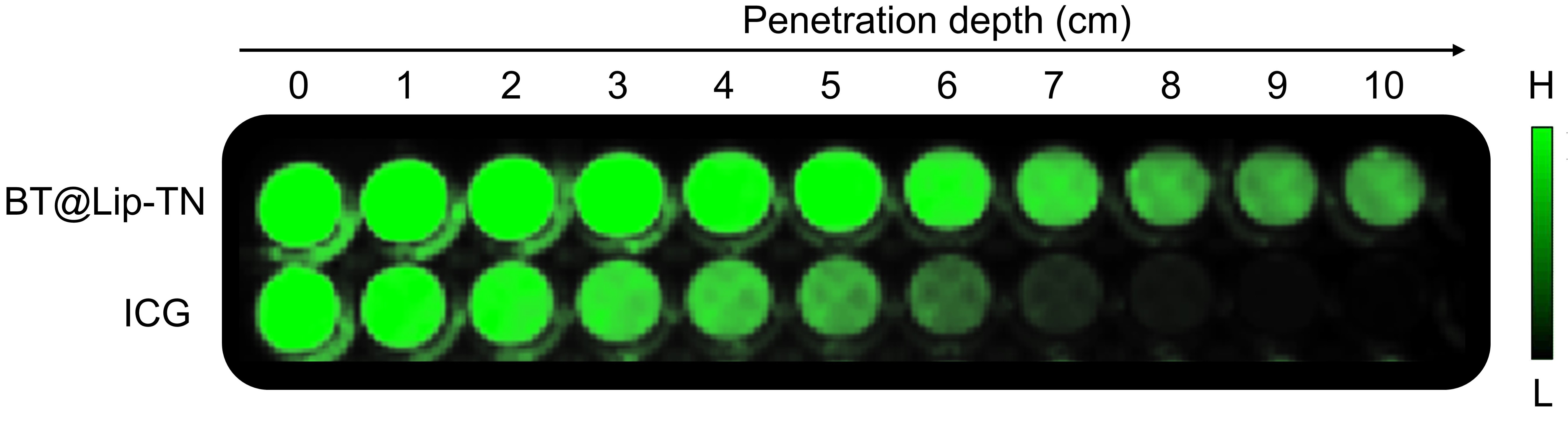


**Figure S10.** Simulation of ROS penetration depth of BT@Lip-TN and ICG in biological tissue-mimicking phantom using DCFH-DA as an indicator.


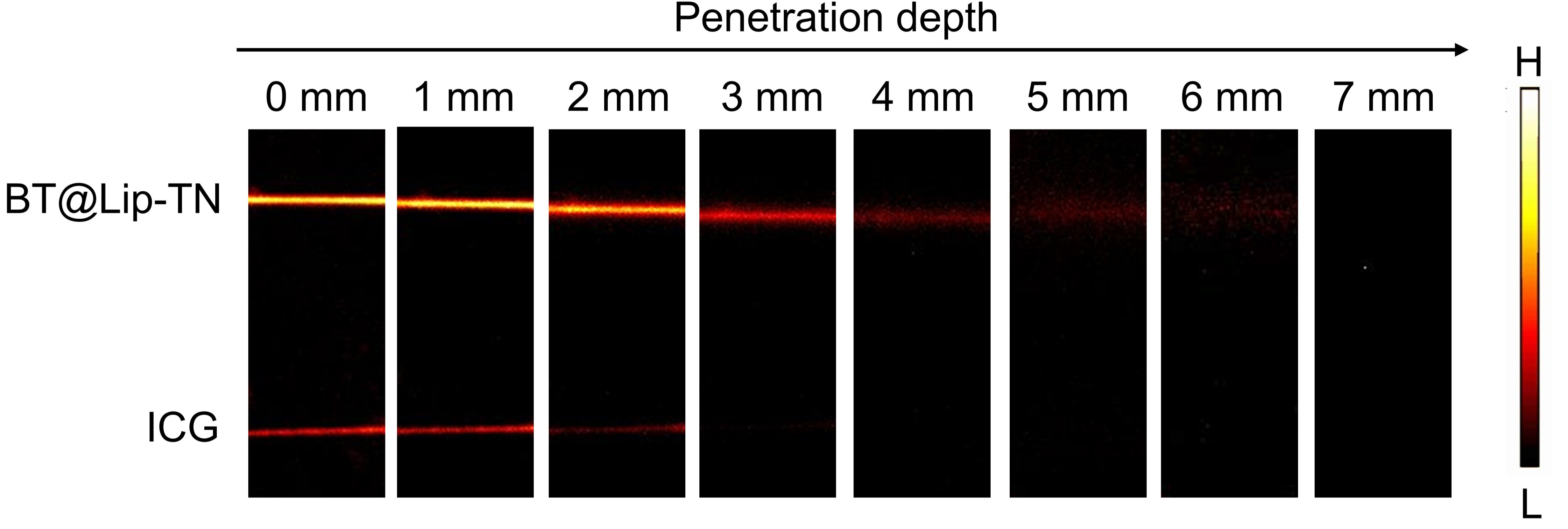


**Figure S11.** Evaluation of fluorescence penetration depth of BT@Lip-TN and ICG (λ_ex_ = 808 nm, long-pass filter: 900 nm).


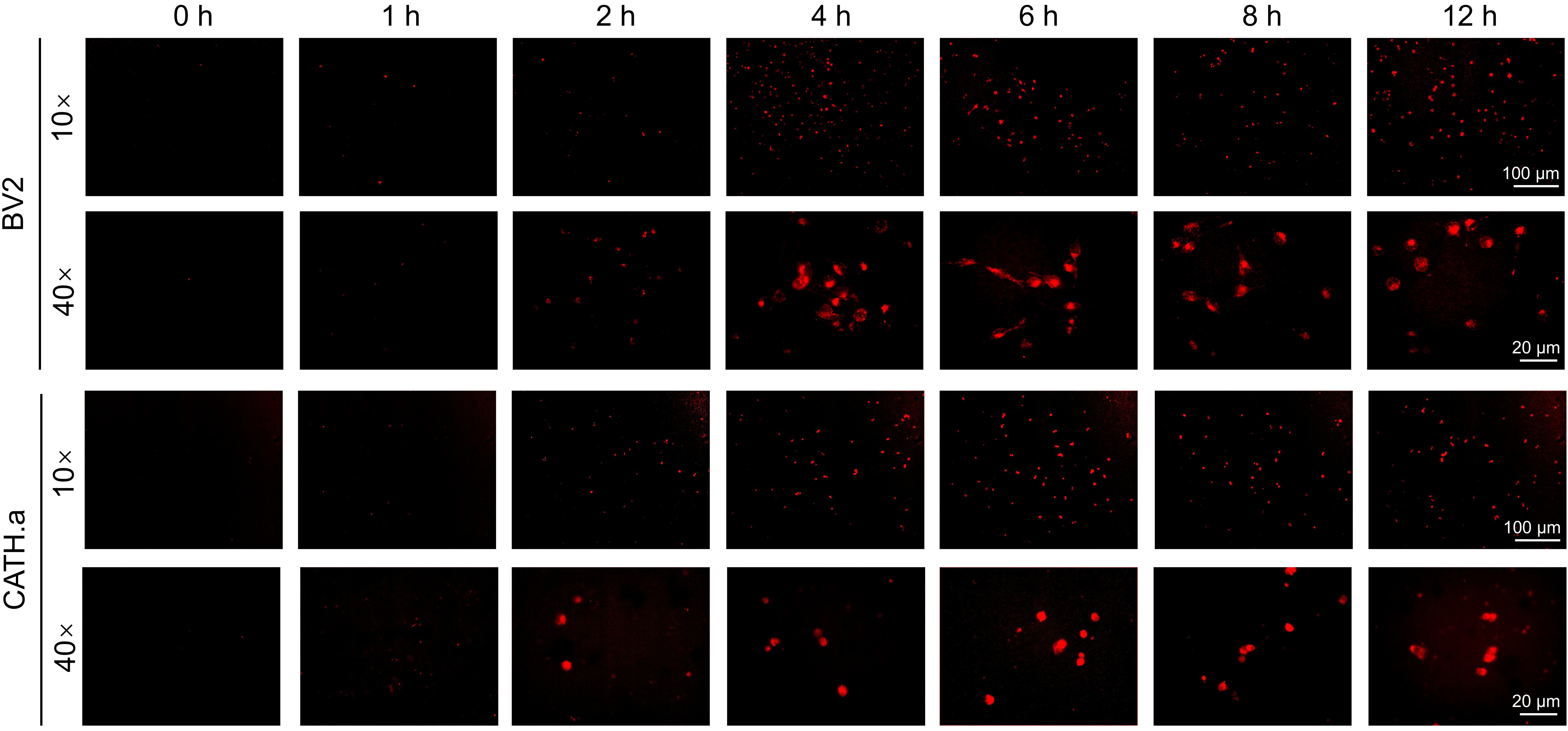


**Figure S12.** NIR-II fluorescence imaging of BV2 and CATH.a cells after incubation with BT@Lip-TN (40 μM) at different time points. Scale bar: 100 μm (above), 20 μm (below).


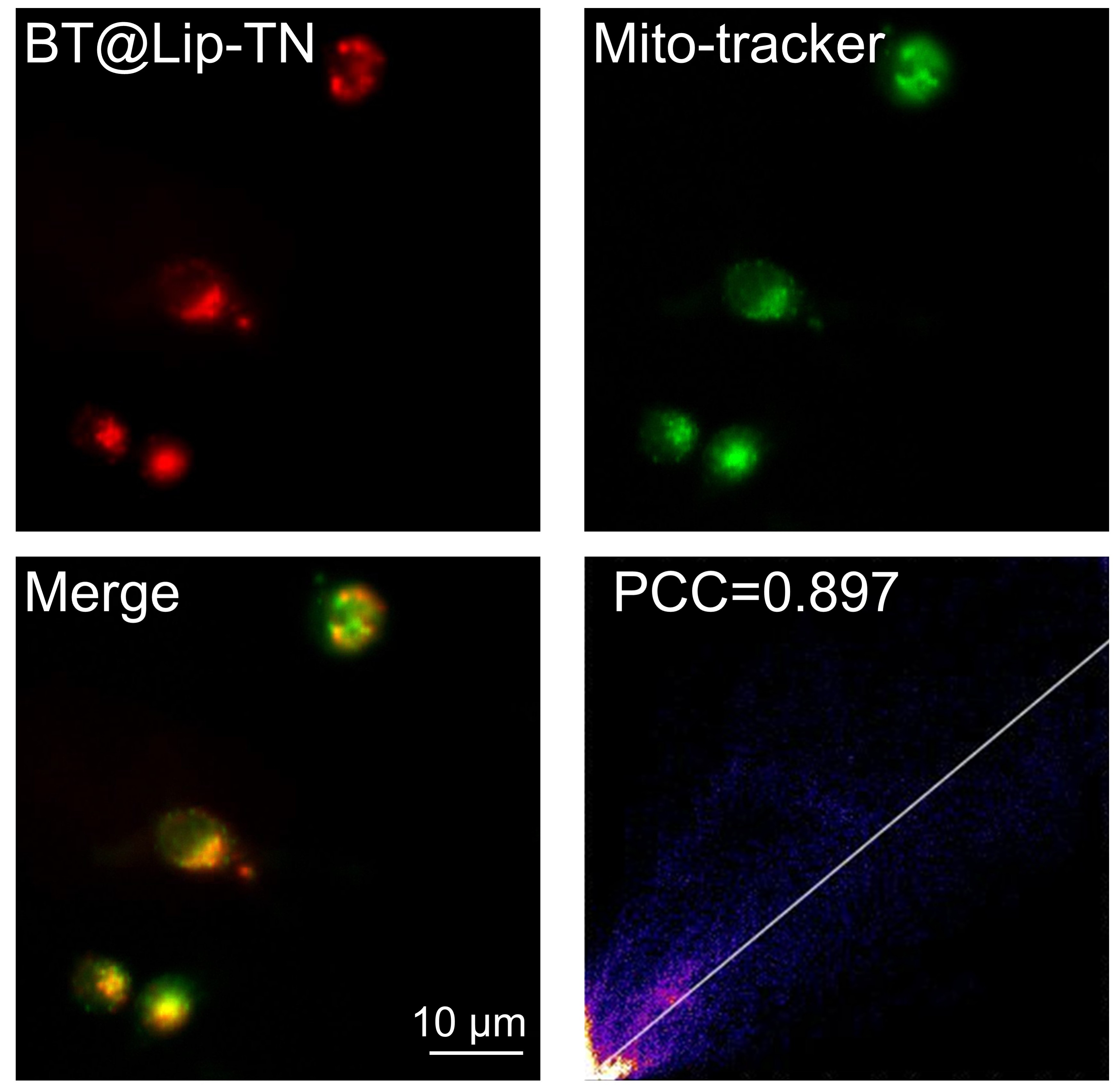


**Figure S13.** Mitochondrial colocalization images of BT@Lip-TN in BV2 cells using an upright microscope. Scale bar: 10 μm.


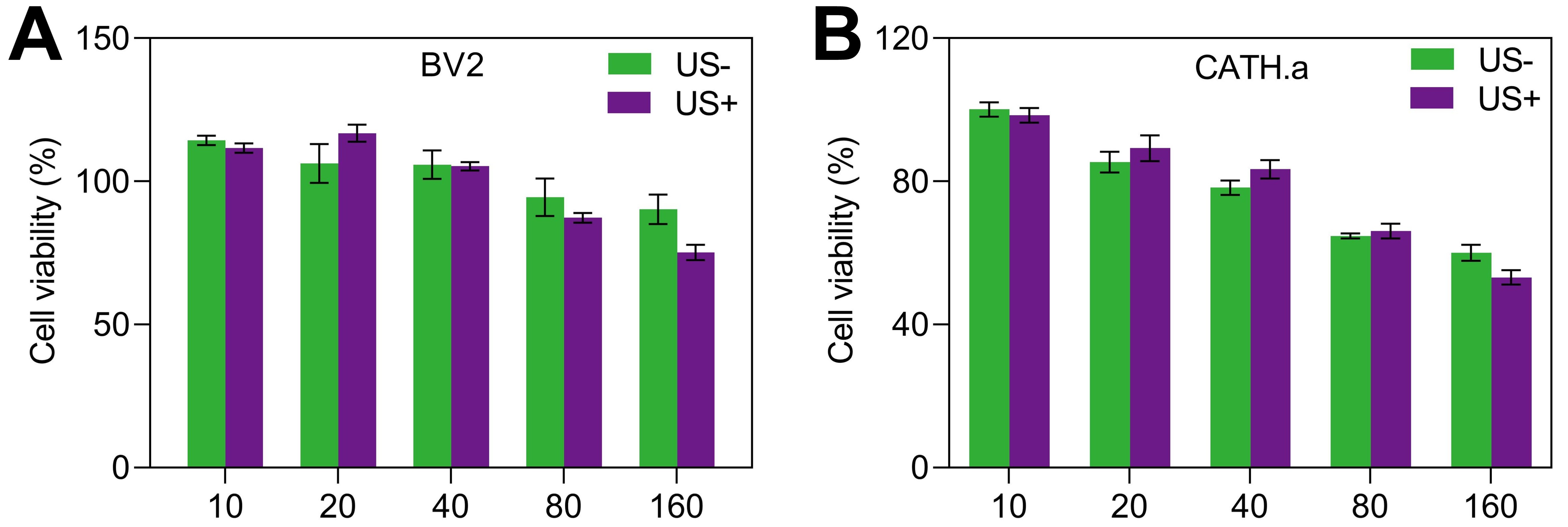


**Figure S14.** Cell viability assessment of A) BV2 and B) CATH.a cells after different treatment conditions using the CCK-8 assay. Data are presented as mean ± S.E.M. (*n = 5*).


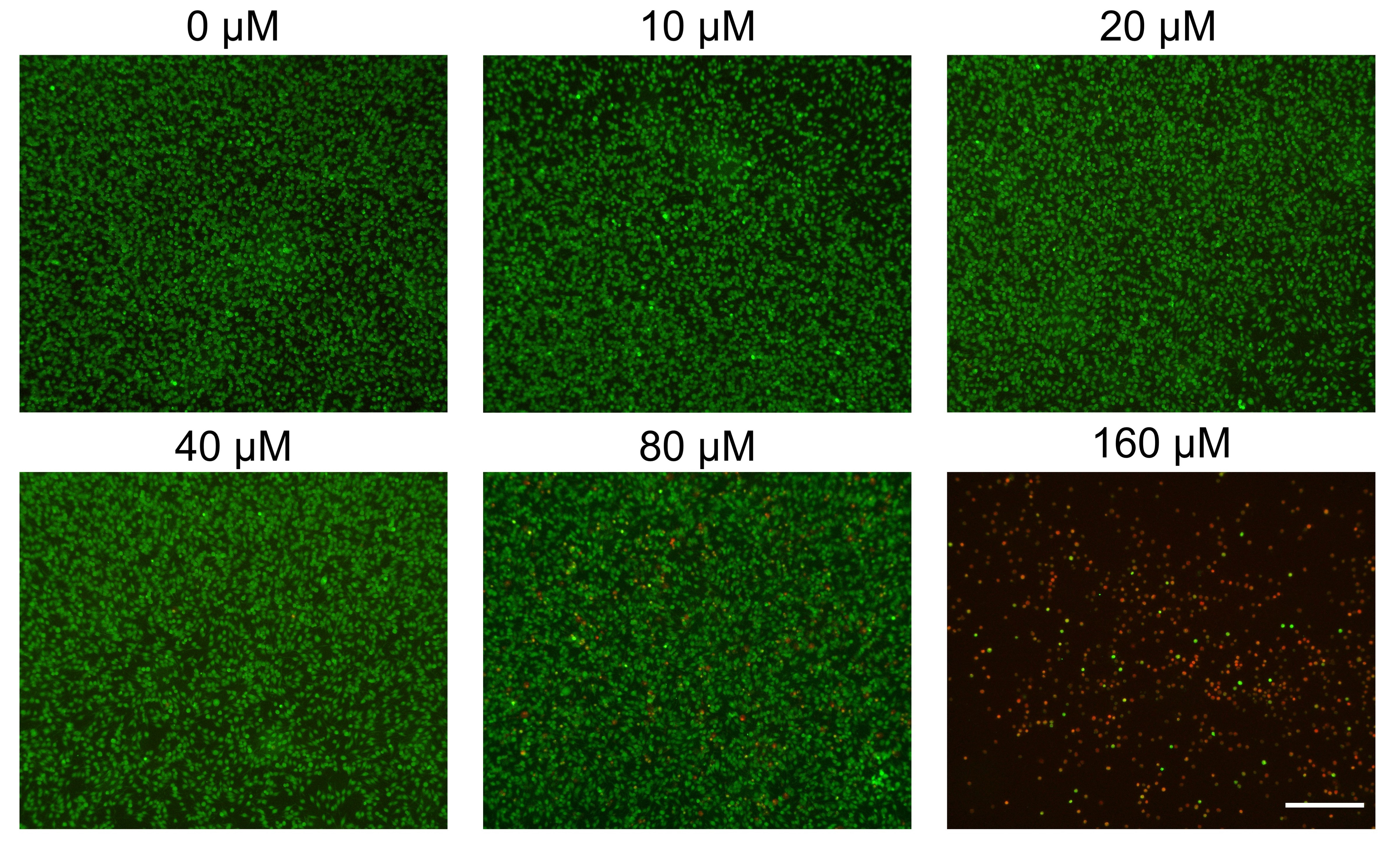


**Figure S15.** Representative images of Calcein-AM/PI staining in BV2 cells after incubation with different concentration of BT@Lip-TN under US irradiation. Scale bar: 100 μm.


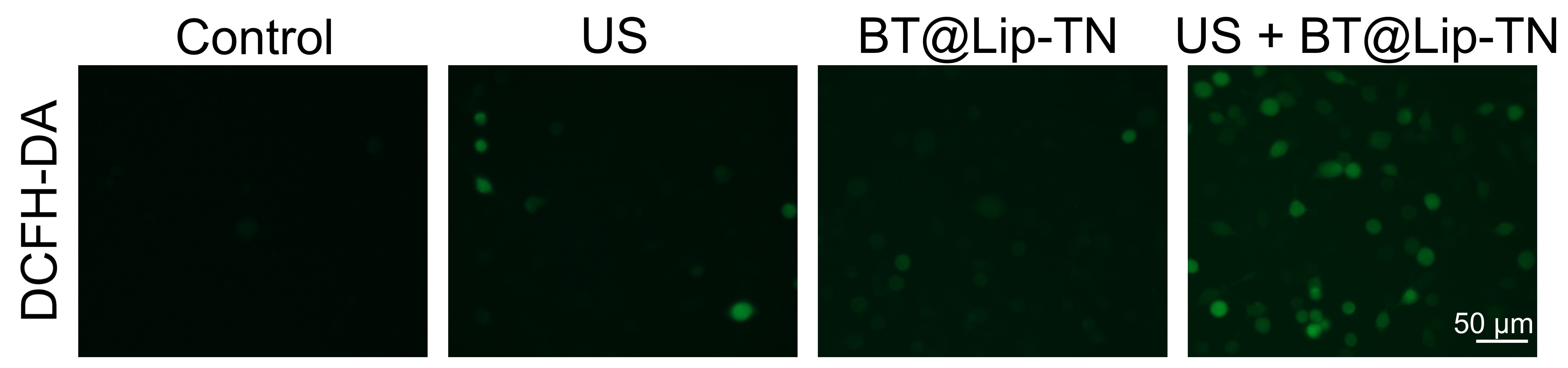


**Figure S16.** Fluorescence images of DCFH-DA-stained BV2 cells under an inverted fluorescence microscope after various treatments. Scale bar: 50 μm.


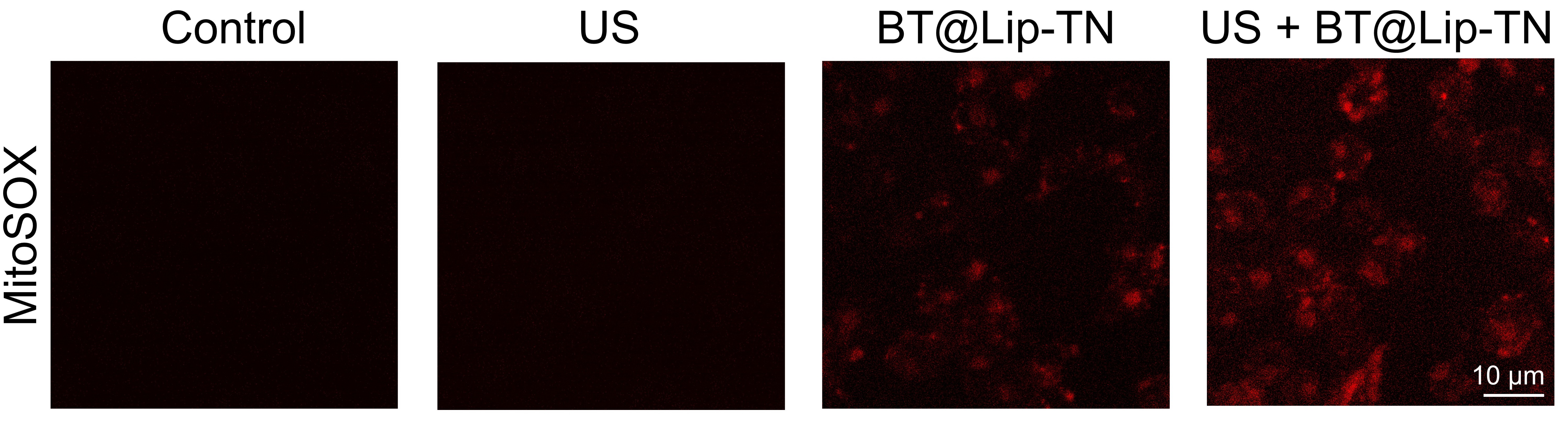


**Figure S17**. Representative fluorescence images of MitoSOX staining in BV2 cells after different treatments. Scale bar: 10 μm.


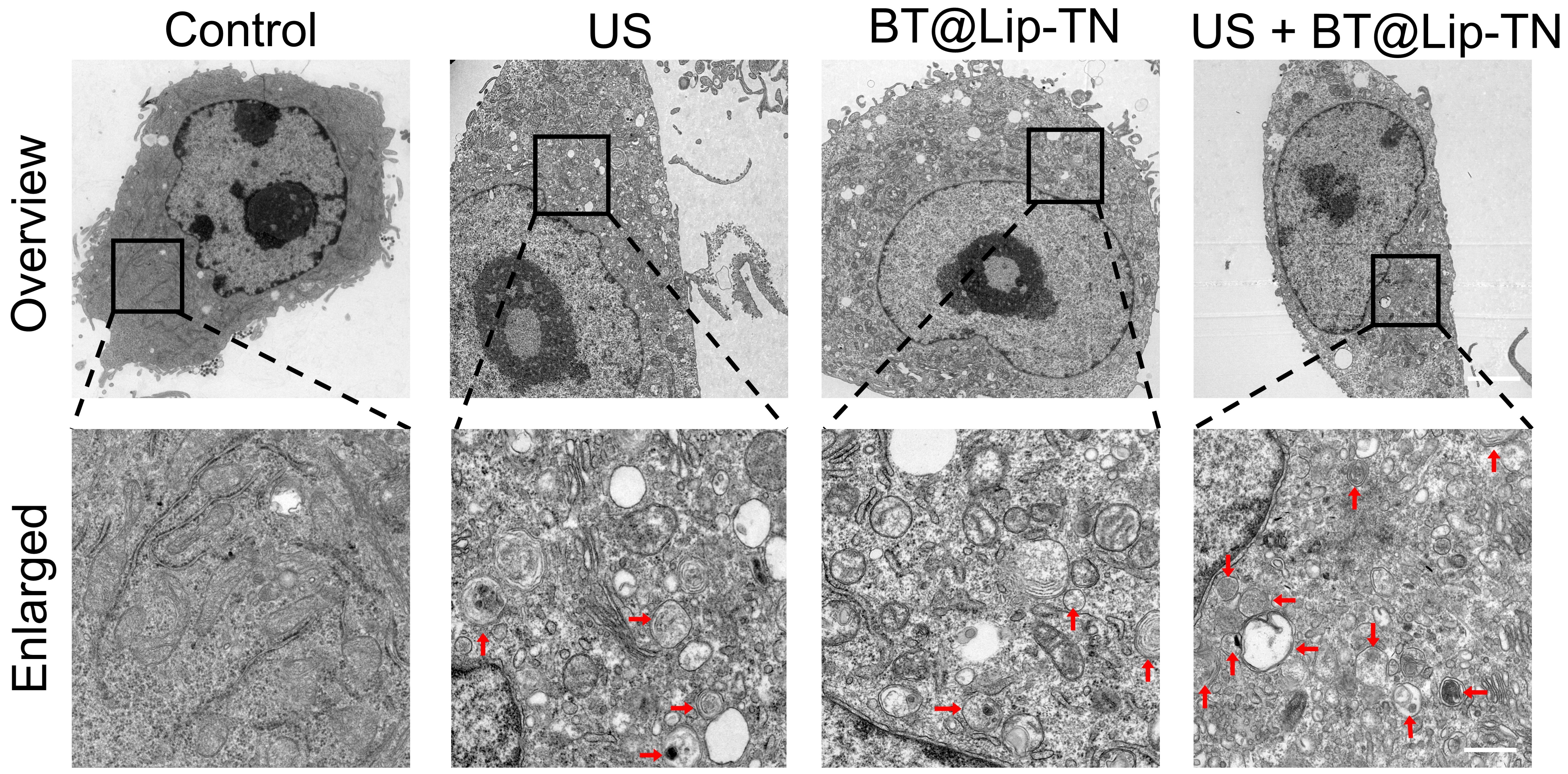


**Figure S18.** Representative Bio-TEM images of BV2 cells under different treatments. Red arrows indicate autolysosomes. Scale bar: 2.5 μm (above), 500 nm (below).

**
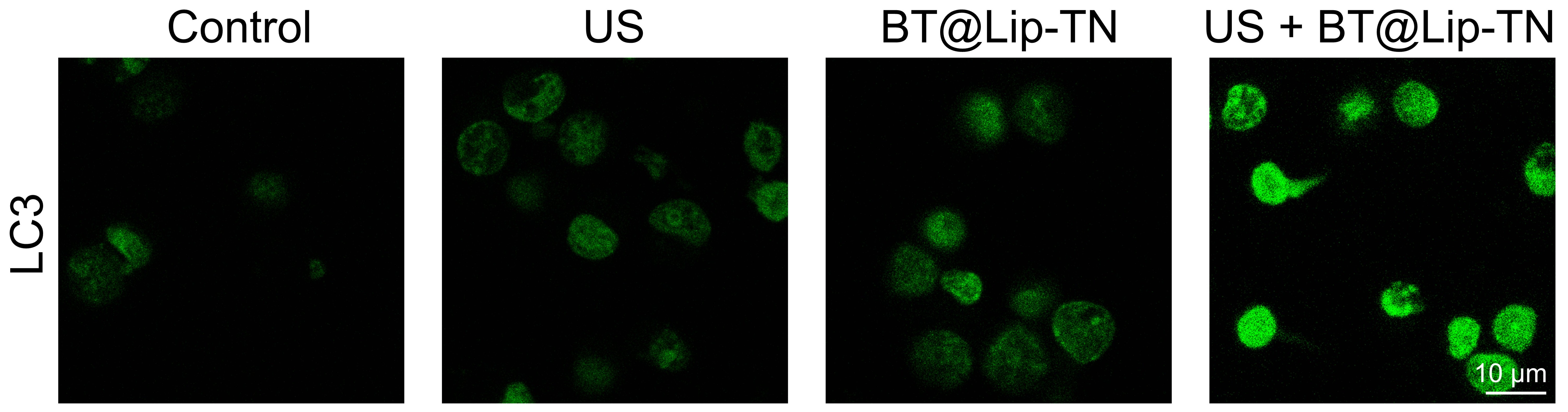
**

**Figure S19.** LC3 immunofluorescence staining in CATH.a cells after various treatments. Scale bar: 10 μm.


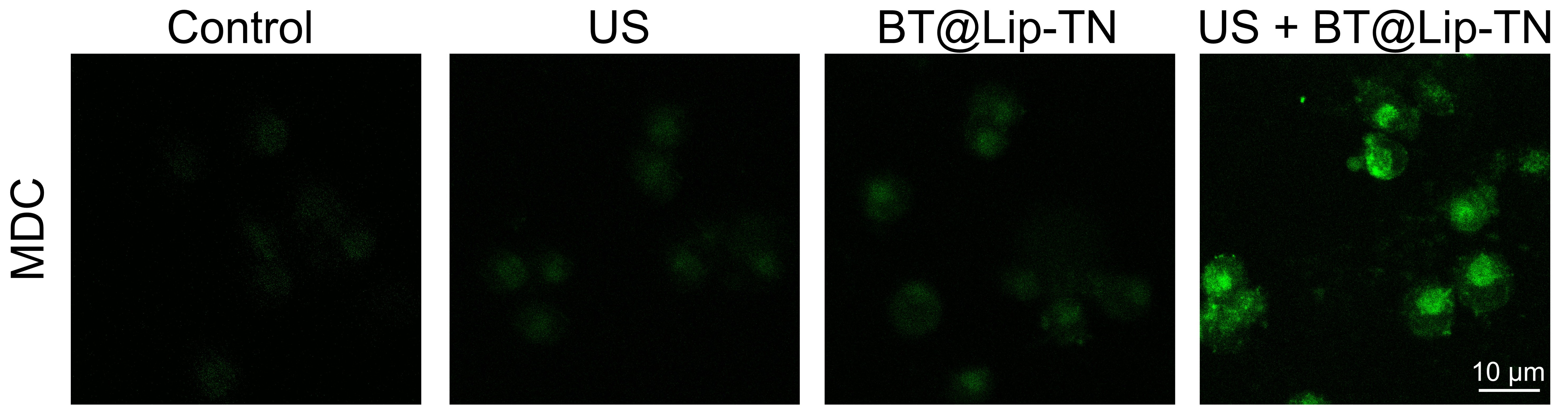


**Figure S20.** MDC staining in differently treated BV2 cells. Scale bar: 10 μm.

**
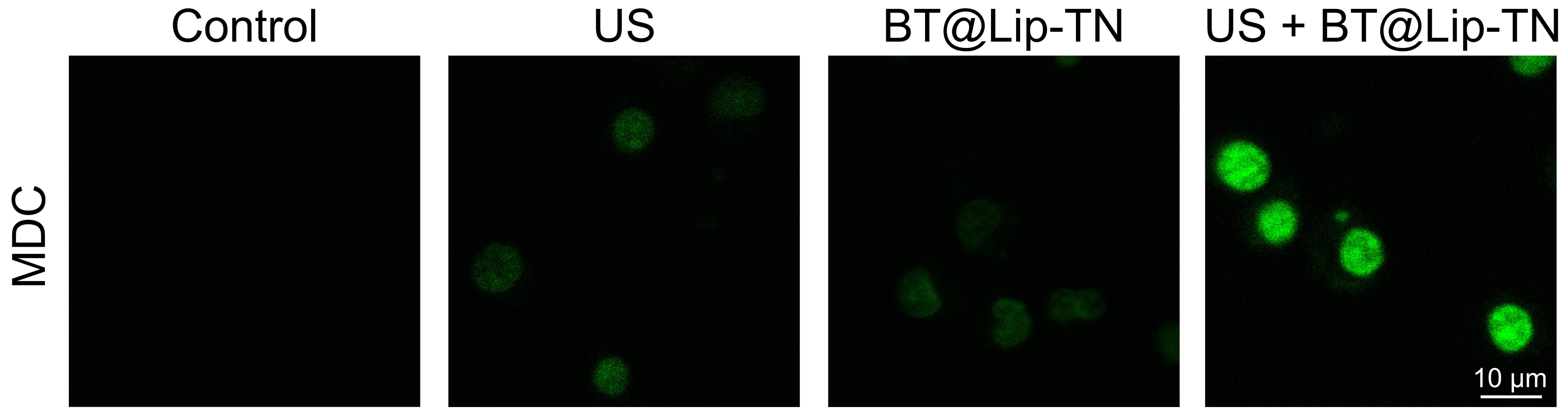
**

**Figure S21.** Confocal images of MDC staining in CATH.a cells under different treatments. Scale bar: 10 μm.


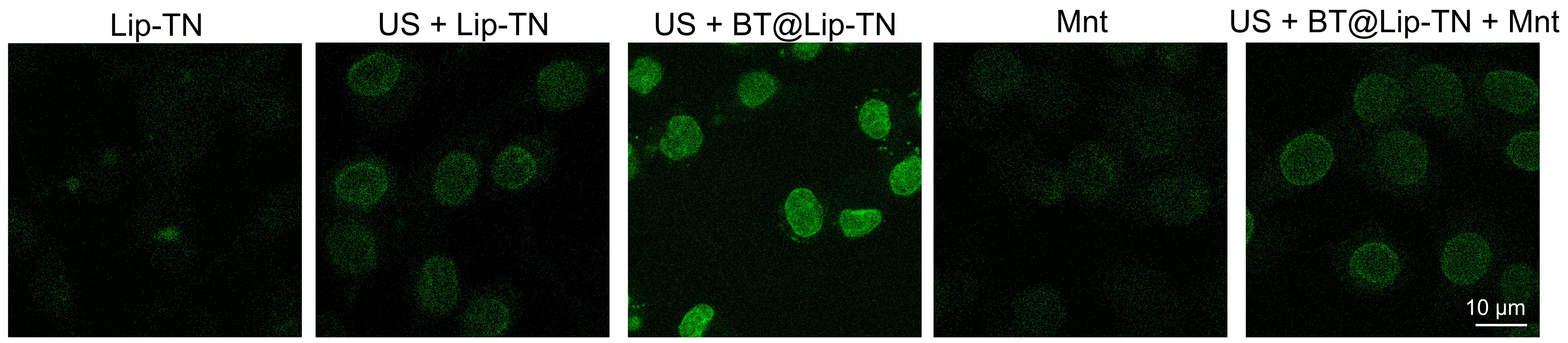


**Figure S22.** LC3 immunofluorescence staining in BV2 cells after the treatment of Lip-TN (2mg/mL), US + Lip-TN (2mg/mL), US + BT@Lip-TN, Mnt (50 mM), and US + BT@Lip-TN + Mnt (50 mM). Scale bar: 10 μm. Mnt, mannitol.


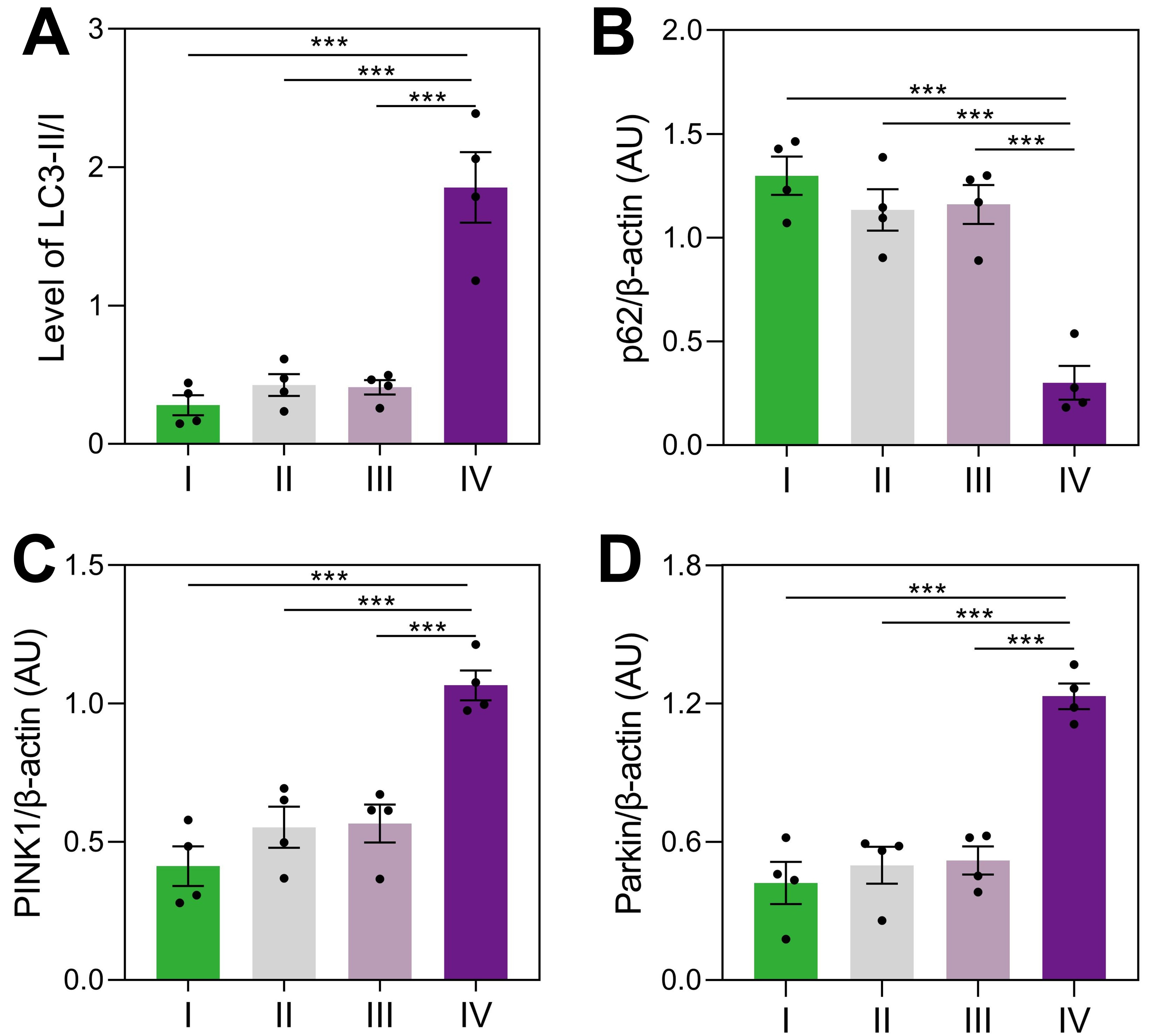


**Figure S23.** Semi-quantitative analysis of A) LC3-II/I, B) p62, C) PINK1, and D) Parkin by Western blot. Data are presented as mean ± S.E.M. (*n = 4*). I, Control; II, US; III, BT@Lip-TN; IV, US + BT@Lip-TN. ****P* < 0.001.


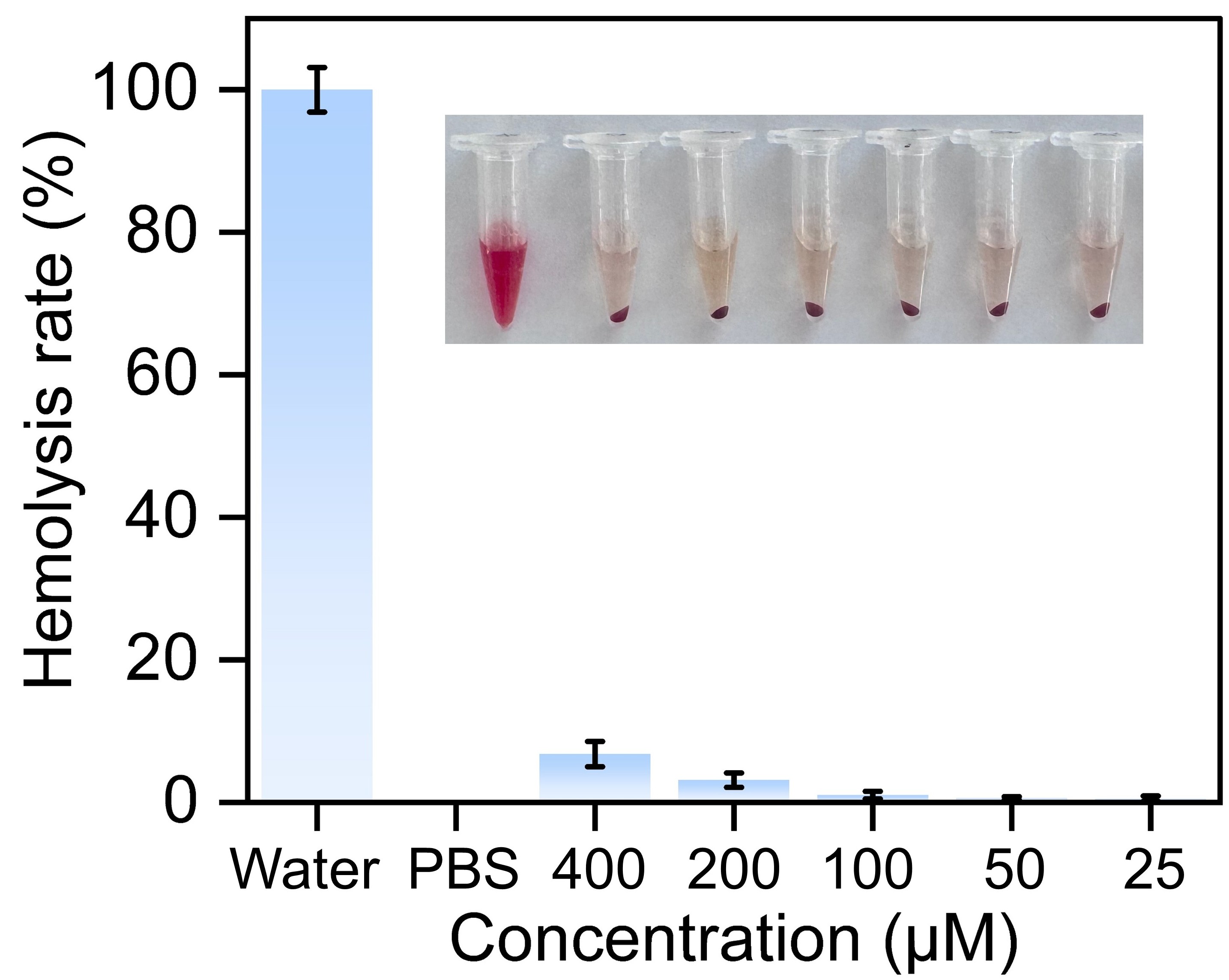


**Figure S24.** Hemolysis rate of red blood cells incubated with different concentrations of BT@Lip-TN (25, 50, 100, 200, 400 μM). The inset shows optical photographs. Values are presented as mean ± S.E.M. (*n = 3*).


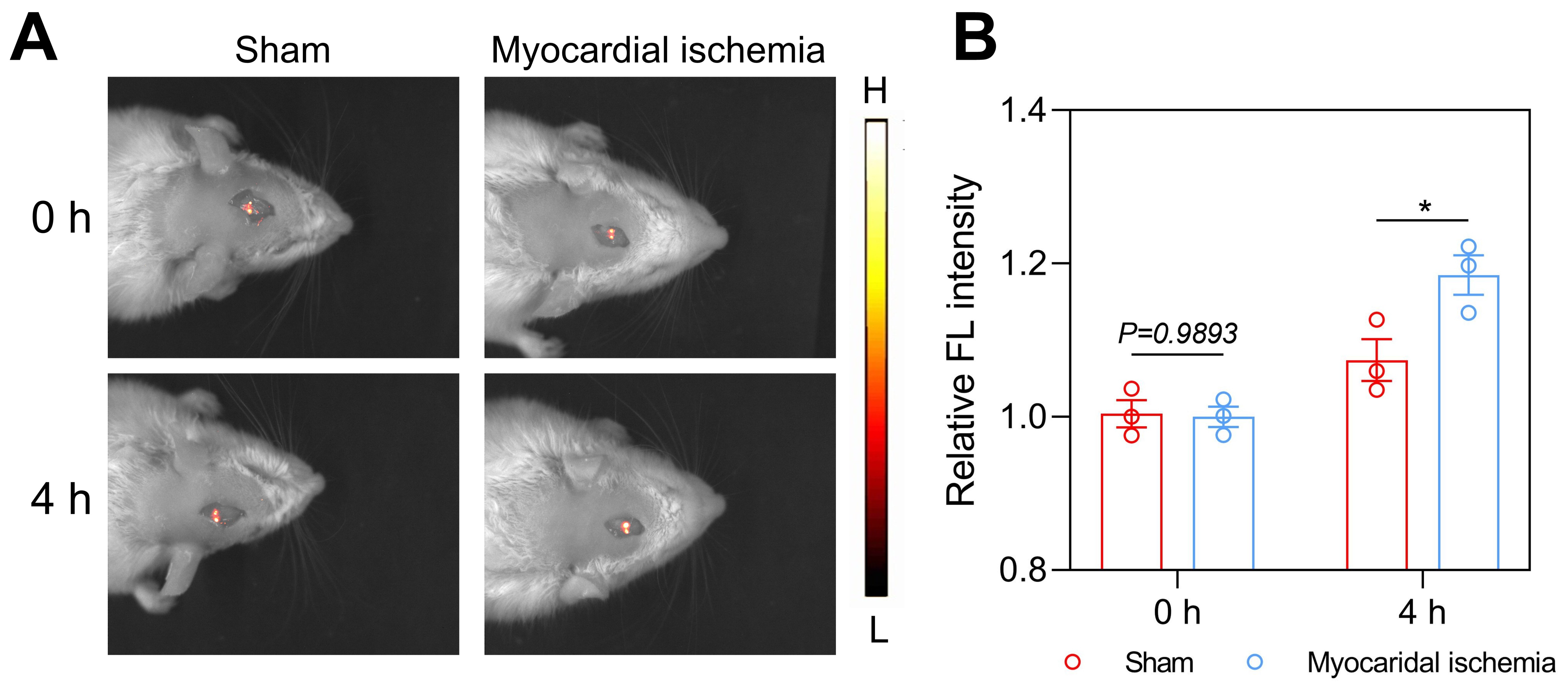


**Figure S25.** A) NIR-II fluorescence images and B) quantitative analysis of the PVN region after myocardial ischemia modeling. Data are presented as mean ± S.E.M. (*n = 3*). **P* < 0.05.


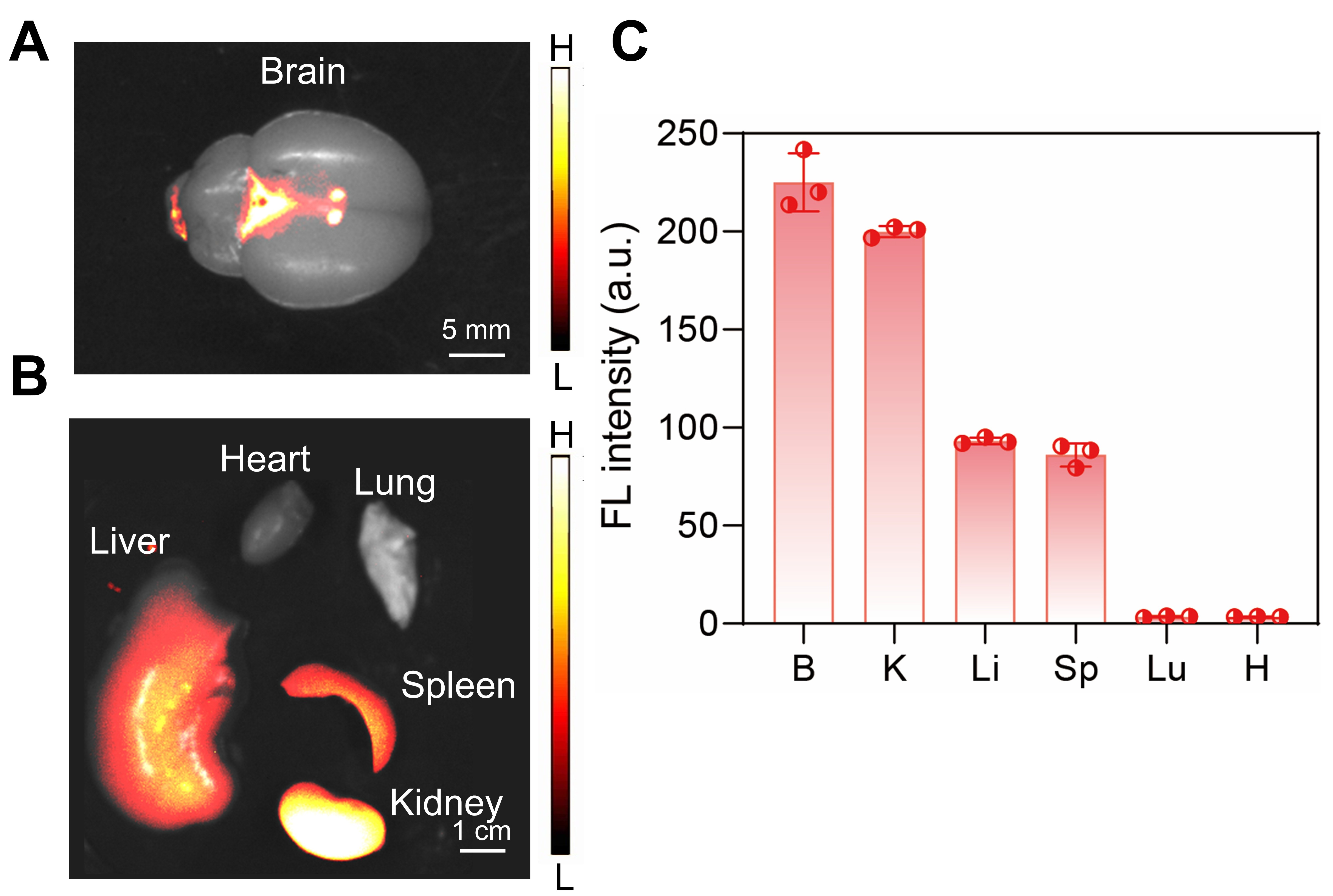


**Figure S26.** *Ex vivo* imaging of major organs and quantitative analysis of fluorescence intensity after microinjection in the PVN. Values are presented as mean ± S.E.M. (*n = 3*). B, brain; K, kidney; Li, liver; Sp, spleen; Lu, lung; H, heart.


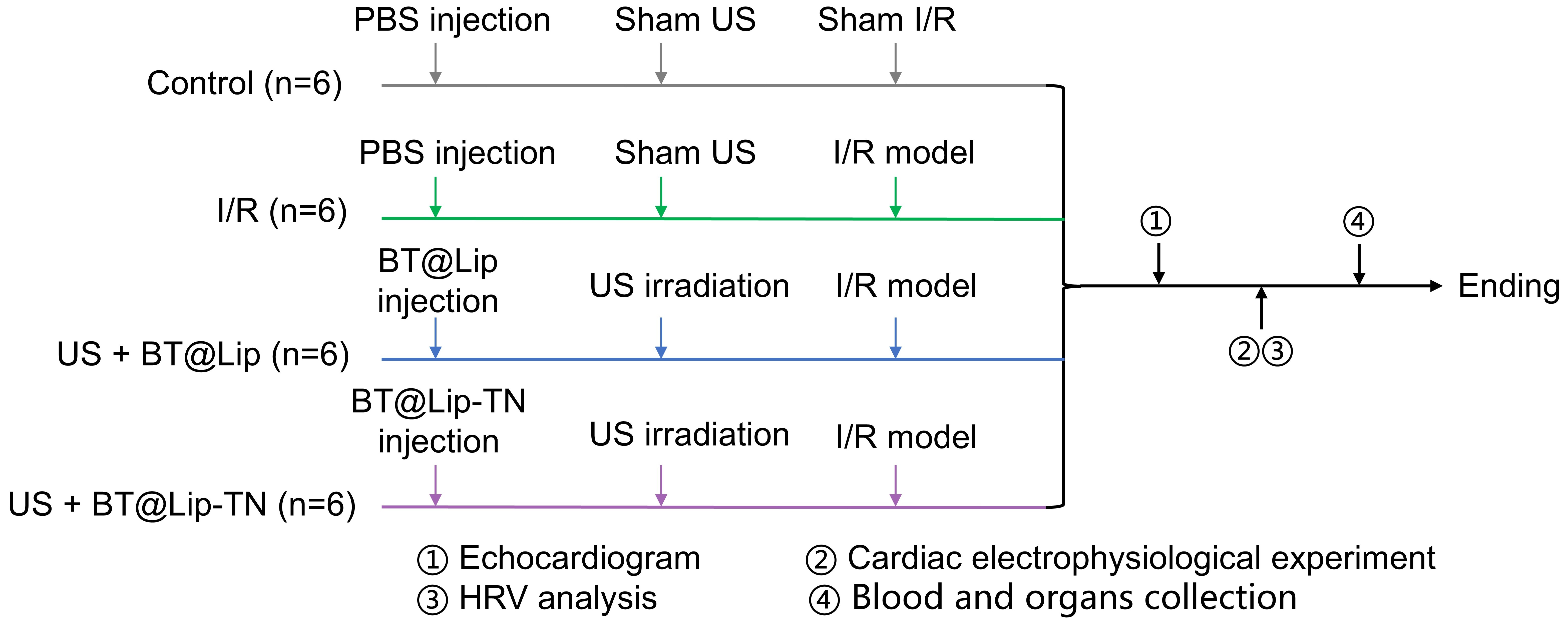


**Figure S27.** Group assignment and detailed experimental flowchart for *in vivo* studies.


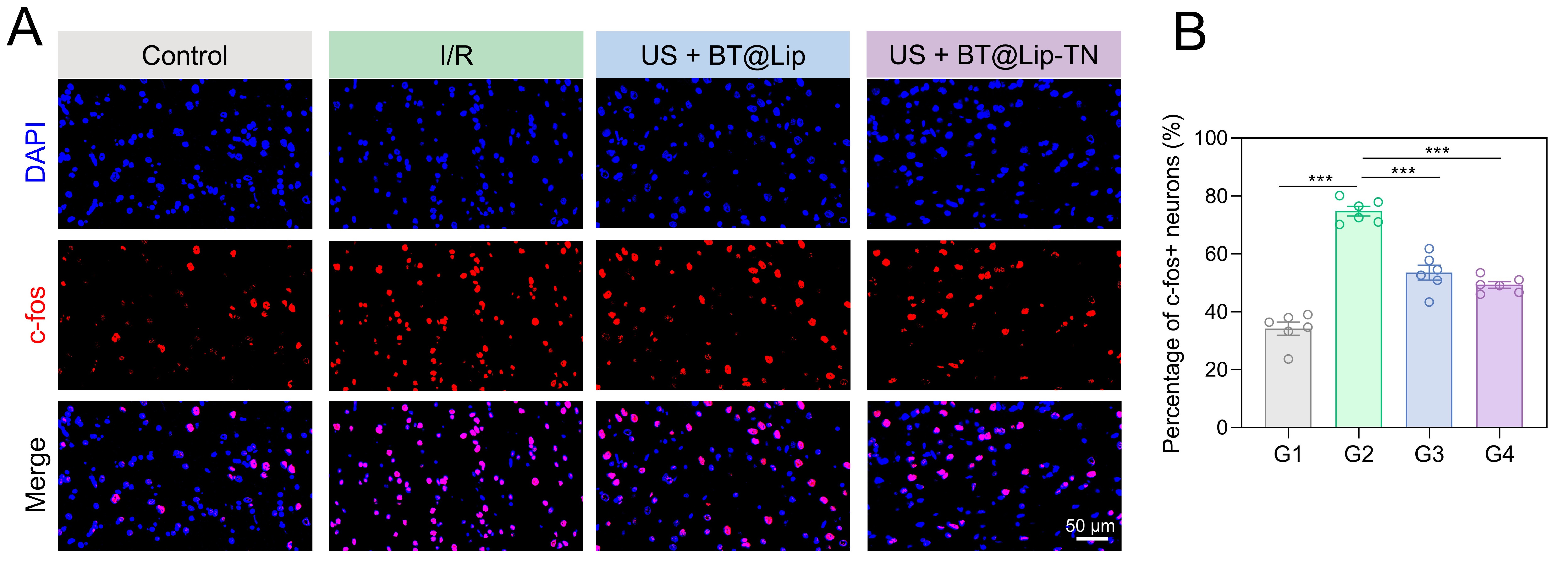


**Figure S28.**  A) Representative images of c-fos immunofluorescence staining in the PVN region. Scale bar: 50 μm. B) Quantitative analysis of the percentage of c-fos^+^ neurons. Data are presented as mean ± S.E.M. (*n = 6*). ****P* < 0.001.


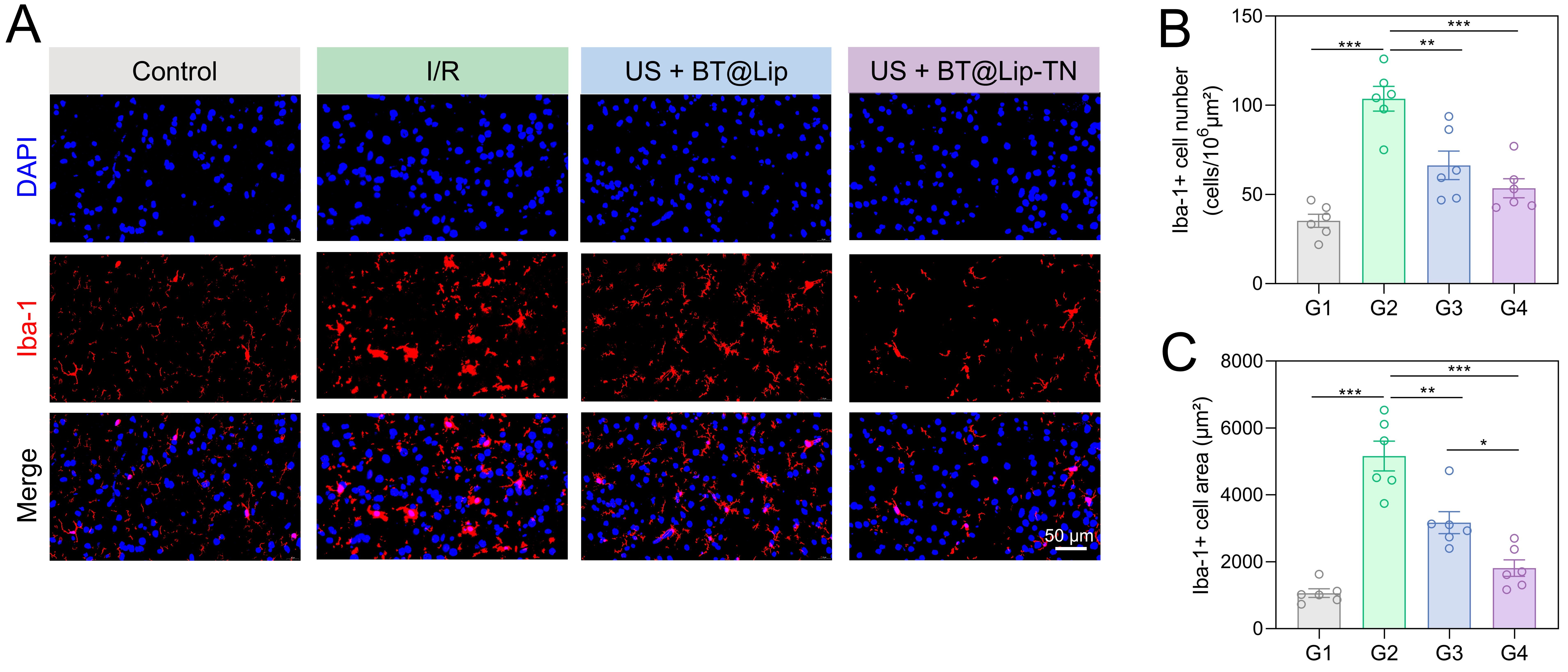


**Figure S29.** A) Typical images of Iba-1 immunofluorescence staining in the PVN region under different treatments. Scale bar: 50 μm. Quantitative analysis of B) Iba-1^+^ cell number and C) cell area. Data are presented as mean ± S.E.M. (*n = 6*). **P* < 0.05, ***P* < 0.01, and ****P* < 0.001.


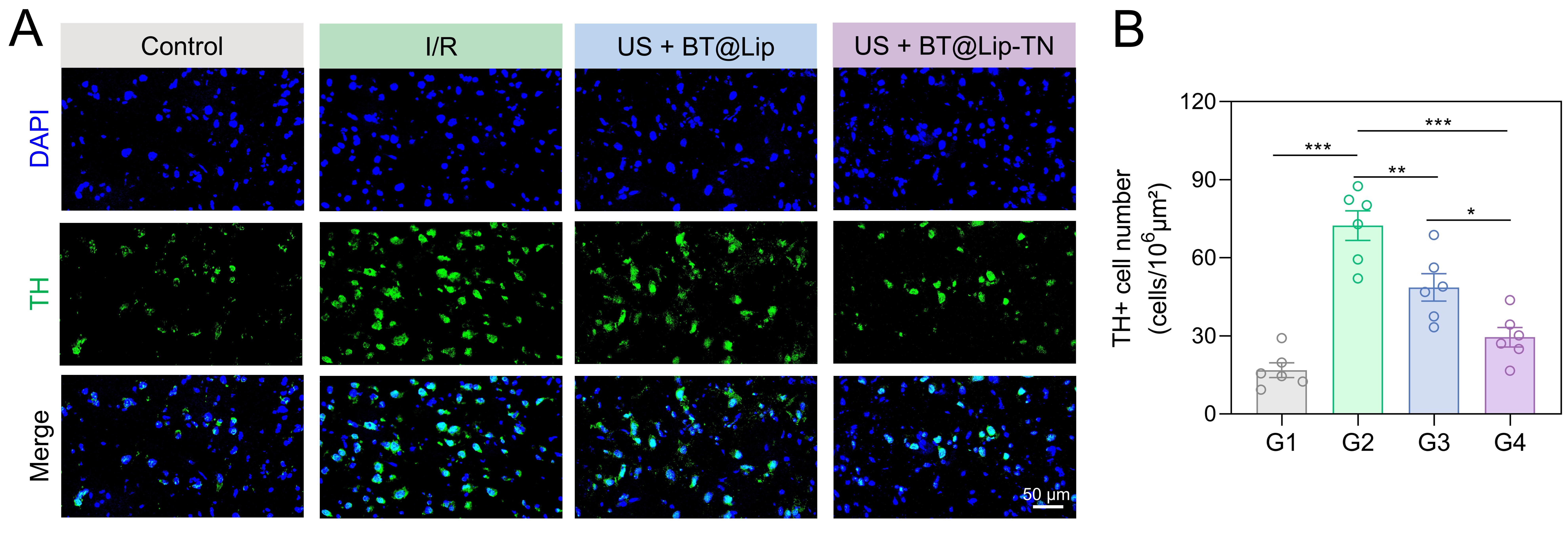


**Figure S30.** A) Representative immunofluorescence images of TH staining within the PVN region. Scale bar: 50 μm. B) Quantification of TH^+^ neurons. Data are presented as mean ± S.E.M. (*n = 6*). **P* < 0.05, ***P* < 0.01, and ****P* < 0.001.


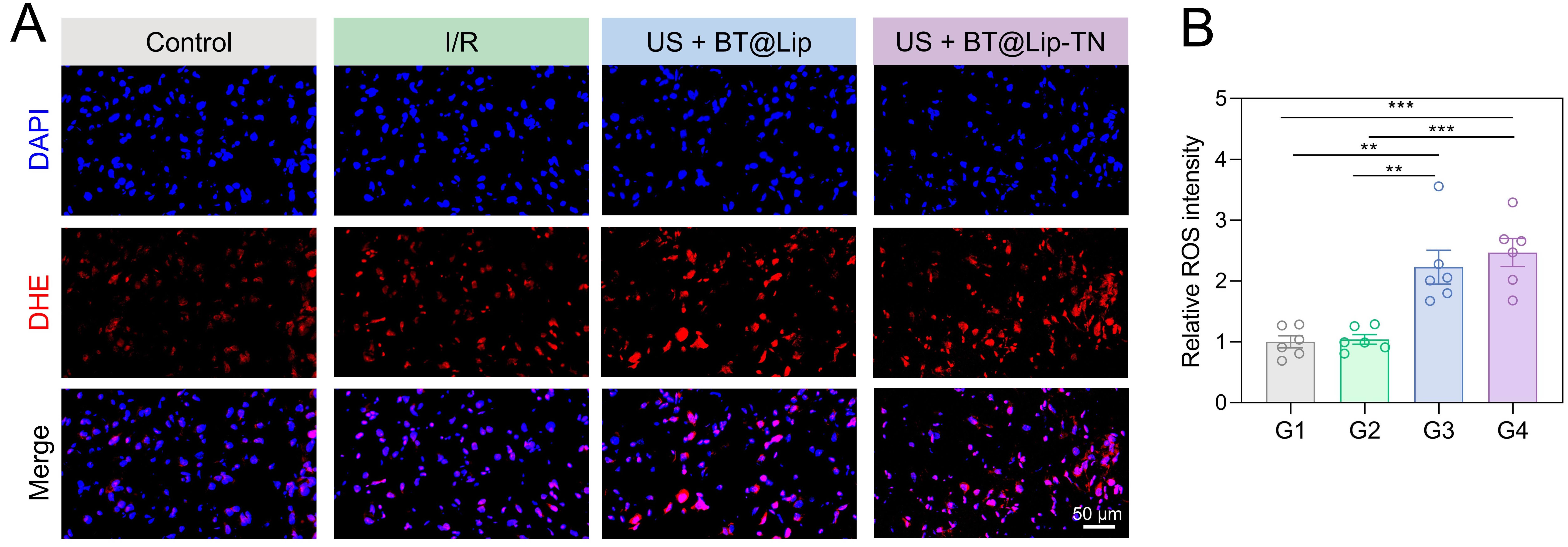


**Figure S31.** A) Evaluation of ROS generation in the PVN region by DHE staining. Scale bar: 50 μm. B) Statistical analysis of the relative fluorescence intensity. Data are presented as mean ± S.E.M. (*n = 6*). ***P* < 0.01, and ****P* < 0.001.


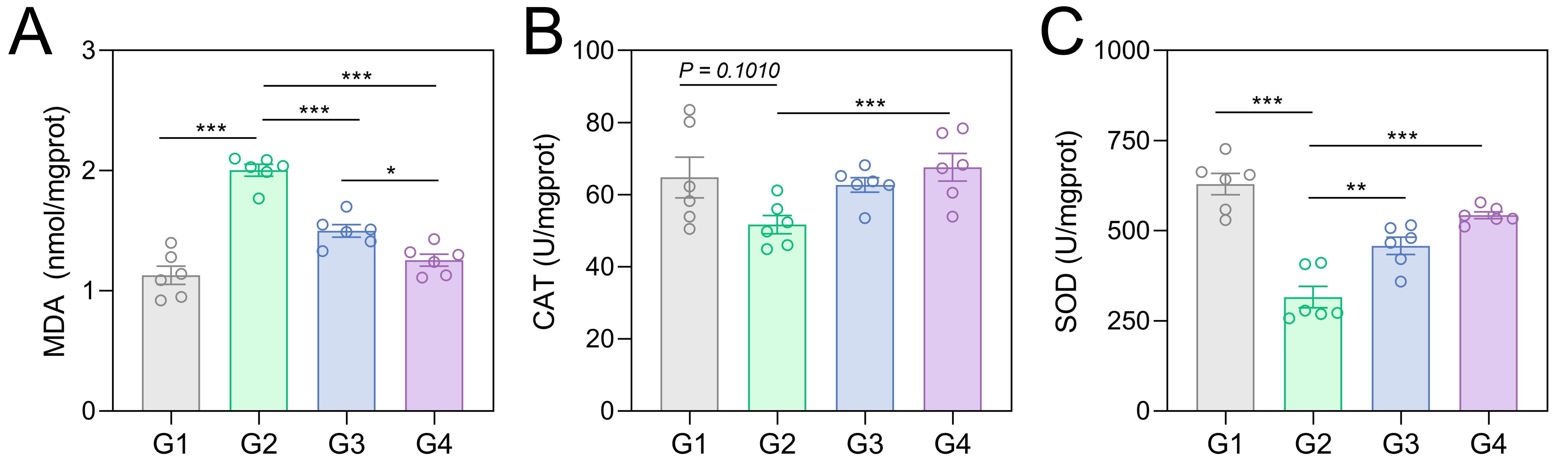


**Figure S32.** Levels of A) MDA, B) CAT, and C) SOD in the peri-ischemic myocardium. Data are expressed as mean ± S.E.M. (*n = 6*). **P* < 0.05, ***P* < 0.01, and ****P* < 0.001.


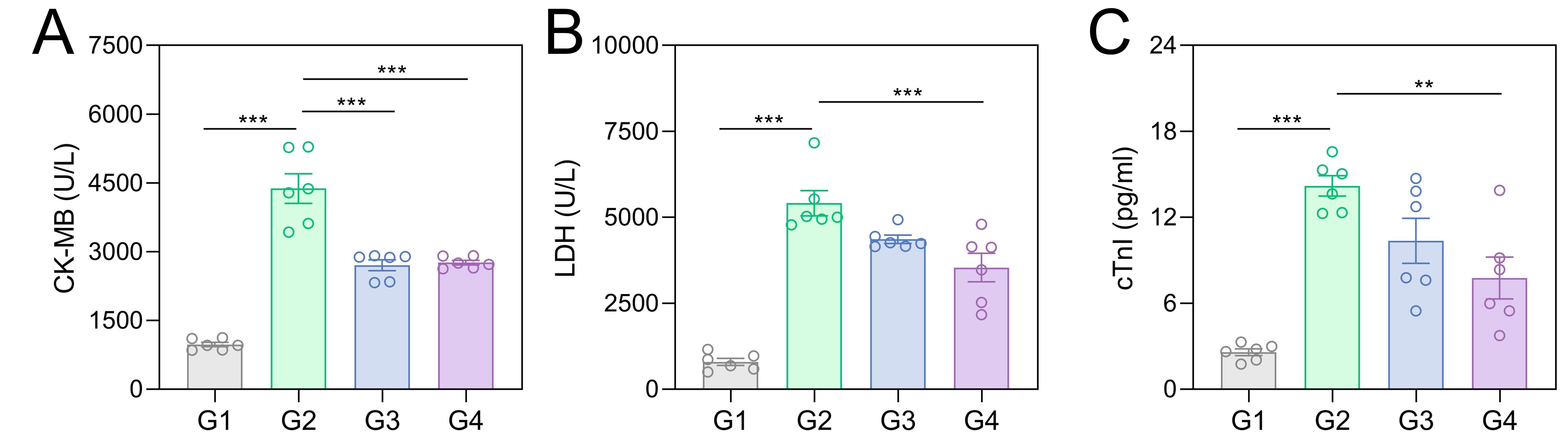


**Figure S33.** Levels of cardiac injury biomarkers including serum A) CK-MB, B) LDH, and C) cTnI. Values are expressed as mean ± S.E.M. (*n = 6*). ***P* < 0.01, and ****P* < 0.001.


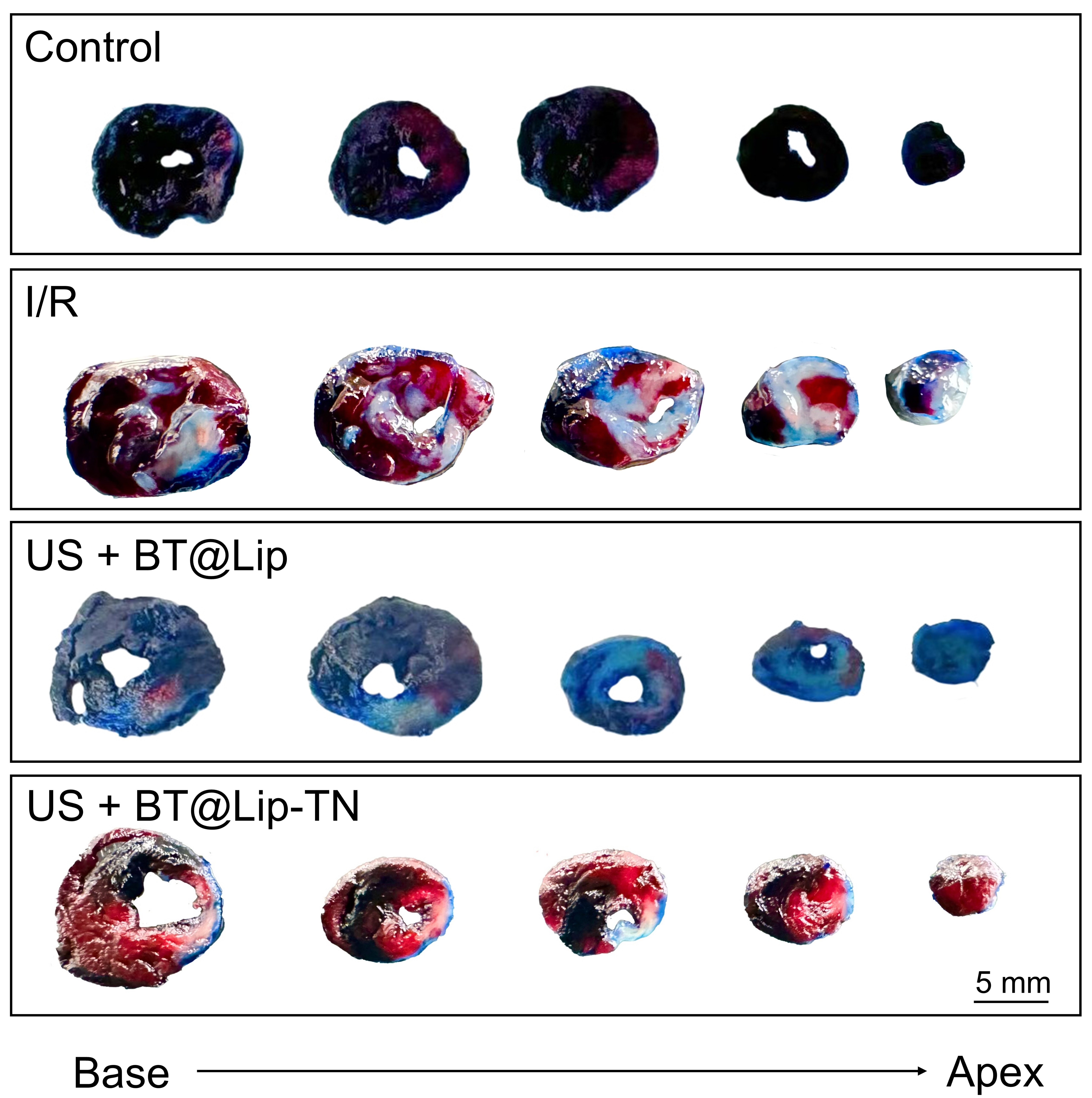


**Figure S34.** Representative images of myocardial Evans blue-TTC staining. Scale bar: 5 mm.


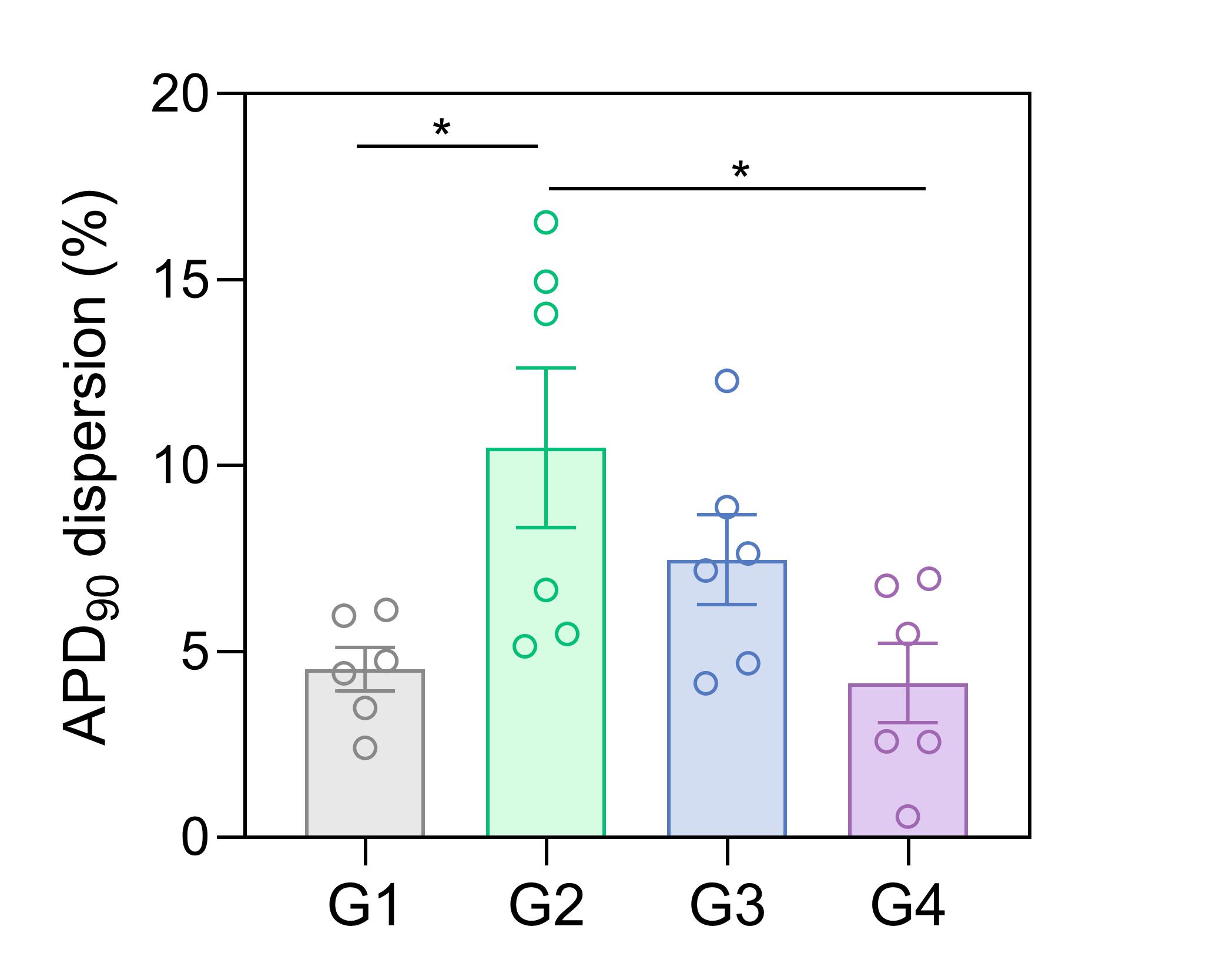


**Figure S35.** Statistical analysis of APD_90_ dispersion. Data are expressed as mean ± S.E.M. (n = 6). **P* < 0.05.


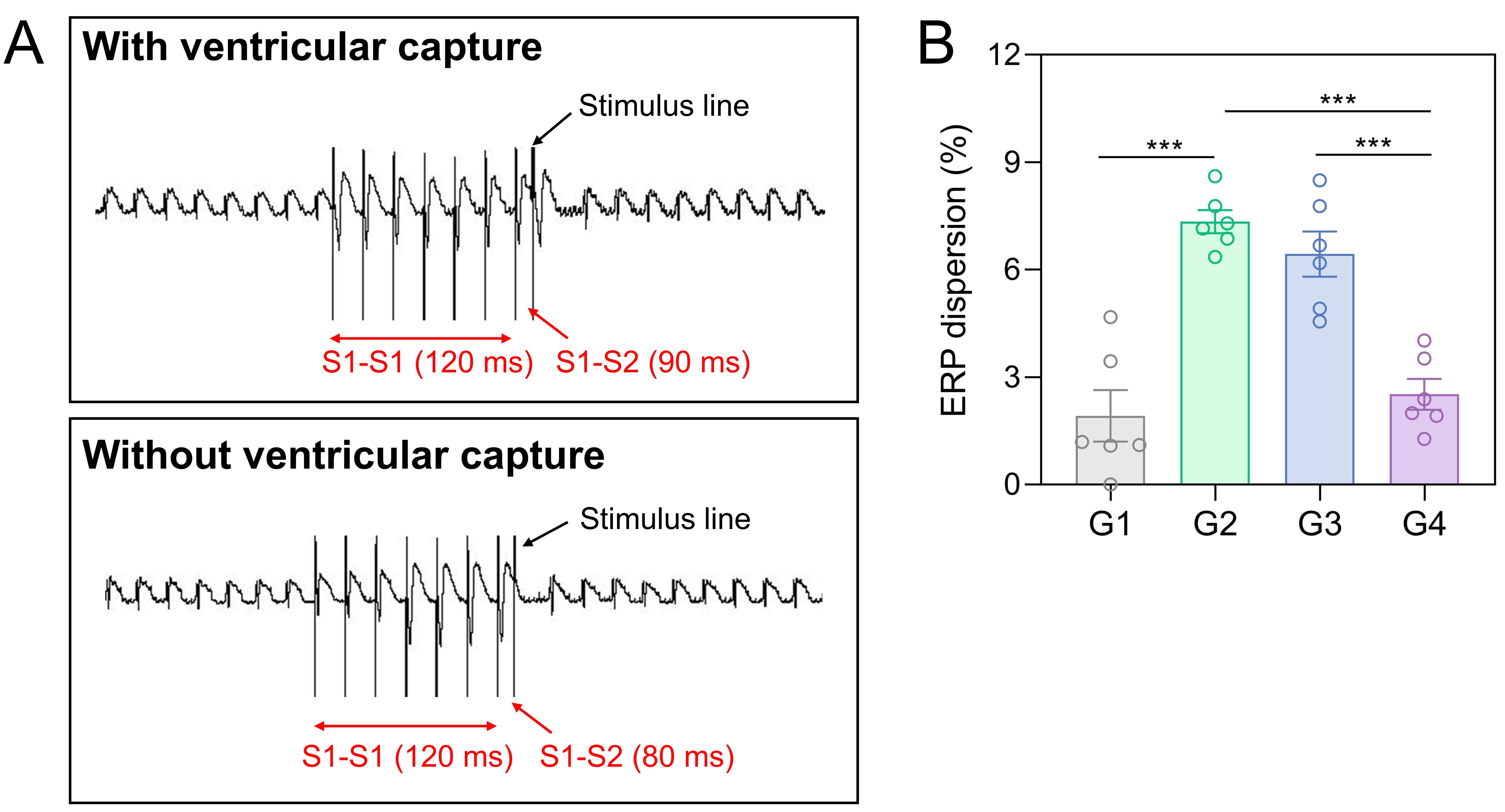


**Figure S36.** A) Representative ECG showing ventricular capture during the determination of ventricular ERP. B) Statistical analysis of ERP dispersion. Data are expressed as mean ± S.E.M. (*n = 6*). ****P* < 0.001.


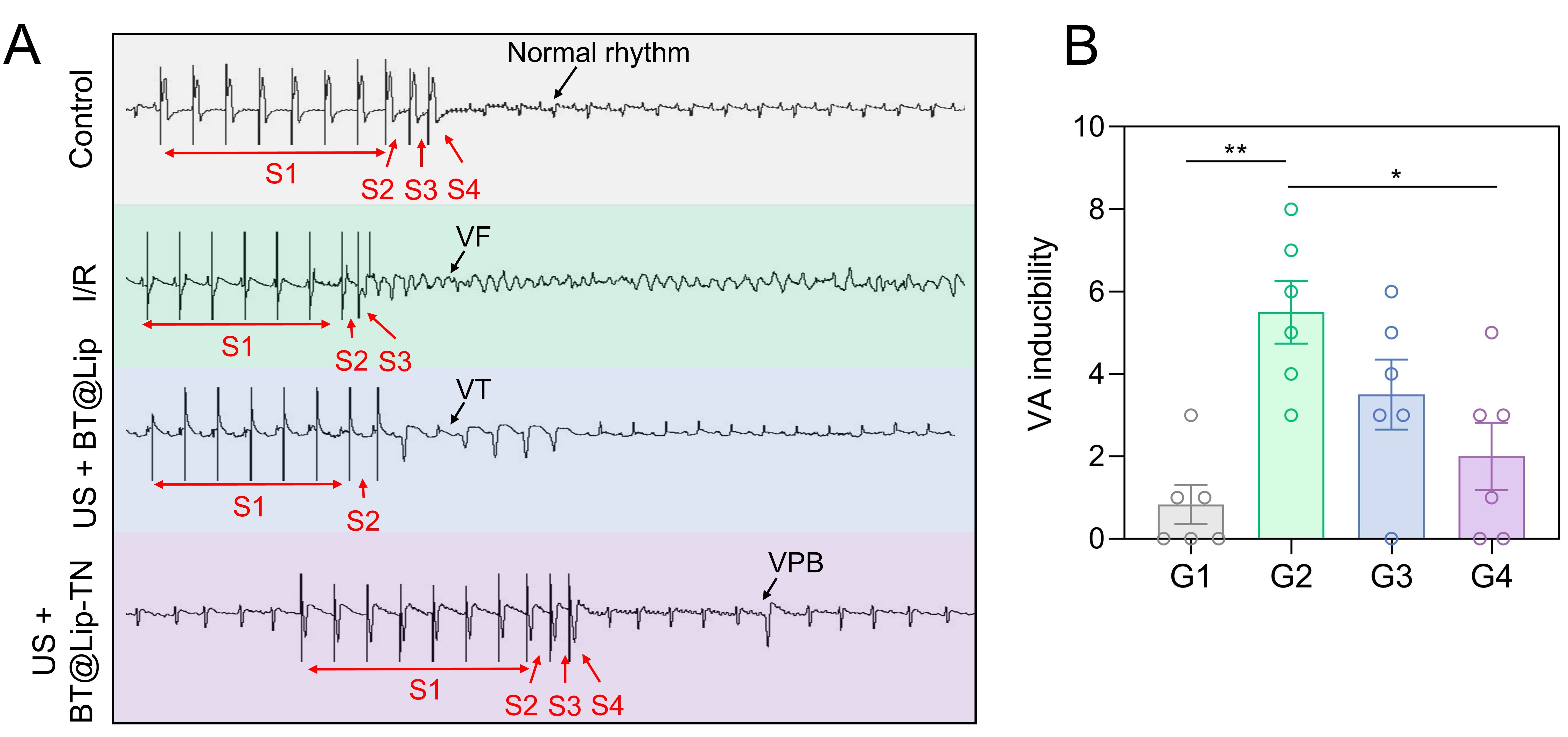


**Figure S37.** A) Representative images of programmed electrical stimulation for measuring VA inducibility. B) Statistical analysis of VA inducibility in the four groups. Data are presented as mean ± S.E.M. (*n = 6*). **P* < 0.05, and ***P* < 0.01.


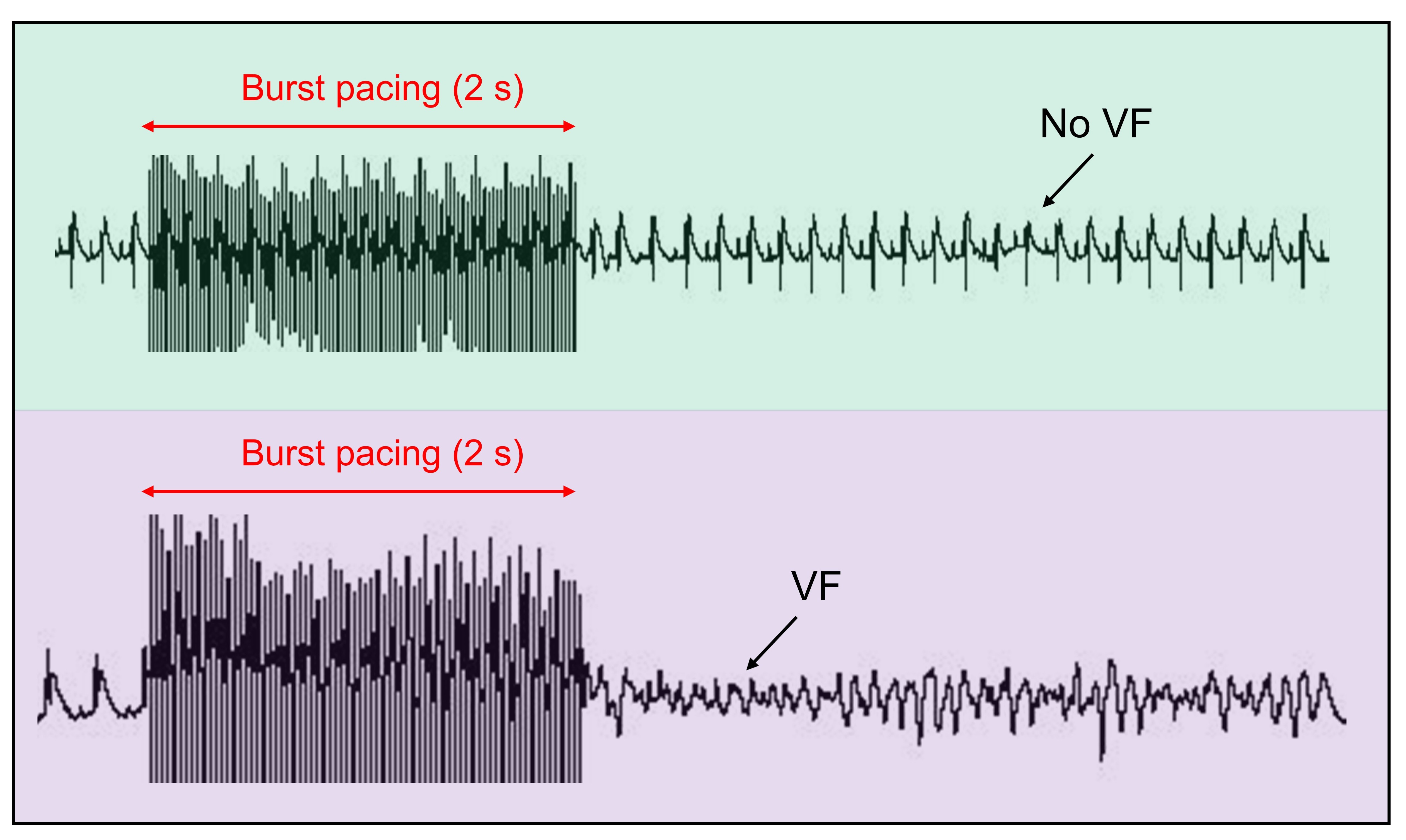


**Figure S38.** Representative ECG recordings during burst pacing (for 2 s) to detect ventricular fibrillation (VF) threshold.


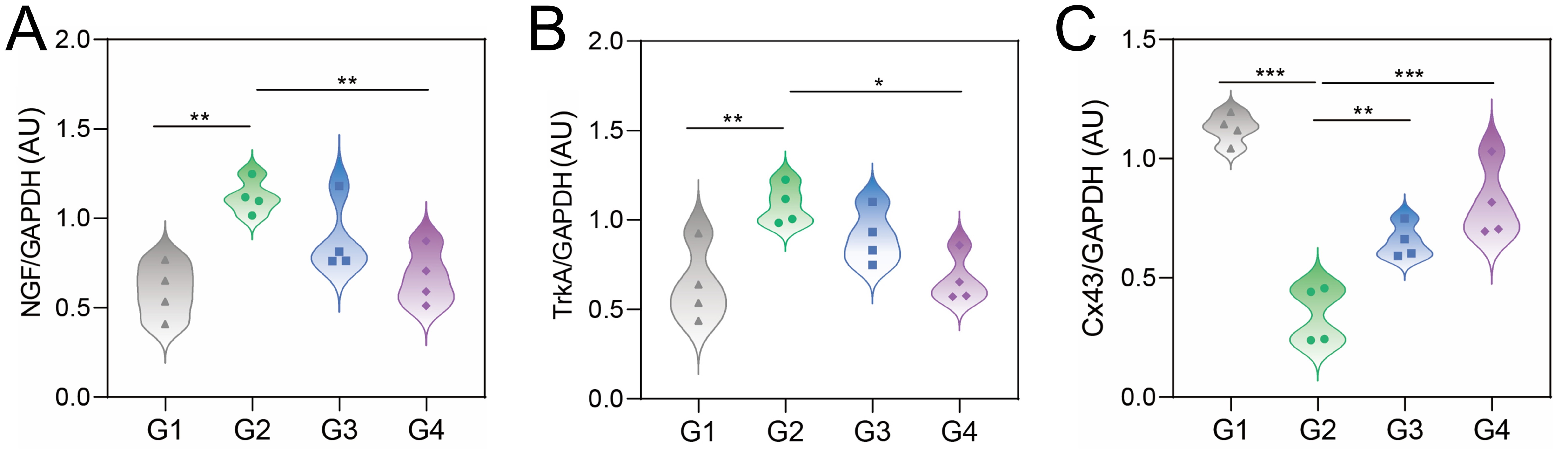


**Figure S39.** Semi-quantification of relative expression of A) NGF, B) TrkA, and C) Cx43 (normalized to GAPDH). Data are presented as mean ± S.E.M. (*n = 4*). **P* < 0.05, ***P* < 0.01, and ****P* < 0.001.


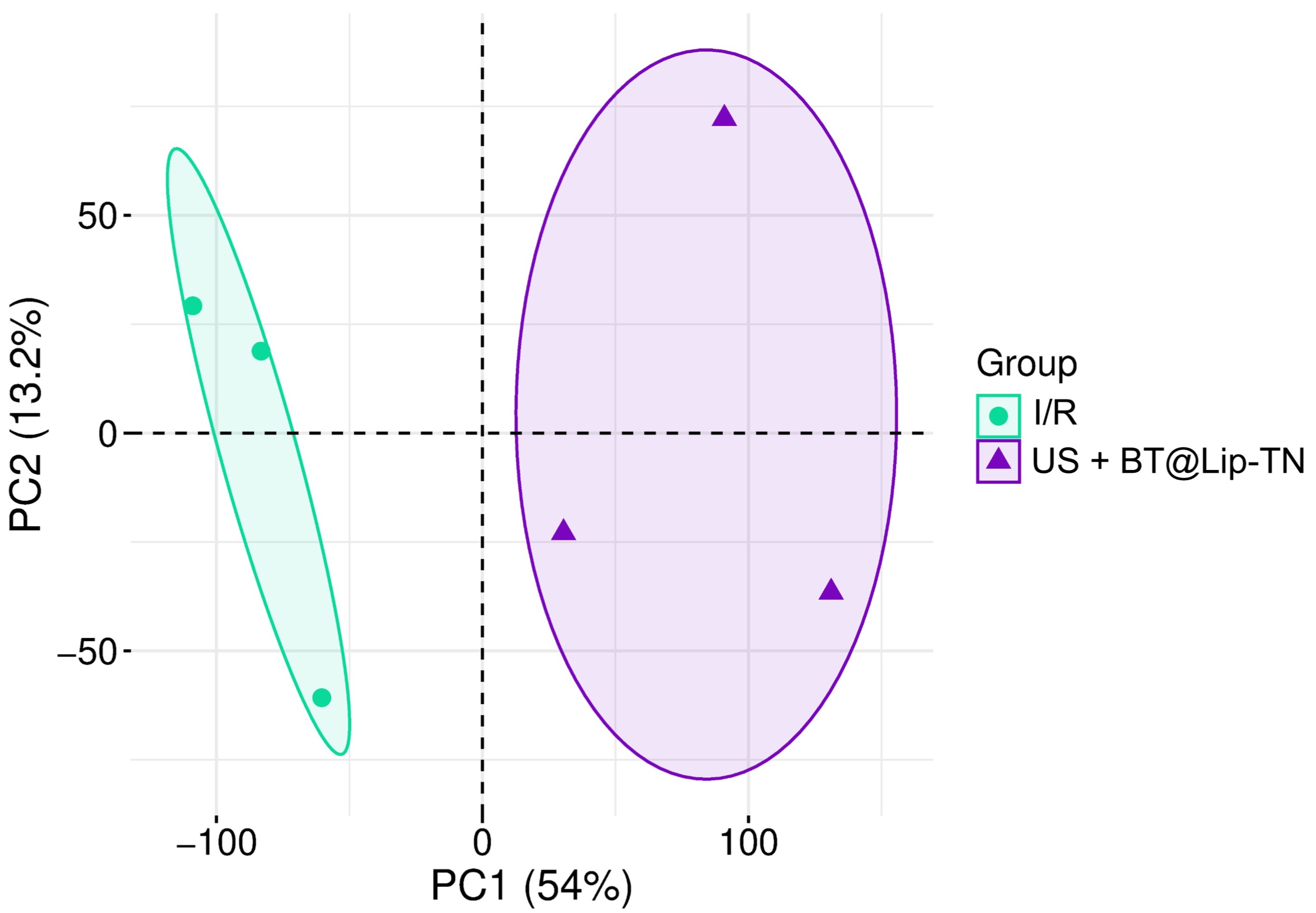


**Figure S40.** Principal component analysis (PCA) based on RNA-sequencing results from the I/R group and US + BT@Lip-TN group (*n = 3*).


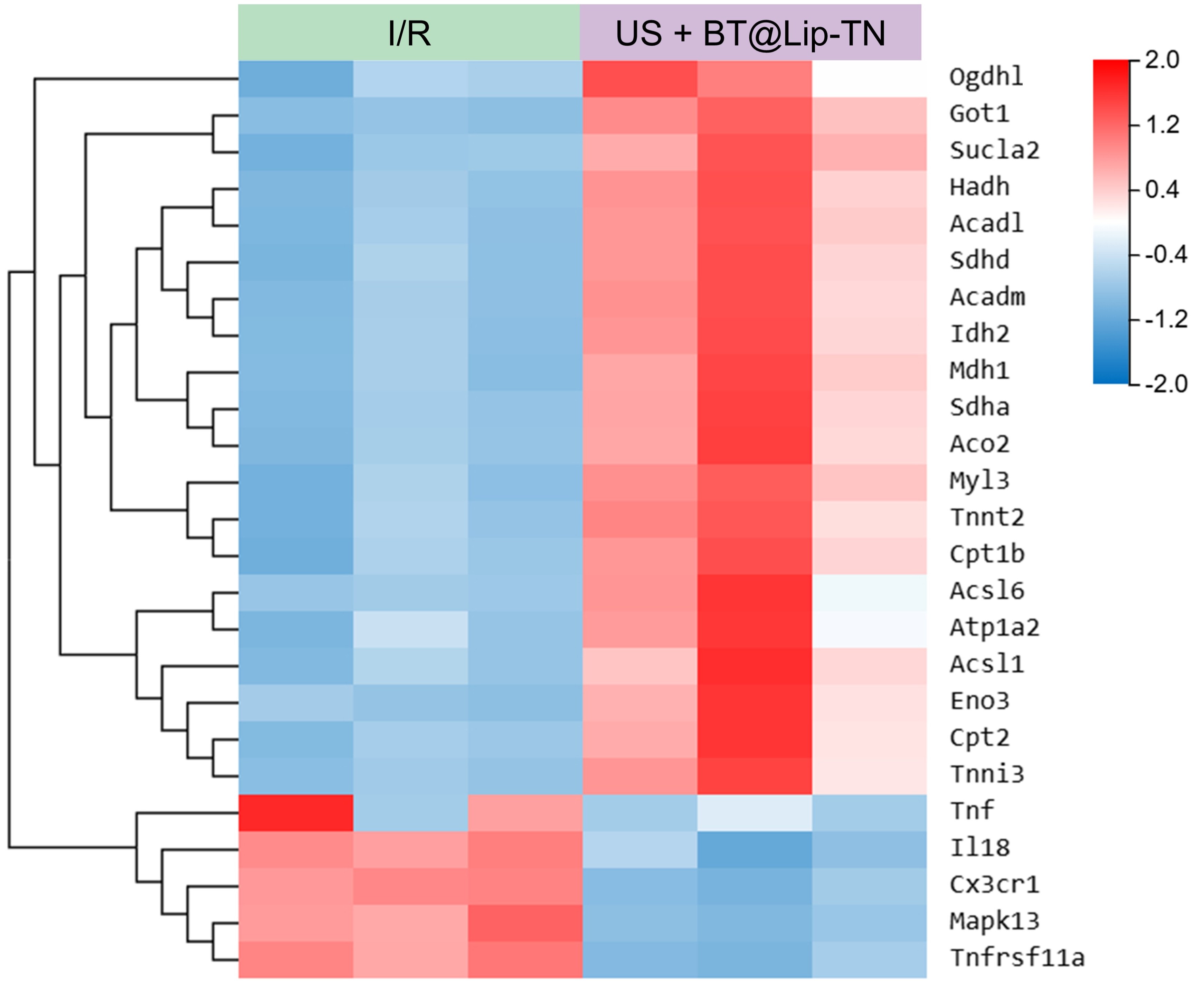


**Figure S41.** Heatmap showing the relative expression levels of key genes involved in pathways including "Carbon metabolism", "Fatty acid metabolism", "IL-17 signaling pathway", "Cardiac muscle contraction", "Citrate cycle (TCA cycle)", and "Cytokine-cytokine receptor interaction".


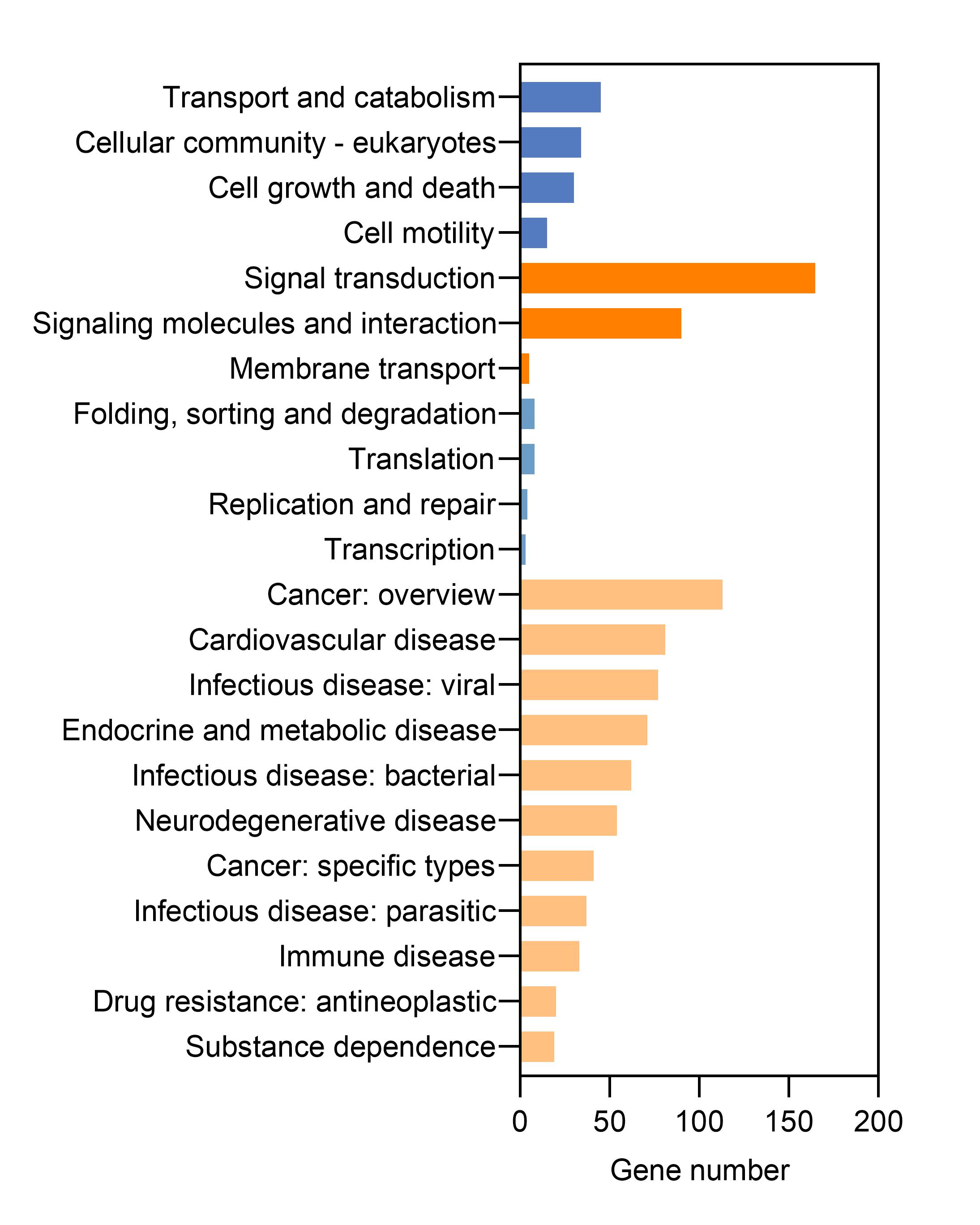


**Figure S42.** Changes in KEGG pathway classification.


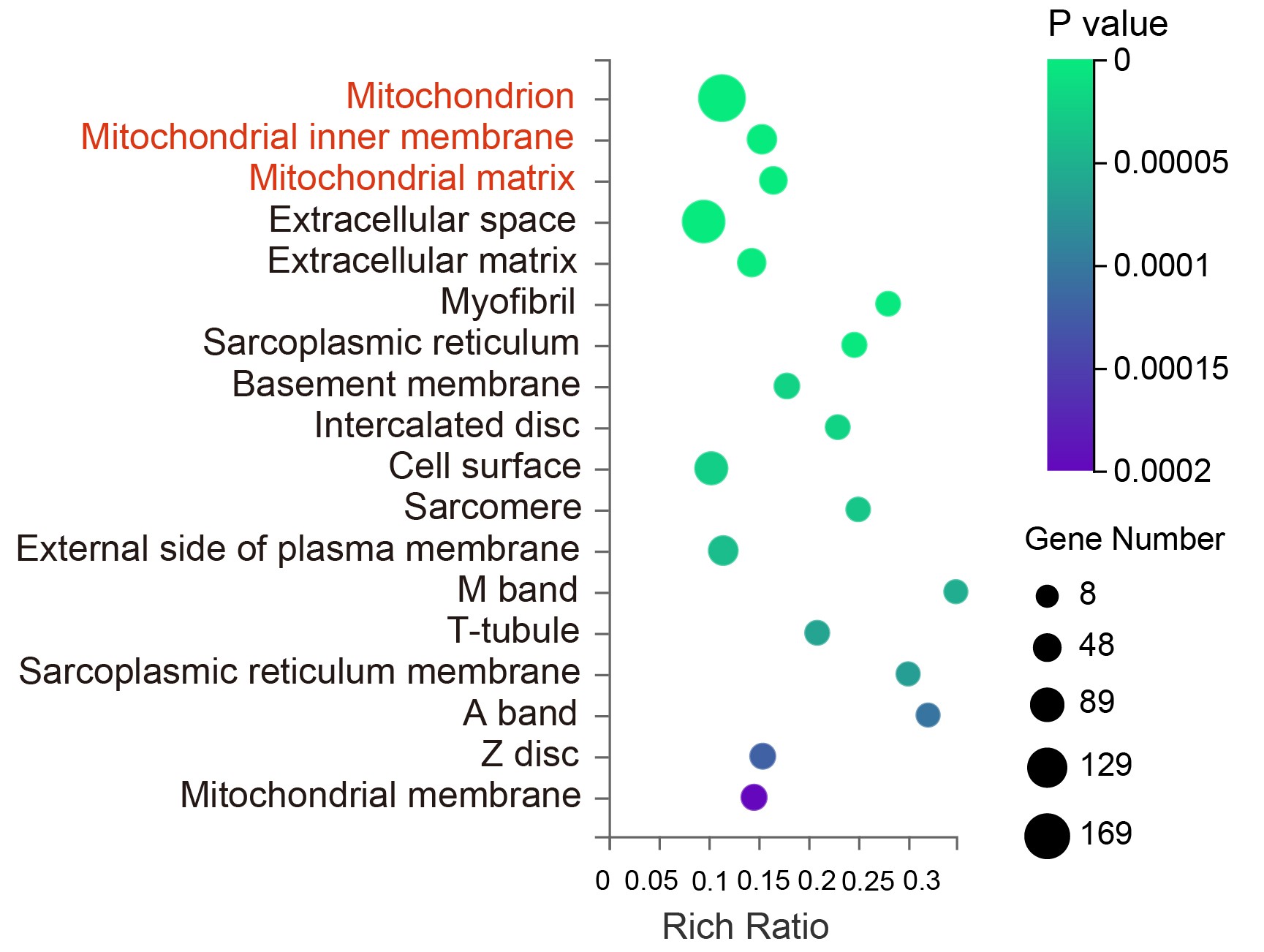


**Figure S43.** Bubble plot of Gene Ontology (GO) pathway enrichment analysis.


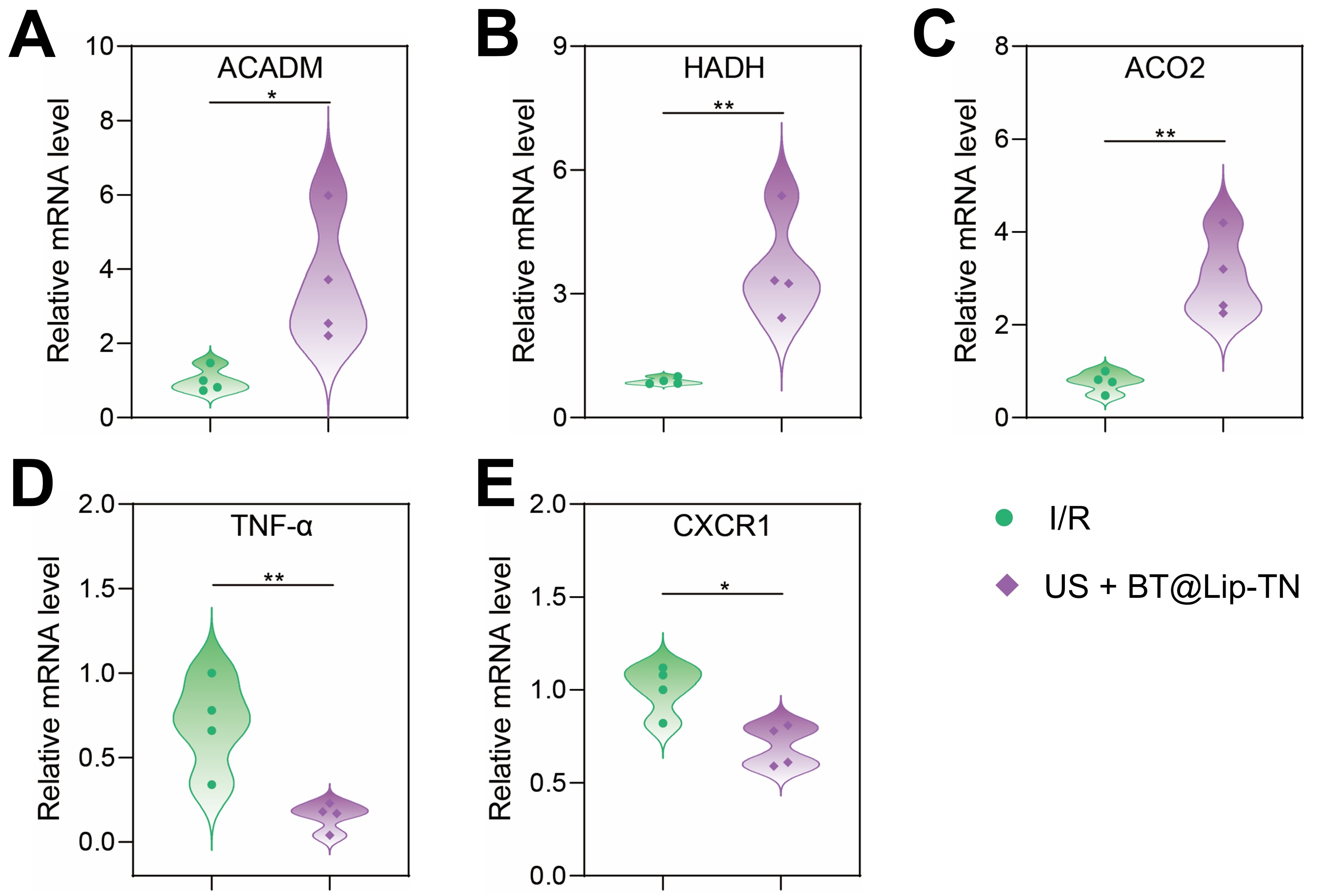


**Figure S44.** Relative mRNA expression levels of A) ACADM, B) HADH, C) ACO2, D) TNF-α, and E) CXCR1 in peri-ischemic myocardial tissue. Data are presented as mean ± S.E.M. (*n = 4*). **P* < 0.05, and ***P* < 0.01.


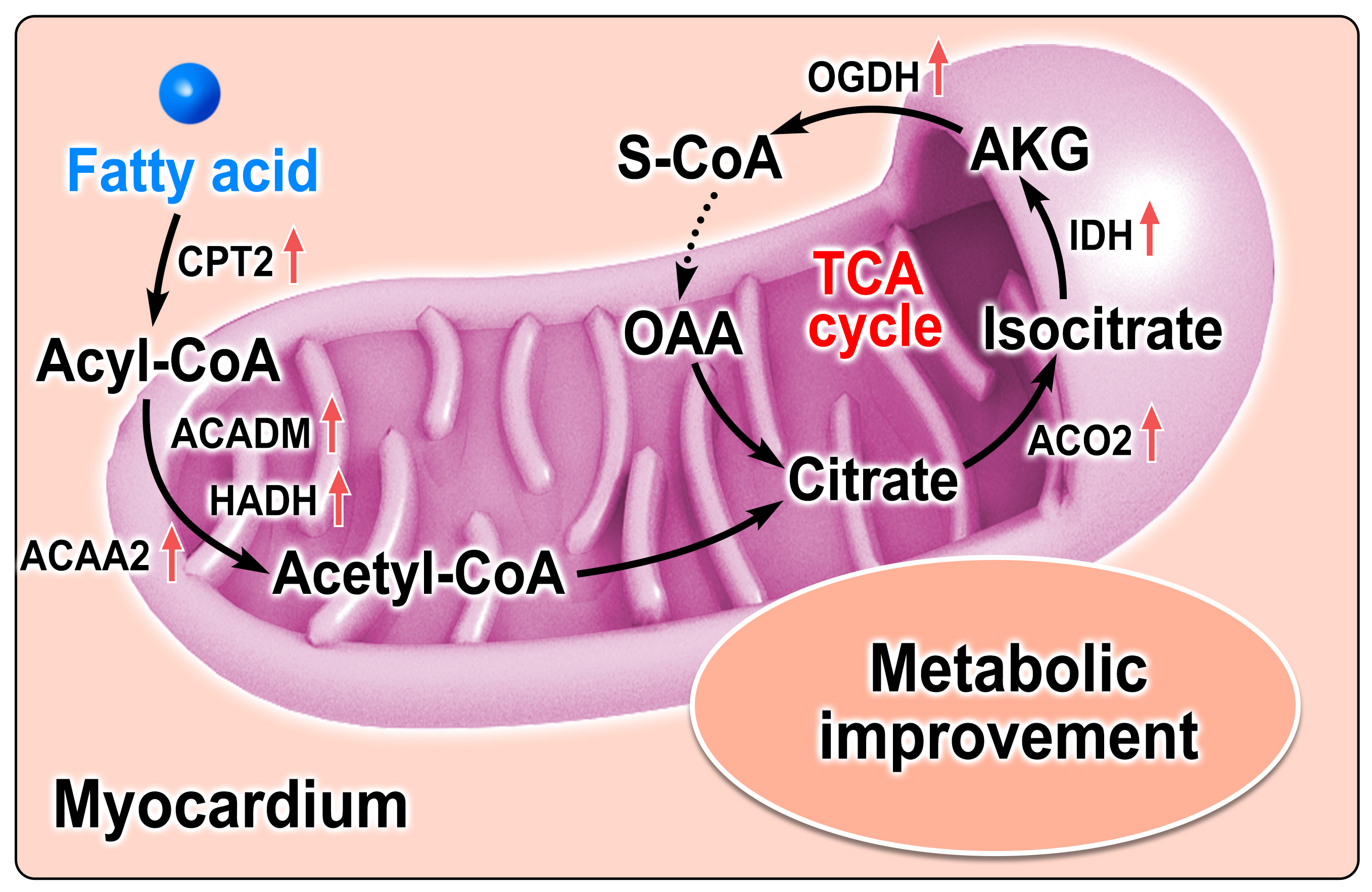


**Figure S45.** Schematic diagram illustrating the mechanism by which US + BT@Lip-TN improves myocardial metabolism. CPT2, carnitine palmitoyltransferase 2; Acyl-CoA, acyl-coenzyme A; ACADM, acyl-CoA dehydrogenase medium chain; HADH, hydroxyacyl-CoA dehydrogenase; ACAA2, acetyl-CoA acyltransferase 2; Acetyl-CoA, acetyl coenzyme A; ACO2, aconitase 2; IDH, isocitrate dehydrogenase; AKG, α-ketoglutarate; OGDH, oxoglutarate dehydrogenase; S-CoA, succinyl-coenzyme A; OAA, oxaloacetate.


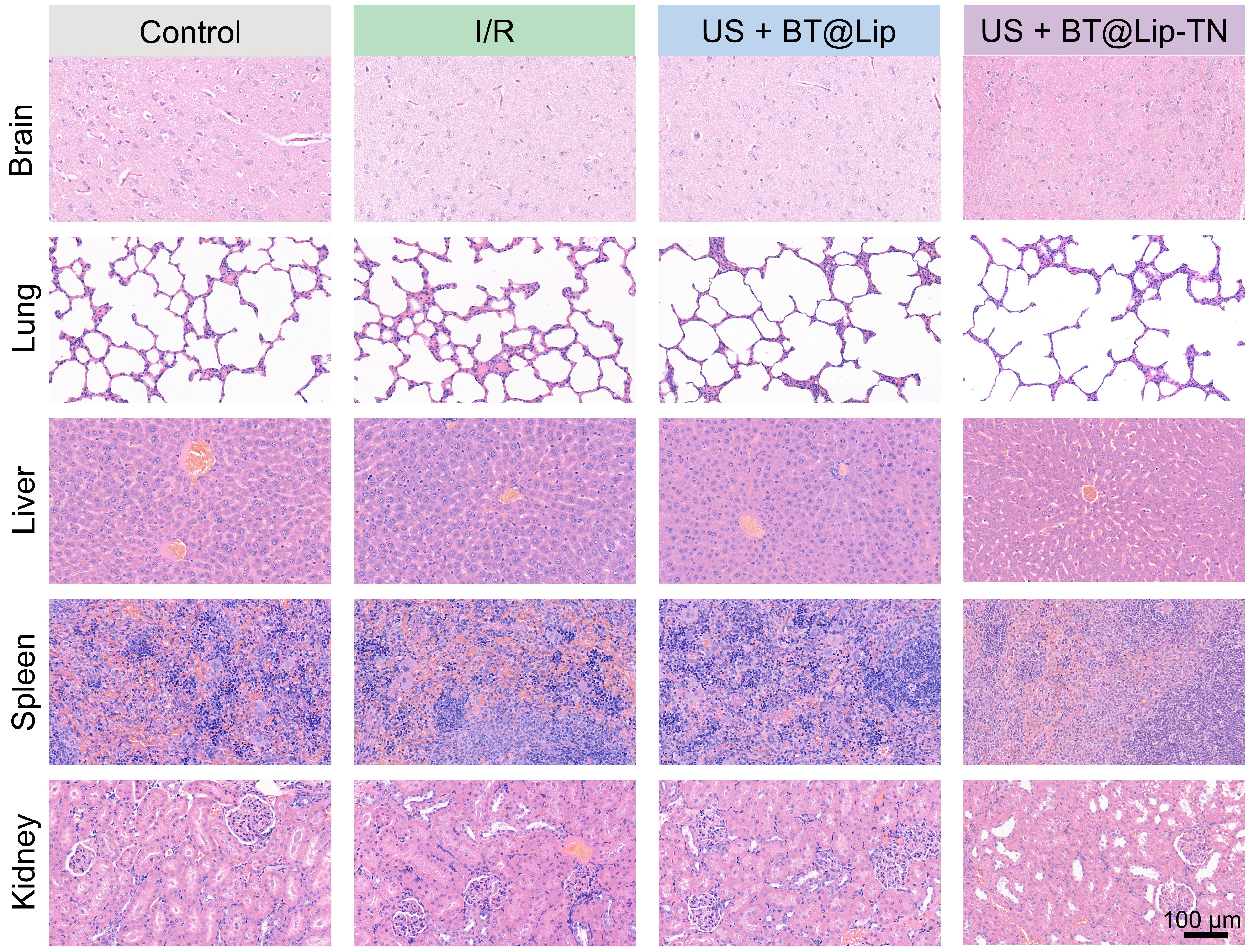


**Figure S46.** H&E staining images of major organs under different treatments. Scale bar: 100 μm.


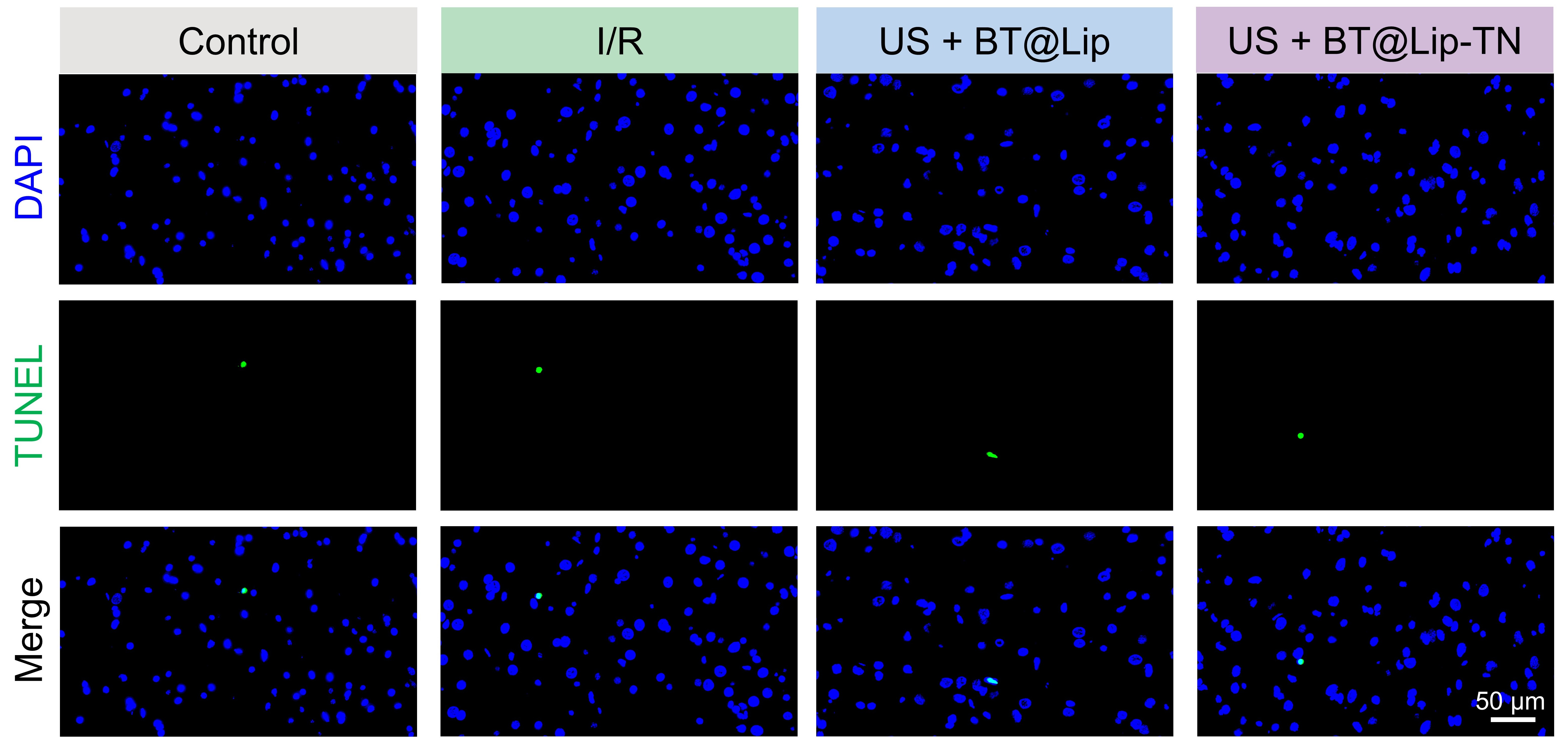


**Figure S47.** Representative TUNEL staining images of the PVN region in the four experimental groups. Scale bar: 50 μm.


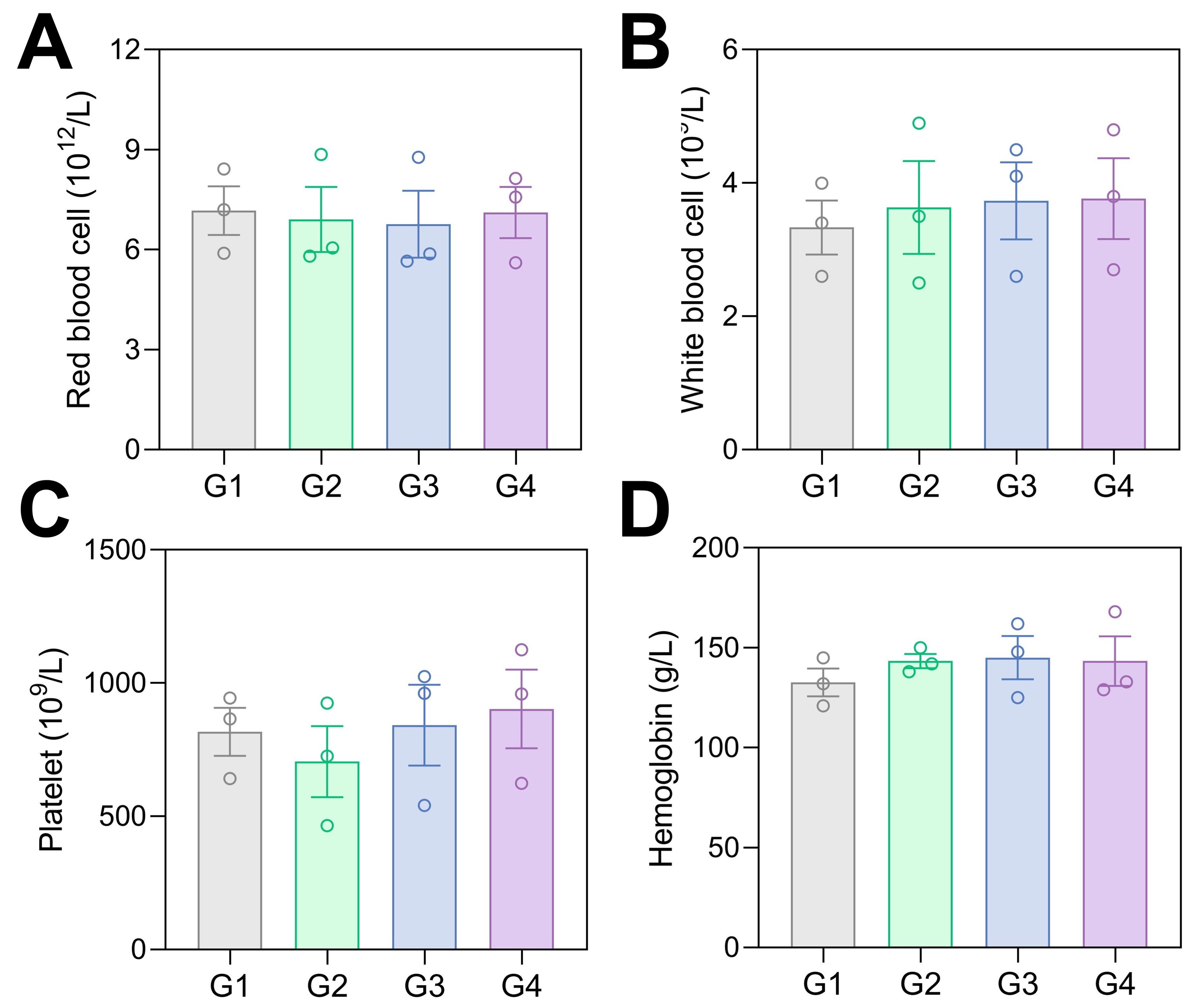


**Figure S48.** A) Red blood cell, B) white blood cell, C) platelet, and D) hemoglobin counts in rats from the four groups. Data are presented as mean ± S.E.M. (*n = 3*). All values fell within normal reference ranges.


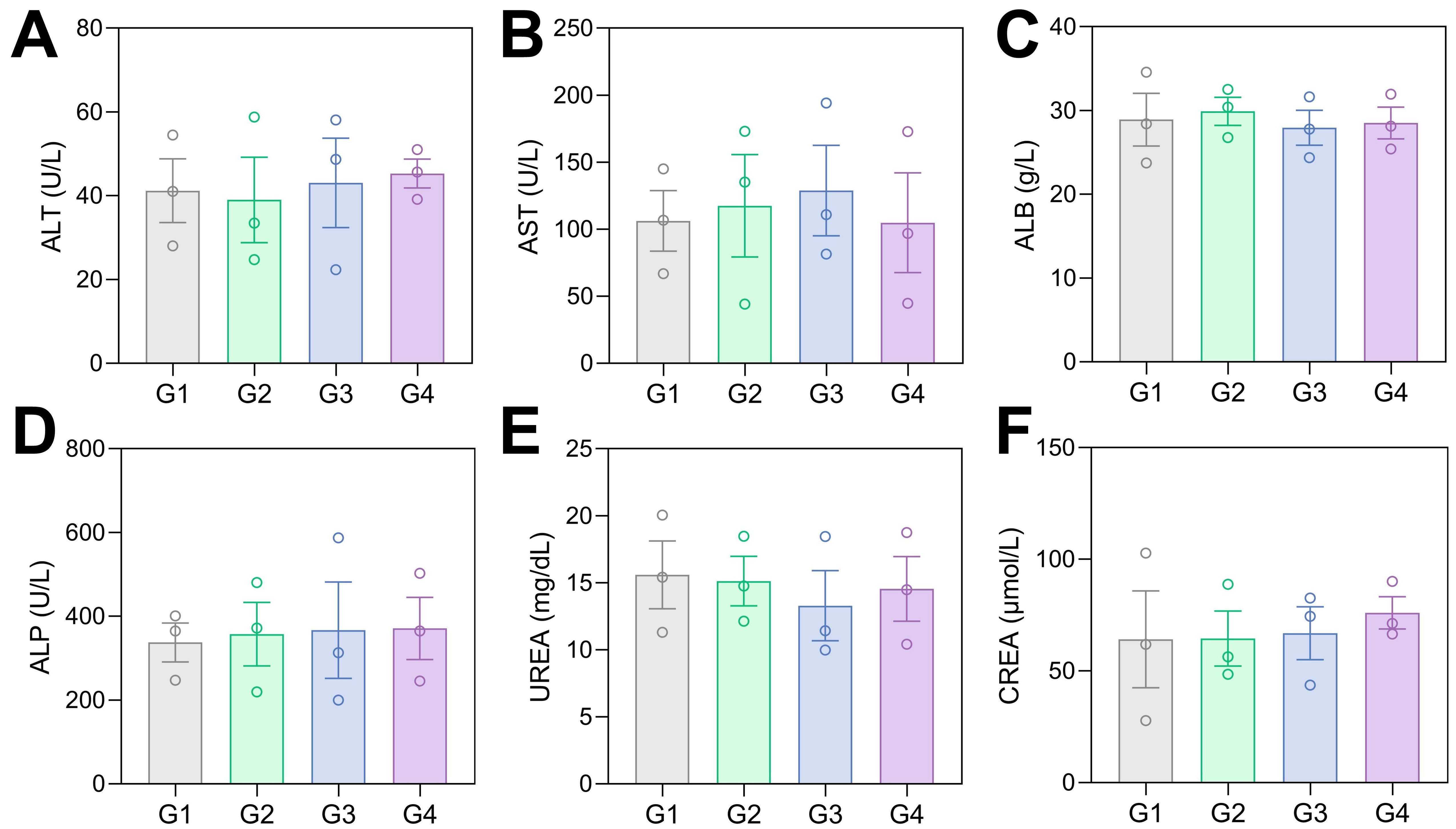


**Figure S49.** Levels of A) alanine aminotransferase (ALT), B) aspartate aminotransferase (AST), C) albumin (ALB), D) alkaline phosphatase (ALP), E) urea (UREA), and F) Creatinine (CREA) in rats across different treatment groups. Data are expressed as mean ± S.E.M. (*n = 3*). All values remained within normal reference ranges.


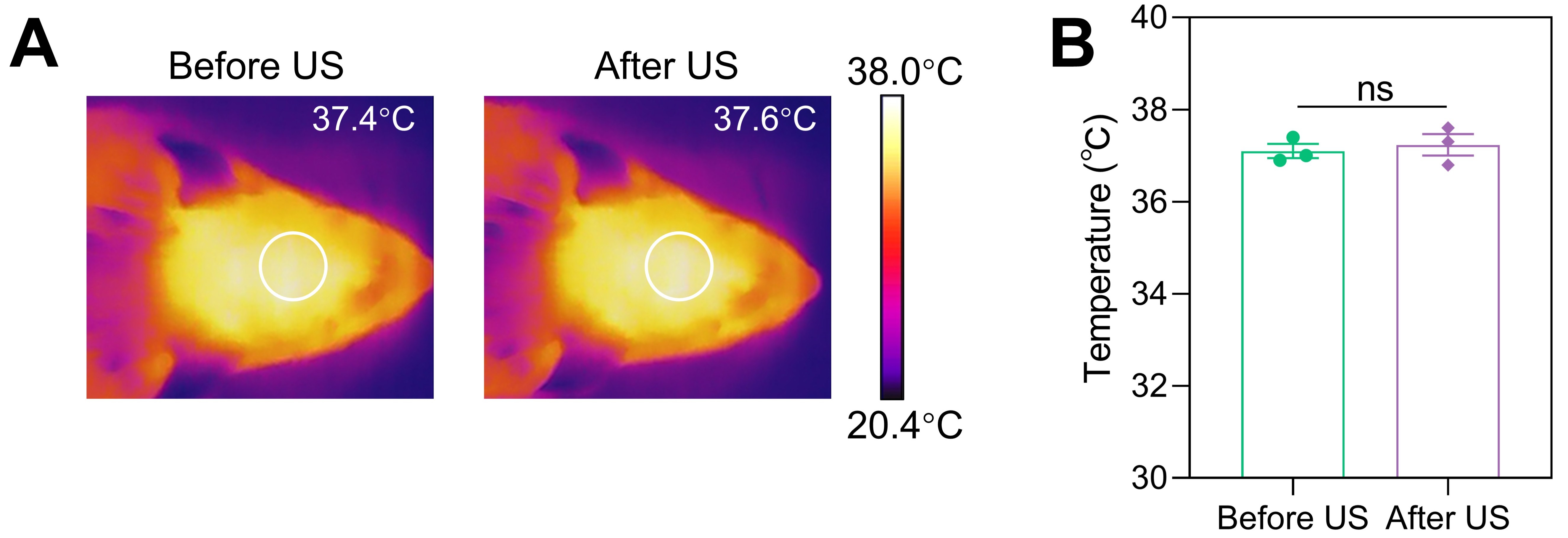


**Figure S50.** Temperature changes of the skull surface before and after ultrasound irradiation (1.0 MHz, 1.5 W cm^-2^, 15 min). Data are presented as mean ± S.E.M. (*n = 3*). Ns, no significance.

**4. Supplementary Tables**

**Table S1.** Loading ratio and encapsulation efficiency analysis of BT@Lip-TN.

| Liposomes: BT (W/W) | Loading ratio | Encapsulation efficiency |
| --- | --- | --- |
| 50:1 | 1.4% | 70% |
| 50:2 | 2.0% | 50% |
| 50:5 | 3.8% | 40% |
| 50:10 | 4.2% | 22% |

**Table S2.** Ultrasound parameters utilized in the *in vivo* study.

| Parameters | Value |
| --- | --- |
| Frequency | 1.0 MHz |
| Intensity | 1.5 W cm^-2^ |
| Pulse repetition frequency (PRF) | 100 Hz |
| Duty cycle (DC) | 50% |
| Duration | 15 min |

**Table S3.** The primer sequences for qPCR.

| Gene | Forward primer | Reverse primer |
| --- | --- | --- |
| GAPDH | CTGGAGAAACCTGCCAAGTATG | GGTGGAAGAATGGGAGTTGCT |
| OGDH | TCCGTGCCCGCTGACATTAT | TGGCATTGCTCCAAATCGTTA |
| ACO2 | CGAGGACATGGGACGATCAG | AACGATTCCACCCACATCCC |
| IDH2 | CCCATCACCATTGGCAGACAC | CCTCCGGCAGGGAAGTTATACA |
| ACADM | AGGCTACAAGGTCCTGAGAAGTG | CTTTTATCGTAGTCTGGGGCGA |
| HADH | GAGGACATCCTGGCAAAATCC | CTGGTTGAAAGGGAGCTCAGG |
| ACAA2 | ATGTGGGTTTACGAGTGGGAGTC | TTCCTCCACATAAGACGACCTCAG |
| CPT2 | GCCCACCATGCACTACCA | TCCCAACGCCAGTCTCAAA |
| TNF-α | CCACCACGCTCTTCTGTCTACTG | TGGGCTACGGGCTTGTCACT |
| IL-18 | AACAGCCAACGAATCCCAGAC | TTGTTTTTACAGGAGAGGGTAGACA |
| CXCR1 | CTCTCTTAGGAGCCCACTTGATTC | CCAACAAAGGCATAGATGACGG |

Abbreviations: GAPDH, glyceraldehyde-3-phosphate dehydrogenase; OGDH, oxoglutarate dehydrogenase; ACO2, aconitase 2; IDH2, isocitrate dehydrogenase 2; ACADM, acyl-CoA dehydrogenase medium chain; HADH, hydroxyacyl-CoA dehydrogenase; ACAA2, acetyl-coenzyme A acyltransferase 2; CPT2, carnitine palmitoyltransferase 2; TNF-α, tumor necrosis factor-α; IL-18, interleukin-18; CXCR1, C-X-C motif chemokine receptor 1.

**5. Supplementary References**

[1] H. Hu, S. Wang, Q. Li, J. Zhao, Y. Pang, J. Wang, H. Wu, X. Wang, Y. Cheng, M. Yu, X. Yin, Y. Zhang, L. Yu, Y. Sun, H. Jiang, Autophagy-enhanced nanosonosensitizer mediated sonodynamic therapy for post-myocardial infarction neuromodulation and arrhythmia prevention, Theranostics, 15 (2025) 2201-2214.

[2] H. Xiang, C. Yan, Y. Lei, N. Lai, P. Zhang, K. Jiang, W. Zhao, H. Yang, W. Cui, Y. Li, Aging‐Regulating Microspheres for Enhancing Mitochondrial Biogenesis in Early Senescence and Clearing Late Senescent Cells, Adv. Funct. Mater., 35 (2025) e06677.

[3] X. Zhang, C. Li, Y. Zhang, X. Guan, L. Mei, H. Feng, J. Li, L. Tu, G. Feng, G. Deng, Y. Sun, Construction of Long‐Wavelength Emissive Organic Nanosonosensitizer Targeting Mitochondria for Precise and Efficient In Vivo Sonotherapy, Adv. Funct. Mater., 32 (2022) 2207259.

[4] J. Zhao, Q. Zhang, W. Cheng, Q. Dai, Z. Wei, M. Guo, F. Chen, S. Qiao, J. Hu, J. Wang, H. Chen, X. Bao, D. Mu, X. Sun, B. Xu, J. Xie, Heart–gut microbiota communication determines the severity of cardiac injury after myocardial ischaemia/reperfusion, Cardiovasc Res, 119 (2023) 1390-1402.

[5] L. Yu, L. Zhou, G. Cao, S.S. Po, B. Huang, X. Zhou, M. Wang, S. Yuan, Z. Wang, S. Wang, H. Jiang, Optogenetic Modulation of Cardiac Sympathetic Nerve Activity to Prevent Ventricular Arrhythmias, J. Am. Coll. Cardiol., 70 (2017) 2778-2790.

[6] J.P. Schütte, M.-C. Manke, K. Hemmen, P. Münzer, B.F. Schörg, G.C. Ramos, M. Pogoda, V. Dicenta, S.H.L. Hoffmann, J. Pinnecker, F. Kollotzek, M. Zdanyte, K.A.L. Mueller, Y. Singh, A.F. Mack, B. Pichler, F. Lang, B. Nieswandt, M. Gawaz, K.G. Heinze, N. Casadei, O. Borst, Platelet-Derived MicroRNAs Regulate Cardiac Remodeling After Myocardial Ischemia, Circ. Res., 132 (2023) e96-e113.

[7] P. Švorc, S. Grešová, P. Švorc, Heart rate variability in male rats, Physiol Rep., 11 (2023) e15827.

[8] L. Zhou, Y. Zhang, G. Cao, C. Zhang, C. Zheng, G. Meng, Y. Lai, Z. Zhou, Z. Liu, Z. Liu, F. Guo, X. Dong, Z. Liang, Y. Wang, S. Guo, X. Zhou, H. Jiang, L. Yu, Wireless Self‐Powered Optogenetic System for Long‐Term Cardiac Neuromodulation to Improve Post‐MI Cardiac Remodeling and Malignant Arrhythmia, Adv. Sci., 10 (2023) e2205551.

[9] W. Tan, S. Cheng, Q. Qiu, J. Huang, M. Xie, L. Song, Z. Zhou, Y. Wang, F. Guo, X. Jin, Z. Li, X. Xu, H. Jiang, X. Zhou, Celastrol exerts antiarrhythmic effects in chronic heart failure via NLRP3/Caspase-1/IL-1β signaling pathway, Biomed. Pharmacother., 177 (2024) 117121.

[10] C. Xiang, Y. Cheng, X. Yu, T. Mao, H. Luo, H. Hu, Y. Wu, R. Sang, Z. Wang, Y. Wang, Q. Luo, J. Huang, J. Zhao, J. Wang, X. Wang, M. Chen, W. Liu, L. Zhou, S. Wang, H. Jiang, Low-intensity focused ultrasound modulation of the paraventricular nucleus to prevent myocardial infarction–induced ventricular arrhythmia, Heart Rhythm, 21 (2024) 340-348.

[11] E.R. Barrozo, M.D. Seferovic, M.P. Hamilton, D.N. Moorshead, M.D. Jochum, T. Do, D.S. O’Neil, M.A. Suter, K.M. Aagaard, Zika virus co-opts microRNA networks to persist in placental niches detected by spatial transcriptomics, Am J Obstet Gynecol., 230 (2024) 251.e251-251.e217.
